# Label-free SRM-based relative quantification of antibiotic resistance mechanisms in *Pseudomonas aeruginosa* clinical isolates

**DOI:** 10.3389/fmicb.2015.00081

**Published:** 2015-02-10

**Authors:** Yannick Charretier, Thilo Köhler, Tiphaine Cecchini, Chloé Bardet, Abdessalam Cherkaoui, Catherine Llanes, Pierre Bogaerts, Sonia Chatellier, Jean-Philippe Charrier, Jacques Schrenzel

**Affiliations:** ^1^Genomic Research Laboratory, Service of Infectious Diseases, Geneva University HospitalsGeneva, Switzerland; ^2^Department of Microbiology and Molecular Medicine, University of GenevaGeneva, Switzerland; ^3^Institute for Analytical Sciences, Joint Research Unit 5280 CNRS/Lyon 1 UniversityVilleurbanne, France; ^4^Technology Research Department, BioMérieux SAMarcy l'Etoile, France; ^5^UMR1092 INSERM, Limoges UniversityLimoges, France; ^6^MD3, BioMérieux SAMarcy l'Etoile, France; ^7^Laboratory of Bacteriology, Department of Genetic and Laboratory Medicine, Geneva University HospitalsGeneva, Switzerland; ^8^Laboratoire de Bactériologie, EA4266, Université de Franche-ComtéBesançon, France; ^9^Laboratoire de Microbiologie, CHU Dinant-Godinne UCL NamurYvoir, Belgique; ^10^Microbiology Unit, BioMérieux SALa Balme Les Grottes, France

**Keywords:** SRM, *Pseudomonas aeruginosa*, multidrug efflux system, AmpC cephalosporinase, OprD porin, carbapenem resistance, cephalosporin resistance, RT-qPCR

## Abstract

Both acquired and intrinsic mechanisms play a crucial role in *Pseudomonas aeruginosa* antibiotic resistance. Many clinically relevant resistance mechanisms result from changes in gene expression, namely multidrug efflux pump overproduction, AmpC β-lactamase induction or derepression, and inactivation or repression of the carbapenem-specific porin OprD. Changes in gene expression are usually assessed using reverse-transcription quantitative real-time PCR (RT-qPCR) assays. Here, we evaluated label-free Selected Reaction Monitoring (SRM)-based mass spectrometry to directly quantify proteins involved in antibiotic resistance. We evaluated the label-free SRM using a defined set of *P. aeruginosa* isolates with known resistance mechanisms and compared it with RT-qPCR. Referring to efflux systems, we found a more robust relative quantification of antibiotic resistance mechanisms by SRM than RT-qPCR. The SRM-based approach was applied to a set of clinical *P. aeruginosa* isolates to detect antibiotic resistance proteins. This multiplexed SRM-based approach is a rapid and reliable method for the simultaneous detection and quantification of resistance mechanisms and we demonstrate its relevance for antibiotic resistance prediction.

## Introduction

*Pseudomonas aeruginosa* is an opportunistic bacterial pathogen able to survive in diverse environments (Lyczak et al., 2000). *P. aeruginosa* infections are predominantly hospital acquired and represent a considerable therapeutic challenge (Giamarellou and Kanellakopoulou, 2008). Selection of the appropriate antibiotic drug to initiate therapy as quickly as possible is essential to optimize the clinical outcome (Bisbe et al., 1988; Micek et al., 2005). *P. aeruginosa* is intrinsically resistant to various antibiotics resulting from the combination of low outer membrane permeability, induction or derepression of AmpC β-lactamase and intrinsic or induced expression of efflux pumps (Poole, 2011). For instance, the constitutively expressed MexAB-OprM and the inducible MexXY-OprM efflux systems extrude various antibiotics from the major drug classes, thus contributing to intrinsic resistance (Masuda et al., 2000). Furthermore, during the course of antibiotic therapy, *P. aeruginosa* acquires resistance through chromosomally-encoded mutations that lead to the constitutive overexpression of RND-type efflux pumps and AmpC cephalosporinase (Hocquet et al., 2007a; Cabot et al., 2011). 14 to 56% of failed therapy in patients under antipseudomonal therapy have been observed due to AmpC derepression (Lister et al., 2009). Moreover, the expression of the substrate-specific porin OprD, promoting the entry of carbapenems, (Trias and Nikaido, 1990a,b), is frequently downregulated by both transcriptional and translational mechanisms (Lister et al., 2009). That these mechanisms are often present simultaneously in *P. aeruginosa*, thereby conferring multiresistant phenotypes is of serious concern (Livermore, 2002; Poole, 2011). A single method detecting all aforementioned mechanisms would be a useful aid for selecting the most appropriate antibiotic in order to counteract antibiotic resistance emergence in this opportunistic pathogen.

Selected Reaction Monitoring (SRM) is a targeted Mass Spectrometry-based (MS) technique that has favorable performance characteristics compared with other MS techniques (Domon and Aebersold, 2010). It makes use of triple quadrupole mass spectrometers to selectively isolate precursor ions corresponding to the mass of the targeted peptides and to selectively monitor peptide-specific fragments. This particular mode of operation provides a high level of selectivity and sensitivity and a wide dynamic range of analysis even in complex samples (Picotti et al., 2009). Suitable sets of precursor and fragment ion masses for a given peptide, called SRM transitions, constitute definitive MS assays that identify a peptide and, by inference, its cognate protein in the proteome digests (Lange et al., 2008). An additional advantage of SRM is the high degree of analyte multiplexing. Here we finalized a scheduled SRM method to quantify antibiotic resistance mechanisms at the protein level. *P. aeruginosa*-specific peptides have been simultaneously investigated to normalize sample quantities by taking advantage of multiplexing capacities. We also developed a generic sample preparation and chromatographic separation method to be used prior to electrospray ionization (ESI) triple quadrupole mass spectrometric analysis. Our data show that a label-free SRM-based method supports the evaluation of antibiotic resistance mechanisms in both laboratory and clinical strains.

## Materials and methods

### Bacterial strain, antibacterial agents, and chemicals

Strains of the literature-based set were previously characterized (Table S1). Strains of the clinical-based set came from different hospitals (Table S2). All strains were stored as glycerol stocks at −80°C. Subcultures were performed on Mueller-Hinton agar medium (Becton Dickinson AG, Allschwil, Switzerland) at 37°C for 18 h in aerobic conditions. Strains were sub-cultured in 5 mL Lysogeny-Broth medium (Becton Dickinson AG) and grown during 5 h at 37°C with shaking. A 1 mL aliquot was centrifuged at 3500 g during 10 min and supernatant was discarded. Aliquots taken from the same culture were used immediately or frozen at −20°C until use. One aliquot was used for RT-qPCR analysis and three aliquots were used for SRM analysis. All of the samples were assayed in technical triplicates for both techniques unless specified for SRM (Table S3). Antimicrobial disk diffusion tests were performed according to the Clinical and Laboratory Standards Institute (Table S4).

### RT-qPCR experiment

#### Isolation of total RNA

Lysis of bacterial cells was performed with 1 mg/mL lysozyme in Tris/EDTA buffer (10 mM Tris-HCl, pH 8.0, containing 1 mM EDTA). Total RNA was isolated according to the instructions of the supplier using a RNeasy kit (Qiagen, Hilden, Germany). Residual DNA was eliminated by DNase treatment using 10U of RNase-free DNase (Roche, Basel, Switzerland). DNase was removed by repeating the column based purification and the RNA was eluted with 30 μL of RNase-free H_2_O. Purified RNA was quantified using a spectrophotometer (ND-8000 NanoDrop Technologies, Wilmington, DE) and stored at −80°C. A reaction mix containing 1 μg of RNA was incubated in the presence of 250 ng random hexamers (Promega, Madison, United States) and dNTPs (10 mM final concentration) in a total volume of 13 μL at 65°C for 5 min. After chilling the mix on ice, 4 μL of 5X first strand buffer, 2 μL of 100 mM dithiotreitol (Sigma-Aldrich-Fluka, Lyon, France) and 1 μL of Superscript III™ reverse transcriptase (Invitrogen, Carlsbad, CA) were added. Reactions were incubated for 5 min at 25°C and then at 50°C for 50 min. Reverse transcriptase was inactivated by incubation at 70°C for 15 min. 1μL of RNase H (Invitrogen) was added and the mix incubated at 37°C for 20 min to degrade RNA. The cDNAs obtained were purified using QIAquick PCR purification kit (Qiagen, Hilden, Germany) following suppliers instructions and stored at −20°C until use.

### RT-qPCR analysis

The primers for the PCR amplification of cDNA were retrieved from literature sources or designed using the primer3 program (Untergasser et al., 2012) (Table S5). An Mx3005P qPCR system (Agilent, Santa Clara, United States) was used for the quantification of cDNA. Triplicate PCR reactions were performed using the Brilliant II SYBR Green Q-PCR Master mix (Agilent). Five microliters of a 1 ng/μL cDNA concentration were used in a total volume of 15 μL. After a 10-min initial denaturation step, 40 cycles of 30 s at 95°C and 60 s at 60°C were performed. A melt curve was run at the end of the 40 cycles to test for the presence of a unique PCR reaction product. To check for residual contaminating genomic DNA, control reactions without reverse transcriptase were analyzed in the same way using the rpsL-F/R primers. The amount of signal in the controls was usually close to the non-template control.

To correct for differences in the amount of starting material, the ribosomal *rpsL* gene was selected as a housekeeping reference gene. Results were presented as ratios of gene expression between the target gene (target) and the reference gene (*rpsL*), which were obtained according to the following equation:

$$ratio=\frac{\text{E }target^{\Delta \text{CP}(\text{Control}-\text{Sample})}}{\text{E }reference^{\Delta \text{CP}(\text{Control}-\text{Sample})}}$$

(Pfaffl et al., 2002), where E is the real-time PCR efficiency for a given gene and ΔCP the crossing point (CP) difference (Δ) of the amplification curve with the threshold. PCR efficiencies were corrected according to LinRegPCR program (Ruijter et al., 2009) and the median of individual values was calculated for each gene.

### SRM experiment

#### Bacterial lysis and digestion

Frozen aliquots of bacterial pellets were thawed and introduced into 1.5 mL Eppendorf tubes, containing a mixture of glass beads and 150 μL of 50 mM NH_4_HCO_3_, 5 mM dithiotreitol (Sigma-Aldrich-Fluka), *pH* = 8.0. The tubes were placed on an ultrasound probe (Hielscher Ultrasonics GmBH, Teltow, Germany) and the samples were disrupted during 5 min. They were further alkylated with 12.5 mM iodoacetamide (Sigma-Aldrich-Fluka) during 5 min in the dark. Trypsin (10 μg) (Sigma-Aldrich-Fluka) was added to the samples and the digestion was performed on a heating block at 50°C during 15 min. The tryptic digestion was stopped by acidifying samples with 0.5 μL formic acid (Sigma-Aldrich-Fluka). The samples were desalted using Oasis HLB 3 cm^3^ (60 mg) reversed phase cartridges (Waters, Milford, MA). The cartridges were conditioned with 1 mL of methanol (Merck Millipore, Billerica, MA), then with 1 mL of water (LC-MS grade, Fisher Scientific, Strasbourg, France) containing 0.1% formic acid prior to loading of the tryptic digest. Cartridges were washed with 1 mL of water containing 0.1% formic acid and eluted with methanol/water (80:20, v/v) containing 0.1% formic acid. The samples were dried by vacuum centrifugation, suspended in 200 μL of water/acetonitrile (95/5, v/v) containing 0.5% of formic acid and directly analyzed or stored at −20°C until analysis.

### Liquid chromatography (LC) and mass spectrometry (MS) analysis

Identification-based analysis was performed on an Ultimate 3000 HPLC instrument (Dionex Corporation, Sunnyvale, CA) coupled to an hybrid triple quadripole/time of flight 5600 TripleTOF (AB Sciex, Foster City, CA) equipped with an ESI Turbo V ion source. LC separation was carried out on a XBridge BEH C18 column (150 mm × 2.1 mm, particle size 3.5 μm, porosity 130 Å) from Waters. Elution was performed at a flow rate of 300 μL/min with water (LC-MS grade, Fisher Scientific) containing 0.1% (v/v) formic acid as solvent A and acetonitrile (LC-MS grade, Fisher Scientific) containing 0.1% (v/v) formic acid as solvent B. An isocratic step at 5% solvent B during 3 min was followed by a 60-min linear gradient from 5 to 45% solvent B. MS analysis was carried out in positive ionization mode using an ion spray voltage of 5500 V. The nebulizer and the curtain gas flows were set at 50 psi using nitrogen. The Turbo V ion source was operated at 550°C with the auxiliary gas flow (nitrogen) set at 50 psi. For peptide identification, raw data were processed in ProteinPilot v4.0 (AB Sciex) and searched using Paragon Algorithm (Shilov et al., 2007) against SwissProt Pseudomonadales database (April 2013) using the following parameters. The search effort was Rapid Identification, trypsin was specified for digestion, iodoacetamide was specified for cysteine alkylation, and the instrument specified was 5600TripleTOF. A protein score >1.3 from software supplier (called Unused Prot Score) was used for scoring and selection of proteins. Peptides were filtered using a peptide confidence score >90.

Targeted analysis was performed in SRM mode on a Nexera HPLC instrument (Shimadzu, Kyoto, Japan) hyphenated to an hybrid triple quadripole/linear ion trap mass spectrometer 5500 QTRAP (AB Sciex) equipped with an ESI Turbo V ion source. Instrument control, data acquisition and processing were performed using Analyst 1.5.1 software. LC separation was carried out on an XBridge BEH C18 column (100 mm × 2.1 mm, particle size 3.5 μm, porosity 130 Å) from Waters. Elution was performed at a flow rate of 300 μL/min with water (LC-MS grade, Fisher Scientific) containing 0.1% (v/v) formic acid as solvent A and acetonitrile (LC-MS grade, Fisher Scientific) containing 0.1% (v/v) formic acid as solvent B. An isocratic step at 2% solvent B during 3 min was followed by a 22-min linear gradient from 2 to 35% solvent B. MS analysis was carried out in positive ionization mode using an ion spray voltage of 5500 V. The nebulizer and the curtain gas flows were set at 50 psi using nitrogen. The Turbo V ion source was operated at 550°C with the auxiliary gas flow (nitrogen) set at 50 psi.

### SRM assays construction

SRM assays were performed directly on digests from *P. aeruginosa* cell pellets with a high content of proteins of interest. Antibiotic-resistance proteins were digested *in silico* using Skyline software (Maclean et al., 2010). All possible fully tryptic, doubly and triply charged peptides that were between 7 and 20 amino acids in length were selected. Carbamidomethyl cysteines were considered. For each peptide all singly and doubly charged product ions from ion three to the last ion were selected. The three most intense transitions were selected based on library built with previous data dependent analyses acquired on a 5600 TripleTOF (AB Sciex). Collision energy and other settings were predicted according to Skyline equations for 5500 QTRAP instrument and not optimized further. Samples with a high content of proteins of interest were injected in SRM mode with several methods. Based on these results, retention times were adjusted and poorly responding peptides were discarded. Previous data-dependent analyses allowed us to select *P. aeruginosa*-specific peptide candidates for bacterial quantification. Specificity to *P. aeruginosa* was evaluated by BLAST analysis (Altschul et al., 1990). Non-specific peptides were discarded as well as peptides containing methionine or missed-cleavage sites. Digests of *P. aeruginosa* cell pellet were injected in SRM mode. Simultaneously, retention times were adjusted and poorly responding peptides were discarded while best responding peptides were selected as candidates for bacterial quantification and added in the final scheduled SRM method (Table S6).

### Data analysis

MultiQuantTM 2.1 software (AB Sciex), with the integration algorithm Summation for peak integration, was used for SRM data analysis. Peak integration parameters were set as follows with a Gaussian smooth width of 1.0 point, a summation window of 10 s, a recentering window of 20 s around the expected retention time and a 10% noise level for baseline.

### Normalization method

Vandesompele et al. has proposed an internal control gene-stability measure called M (Vandesompele et al., 2002). For every control gene they determined the pairwise variation with all other control genes as the standard deviation of the logarithmically transformed expression ratios and defined M as the average pairwise variation of a particular gene with all other control genes. We applied these equations (Vandesompele et al., 2002) for SRM assays. *M*-values were calculated for each *P. aeruginosa*-specific transition and averaged by peptide. A stepwise exclusion strategy was chosen until average *M*-values were below 0.7. Peptides and associated transitions that respected these rules were selected for the internal control protein-stability measure M (Table S7). To take into account the response factor, individual transition areas values were divided by the average transition area. Averages were based on samples with transition areas above 3000 arbitrary units (a.u.). Contrary to Vandesompele et al. a median was preferred to a geometric mean to reduce the effect of outliers. The normalization factor (NF) used for each sample was the median of these ratios.

### Quantotypic peptides selection

Quantotypic peptides are stoichiometric to the level of each protein (Worboys et al., 2014). Selection of quantotypic peptides were based on Pearson correlation. Correlation analysis was performed with Pearson's product moment correlation on log-normalized areas of transitions. A critical value of >0.8 was fixed to be a relevant transition as surrogate of protein level. For a given transition, when two correlation analyses from different peptides did not respect this rule, the transition was excluded from the median calculation (Table S8). Correlation were based on samples with transition areas above 2000 a.u..

### Protein quantification

The underlying assumption for protein quantification in bottom-up proteomics is that the level of the measured peptides is stoichiometric to the level of the protein. To take into account the response factor, individual transition area values were divided by the average transition area. Averages were based on samples with transition areas above 3000 a.u.. The protein abundance estimation was the median of these ratios.

### Protein ratio

For each sample, protein ratios were compared to a control sample using the following equation:

$$protein\;ratio=\left(\frac{\text{median }target}{\text{median }NF}\right)\text{ sample }.\;\left(\frac{\text{median }NF}{\text{median }target}\right)\text{ control}$$

where target corresponds to the protein of interest and NF to the normalization factor.

### OprD protein case

The OprD porin presents substantial polymorphism (Pirnay et al., 2002), seven representative protein sequences were retrieved from Uniprot database (Uniprot release 2013_03). Based on SRM results, four combinations of peptides (SRM profile) were observed and led to five types of protein sequences (Table S9). To take into account the response factor, individual transition area values were divided by the average transition area. Averages were based on samples with transition areas above 2000 a.u.. However, a different average was obtained for each SRM profile. Common peptides among SRM profiles allowed the calculation of a corrective factor (Table S9).

## Results

To assess the possibility of using SRM to detect antimicrobial resistance mechanisms in *P. aeruginosa*, we measured, simultaneously, protein production of the four major efflux pumps, the chromosomal AmpC β-lactamase and the porin OprD in different strains. The approach was compared to RT-qPCR which has been used previously (Dumas et al., 2006). Initially, the approach was assessed thanks to a literature-based set of strains for which resistance mechanisms were well characterized (Table S1). Subsequently, the approach was compared to a clinical-based set of strains for which resistance mechanisms were to be determined (Table S2).

### Evaluation of efflux system expression in the literature-based set

*P. aeruginosa* harbors 12 RND-type efflux pumps on its chromosome, however only the MexAB-OprM, MexCD-OprJ, MexEF-OprN, and MexXY pumps have been shown to be of clinical relevance (Poole et al., 1993, 1996; Köhler et al., 1997; Mine et al., 1999). Among the strain test set, isolates known in the literature to have a basal level expression of efflux system were used to assess the distribution of basal level expression. Strains with expression levels statistically above this distribution were considered as efflux pump overexpressers. Using this classification scheme for both strain sets, we found that, LC-ESI-SRM outperformed RT-qPCR in the literature-based set (Table 1). Classification of efflux system expression levels was 100% in agreement with literature data using SRM compared to 93% using RT-qPCR.

**Table 1:** **Classification of efflux systems status among *P. aeruginosa* strains in the literature-based set**.

**Efflux systems**	**Expected result**	**Strains classified according to**			
		**LC-ESI-SRM (protein)**		**RT-qPCR (mRNA)**	
		**Basal level**	**Over-produced**	**Basal level**	**Over-expressed**
MexAB-OprM^a^	Basal level (*n* = 17)	17	0	16	1
	Over-expressed (*n* = 12)	0	12	5	7
MexCD-OprJ^b^	Basal level (*n* = 29)	29	0	29	0
	Over-expressed (*n* = 0)	0	0	0	0
MexEF-OprN^c^	Basal level (*n* = 22)	22	0	21	1
	Over-expressed (*n* = 7)	0	7	0	7
MexXY^d^	Basal level (*n* = 14)	14	0	13	1
	Over-expressed (*n* = 15)	0	15	0	15

*A two-sample two-sided t-test was performed on strains with basal level efflux system expression. For each subunit of efflux system, 95% confidence intervals were deduced*.

a *Overexpression was significant when MexA/mexA and MexB/mexB measurements were above the confidence interval*.

b *Overexpression was significant when mexC and mexD measurements were above the confidence interval. Basal level strains did not have detectable MexC or MexD protein production*.

c *Overexpression was significant when mexE and mexF measurements were above the confidence interval. Strains expressing efflux systems at a basal level did not have detectable MexE or MexF protein production except for 4 strains allowing to estimate basal level production*.

d *Overexpression was significant when mexX and mexY measurements were above the confidence interval*.

**Figure 1:**
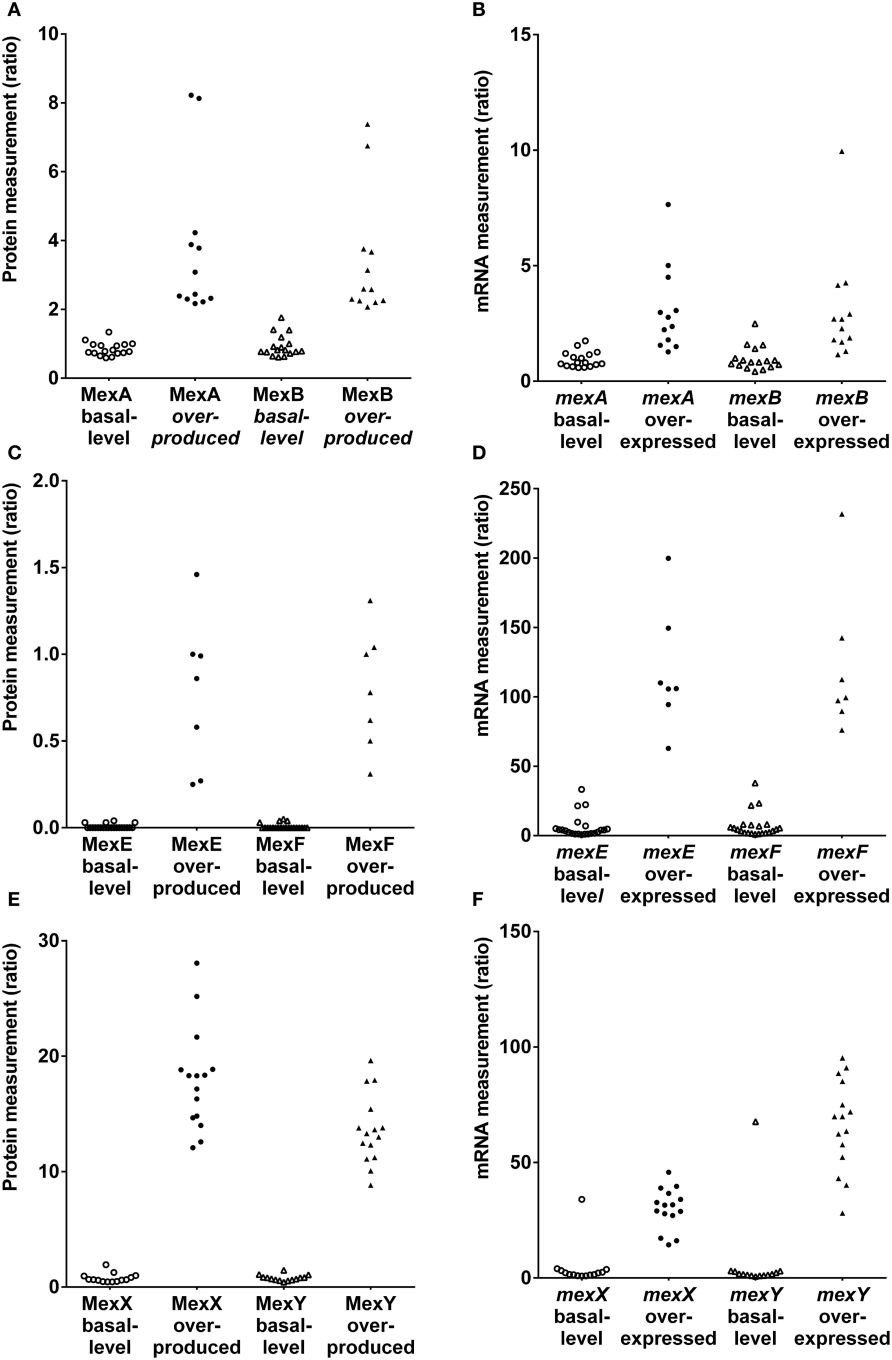
**Protien and mRNA measurements of efflux systems among *P. aeruginosa* strains in the literature-based set**. **(A,C,E)**. Protein measurements of MexAB-OprM, MexEF-OprN and MexXY(-OprM), respectively. **(B,D,F)**. mRNA measurements *mexA, mexB, mexE, mexF and mexX, mexY efflux* genes, respectively.

Discrepancies were mainly observed for the MexAB-OprM efflux system (Figure 1; Table S10). Five strains (1217, 1562, 2085, 2151, and 2172) expected to be overexpressed for this tripartite system were not correctly classified using RT-qPCR. However, strains producing basal level expression were correctly assigned by RT-qPCR, except for strain 2112S. Based on results, a two-fold change or less was sufficient to consider this efflux system as overproduced. The correlation coefficient between MexA and MexB subunits production was 0.98 for SRM and only 0.93 for RT-qPCR analysis of *mexA* and *mexB* gene expression (Figures S1–S2). Nevertheless, we had to pinpoint a correlation coefficient about 0.84 between MexA protein and *mexA* RNA analyses (Figure S3).

In contrast to the constitutively expressed MexAB-OprM efflux system, the MexCD-OprJ pump is expressed at clinically relevant levels only when mutations arise in the cognate repressor gene *nfxB* (Hosaka et al., 1995). Unfortunately, none of the strains in the literature-based set was an *nfxB* mutant. Both techniques classified correctly each strain (Figure 1; Table S11). Notably, protein production was undetectable in these strains. This observation was in agreement with the fact that the *mexCD-oprJ* operon is not constitutively expressed (Hosaka et al., 1995).

The *mexEF-oprN* operon is positively regulated by the LysR-type activator protein MexT and is overexpressed in *nfxC* mutants (Köhler et al., 1997, 1999). Both techniques classified correctly each strain except 615R which was classified as overexpresser by RT-qPCR (Figure 1; Table S12). Interestingly, a low level of protein production was detected in this strain and undetected in most of the other strains. Conversely, RT-qPCR seemed to be too sensitive and did not reflect a real overexpression. A threshold may be applied to compensate for RT-qPCR sensitivity. Nevertheless, both techniques were precise based on correlation coefficients (Figures S4, S5, *R*^2^ = 0.98).

MexXY-OprM is involved in the intrinsic and acquired resistance of *P. aeruginosa* to aminoglycoside antibiotics (Aires et al., 1999). The *mexXY* operon does not contain a gene coding for an outer membrane protein but is able to recruit OprM of the MexAB-OprM efflux pump to form a functional tripartite efflux system (Mine et al., 1999). Because mutational events in the regulators may lead to the constitutive overproduction of MexXY in clinical strains (Morita et al., 2012), the MexXY efflux system was measured under non inducing conditions. Both RT-qPCR and SRM techniques were able to correctly classify each strain (Figure 1; Table S13) except for strain 2112S with RT-qPCR. Referring to MexXY basal-level producer strains, the highest MexY protein quantity was observed in strain 2112S. These results were in agreement with immunodetection of MexY reported by Vogne et al. in this strain (Vogne et al., 2004). RT-qPCR detected similar *mexXY* transcript quantities in these paired strains 2112S and 2112R. Post-transcriptional events may occur in these paired isolates preventing to know clinical relevance in strain 2112S thanks to RT-qPCR. Both SRM and RT-qPCR yielded excellent correlations between either MexX and MexY proteins or *mexX* and *mexY* mRNAs (respectively, *R*^2^ = 0.99 and 0.94, Figures S6, S7) whereas there was no correlation between OprM and either MexX or MexY (respectively, *R*^2^ = 0.24 and 0.28, Figures S8, S9) supporting independent expression of *mexXY* and *oprM*.

### Evaluation of efflux system expression in the clinical-based set

Overexpression of *mexAB-oprM* has been detected in *nalB*-, *nalC*-, and *nalD*-type multidrug resistant mutants, selected both *in vivo* and *in vitro* (Lister et al., 2009). In 6 out of 15 clinical strains, overproduction was detected by LC-ESI-SRM (Table 2; Table S10). Correlation between amounts of MexA and MexB protein corroborated this result (*R*^2^ = 0.98 and slope = 0.976, Figure S10). In contrast, RT-qPCR failed to classify 5 out of 6 strains as *mexAB* overexpressers (Table 2; Table S10). The only strain (Pa-006) classified as *mexAB* over-expresser by RT-qPCR had the highest-fold change ratios for *mexA* and *mexB* which were statistically significant. Lack of precision for other strains is reflected by the low correlation coefficient between *mexA* and *mexB* mRNA expression (*R*^2^ = 0.725 and slope = 1.797, Figure S11).

**Table 2:** **Comparison of LC-ESI-SRM vs. RT-qPCR results to classify efflux systems status among *P. aeruginosa* clinical isolates**.

**Efflux systems**	**Strains classified according to**		
	**LC-ESI-SRM (protein)**	**RT-qPCR (mRNA)**	
		**Basal level**	**Over-expressed**
MexAB-OprM	Basal level (9)	9	0
	Over-produced (6)	5	1
MexCD-OprJ	Basal level (12)	12	0
	Over-produced (3)	0	3
MexEF-OprN	Basal level (15)	15	0
	Over-produced (0)	0	0
MexXY	Basal level (5)	5	0
	Over-produced (10)	2	8

*The same cutoffs used in the literature-based set were applied to the clinical-based set. Strains expressing efflux systems at a basal level did not have detectable MexC or MexD protein production. Overproduction was considered significant when MexC and MexD were detectable in the clinical-based set*.

Given the crucial role of the complement in the host defense against *P. aeruginosa* infections, *nfxB* mutants emerged rarely and mainly in patients with compromised complement levels such as isolates from cystic fibrosis patients with chronic respiratory infections (Jalal et al., 2000; Mueller-Ortiz et al., 2004; Martinez-Ramos et al., 2014). Indeed, mutation of *nfxB* has been reported to be involved in the early adaptation to the chronic setting (Rau et al., 2010). In 3 out of 15 strains, overproduction of MexCD-OprJ was detected by SRM and RT-qPCR. Both techniques seemed to classify correctly each strain (Table 2; Table S11).

Neither RT-qPCR nor SRM identified MexEF-OprN overproducers among the 15 clinical isolates, supporting the idea that they are rarely found in clinical settings (Hocquet et al., 2007b). Protein expression levels of MexEF-OprN were undetectable using SRM. Although *mexE*, *mexF* or *oprN* overexpression was detected in some of the isolates by RT-qPCR, none of them fulfilled the criteria to be considered as a *mexEF-oprN* overexpresser.

Both techniques were able to classify correctly MexXY overexpression except for two strains (Pa-007, Pa-010). In these two strains only *mexY* was overexpressed but not *mexX*. Based on SRM measurements, the MexXY efflux system was overproduced in 10 out of 15 clinical strains.

### AmpC-mediated resistance

The inducible cephalosporinase AmpC (Livermore, 1995) was measured under non-inducing conditions. AmpC derepression enabled to predict resistance to antipseudomonal penicillins or cephalosporins thanks to a threshold level. AmpC expression levels were compared to antibiogram phenotypes (Table S4). SRM and RT-qPCR predicted antibiotic resistance most readily when production or expression levels were 3 times and 15 times higher than those of PAO1, respectively. Only 3 discrepancies out of 44 strains analyzed were observed between the two techniques (Table S14). For two strains (1237 and Pa-001), RT-qPCR detected *ampC*-derepression but not SRM, while strain PT1155 was identified as AmpC derepressed by SRM but not by RT-qPCR. The antibiogram suggested that AmpC was not derepressed in strain 1237 and in strain Pa-001 an extended-spectrum β-lactamase has been identified preventing assumptions on phenotype (Table S5). In the previously characterized strain PT1155, AmpC was derepressed by SRM but showed an increase close to the threshold by RT-qPCR. Overall, label-free SRM-based AmpC detection predictions showed a better consistency with the antibiogram data than RT-qPCR. The label-free SRM approach showed agreement with disk diffusion assays of 88, 95, and 90% for piperacillin/tazobactam, ceftazidime and cefepime, respectively (Table 3). The RT-qPCR approach showed agreement with disk diffusion assays of 88, 93, and 90% for piperacillin/tazobactam, ceftazidime and cefepime, respectively (Table 4).

**Table 3:** **Protein-based prediction of antipseudomonal antibiotic resistance and agreement with disk diffusion**.

**Protein-based prediction**	**Drugs**	**Agreement with disk diffusion (number of strains; %)**	**Minor error**	**Major error**	**Very major error**
Highly reduced	imipenem	35	5	0	1
OprD production^a^		85.4%	12.2%	0.0%	2.4%
	meropenem	37	3	1	0
		90.2%	7.3%	2.4%	0.0%
Derepressed	PIP/TAZO^c^	36	4	1	0
AmpC^b^		87.8%	9.8%	2.4%	0.0%
	ceftazidime	39	1	1	0
		95.1%	2.4%	2.4%	0.0%
	cefepime	37	1	2	1
		90.2%	2.4%	4.9%	2.4%

a *Highly reduced OprD protein production, defined as 20 times less produced than PAO1 level measured by LC-ESI-SRM, predicted carbapenem resistance, otherwise susceptible*.

b *Derepressed AmpC protein, defined as 3 times more produced than PAO1 level without induction measured by LC-ESI-SRM, predicted piperacillin/tazobactam, ceftafidime and cefepime resistance, otherwise susceptible*.

c *PIP/TAZO corresponds to piperacillin/tazobactam combination. The error rate determination is as follow: Minor error – reference result is R or S and device result is I; reference result is I and device result is R or S. Major error – reference result is S and device result is R. Very major error – reference result is R and device result is S*.

**Table 4:** **RNA-based prediction of antipseudomonal antibiotic resistance and agreement with disk diffusion**.

**RNA-based prediction**	**Drugs**	**Agreement with disk diffusion (number of strains; %)**	**Minor error**	**Major error**	**Very major error**
Highly reduced	imipenem	28	5	0	5
*oprD* expression^a^		73.7%	13.2%	0.0%	13.2%
	meropenem	30	3	1	4
		78.9%	7.9%	2.6%	10.5%
Derepressed	PIP/TAZO^c^	36	4	1	0
*ampC*^b^		87.8%	9.8%	2.4%	0.0%
	ceftazidime	38	1	1	1
		92.7%	2.4%	2.4%	2.4%
	cefepime	37	1	2	1
		90.2%	2.4%	4.9%	2.4%

a *Highly reduced oprD mRNA expression, defined as 5 times less expressed than PAO1 level measured by RT-qPCR, predicted carbapenem resistance, otherwise susceptible*.

b *Derepressed ampC mRNA, defined as 15 times more produced than PAO1 level without induction measured by RT-qPCR, predicted piperacillin/tazobactam, ceftafidime and cefepime resistance, otherwise susceptible*.

c *PIP/TAZO corresponds to piperacillin/tazobactam combination. The error rate determination is as follow: Minor error – reference result is R or S and device result is I; reference result is I and device result is R or S. Major error – reference result is S and device result is R. Very major error – reference result is R and device result is S*.

### OprD-mediated resistance

OprD loss enabled prediction of carbapenem resistance based on the established threshold level of 20 times and 5 times lower expression levels than those of PAO1 for SRM and RT-qPCR, respectively. Levels were assigned according to meropenem and imipenem susceptibilities. Notably 4 discrepancies out of 41 were observed between the two techniques (Table S15). For these strains (504, 1237, 2112S, 2112R), SRM detected OprD deficiency whereas RT-qPCR did not, despite a consensus design of primers. Antibiotic susceptibility testing confirmed SRM results as the four strains were resistant to meropenem and imipenem (Table S4). These results may suggest non-transcriptional events preventing OprD insertion in these strains (misfolding, enzymatic degradation, no outer membrane insertion by the Bam complex…). Overall, label-free SRM-based OprD detection was the best predictor for carbapenem resistance. Agreements with standard procedures were 85 and 90% for imipenem and meropenem, respectively, using SRM-based OprD quantification (Table 3) while agreements with standard procedures were 74 and 79% for imipenem and meropenem, respectively, using RT-qPCR-based *oprD* quantification (Table 4).

## Discussion

### Strengths and weaknesses of SRM vs. RT-qPCR

#### 1/Weak under- and over-expression

Weak under- and over-expression, as illustrated by MexAB-OprM efflux system, were more precisely assessed using protein quantification rather than mRNA quantification. Various reasons can explain this observation. The first relates to the accuracy of the antimicrobial resistance mechanism measure. For each antimicrobial resistance mechanism (protein or gene), multiple independent assays per replicate called transitions were performed for SRM whereas only one assay per replicate was performed for RT-qPCR as is typically the case. In targeted proteomics, it is critical that peptides are not only proteotypic but also accurately represent the level of the protein (quantotypic). As recently proposed by Worboys et al. (2014), selection of quantotypic peptides was performed according to a Pearson correlation between pairs of transitions across samples. Both mRNA and peptides could be unstable but in the case of SRM, the more stable peptides were selected and averaged to reduce the variance. A second reason relates to the accuracy of the normalization step. The normalization of sample quantity is a very critical step. For RT-qPCR, internal control genes called housekeeping genes assume that role. Vandesompele et al. clearly showed that geometric averaging of multiple and well-selected internal control genes improve the accuracy of normalization compared to single control normalization (Vandesompele et al., 2002). It should be possible to measure various housekeeping genes but at the cost of a higher workload and price. For RT-qPCR, a single control gene procedure was therefore performed while multiple peptides were assayed and selected effortlessly for the internal control protein-stability measure. A third reason is the multiplexing capacity. Multiplexing of RT-qPCR is possible but is limited by fluorescence labels to six genes. Conversely, SRM present the advantage of having a higher multiplexing capacity.

#### 2/Protein functionality and antibiotic resistance prediction

A restriction inherent to gene expression analysis is that presence of a transcript does not necessarily mean that it encodes a functional protein. Additional regulatory events can affect protein production. At least four cases of additional regulation for OprD and one case for MexXY-OprM were suspected with RT-qPCR. In contrast, protein detection has the ability to delineate the functional units of a cell and is more likely to predict the phenotypic differences such as antibiotic susceptibility. Overproduction of efflux systems contributed to reduce susceptibilities to cephalosporins and carbapenems but these were not the main resistance mechanisms (Wang et al., 2010; Cabot et al., 2011). Although antibiotic resistance implies various mechanisms, predictions were only based on one protein. Prediction of anti-pseudomonal cephalosporin resistance was based on AmpC detection, while prediction of carbapenem resistance was based on OprD detection. Both proteins measured by SRM gave accurate antimicrobial susceptibility prediction in accordance with phenotypic methods.

#### 3/Sensitivity to genetic polymorphism

Design of primers for RT-qPCR experiments can be difficult because of genetic sequence polymorphism which could be the case for *oprD* gene expression (Pirnay et al., 2002). Low transcripts could artificially appear due to primer non-hybridization because of polymorphism. For SRM, knowledge of protein sequence is mandatory but various specific peptides could be detected. For example, three to four peptides per protein were independently detected, giving SRM more autonomy to detect OprD genetic polymorphism.

### Strengths and weaknesses of label-free SRM vs. others mass spectrometry-based techniques

Several mass spectrometry-based approaches have been used to perform quantitative proteomics and have been reviewed recently, for instance, to study carbapenem resistance in *Acinetobacter baumannii* (Tiwari and Tiwari, 2014). Here, we deliberately chose a bottom-up approach and more precisely a peptide-centric strategy because separation, solubilization, ionization, and fragmentation of peptides was easier and more effective than for the parent proteins. A targeted approach was rationally preferred to a shotgun approach. Reproducibility is lacking for shotgun approaches (Tabb et al., 2010) and identification is limited to database exhaustiveness (Duncan et al., 2010). SRM is the dedicated targeted approach and was therefore selected. Two challenges for effective relative quantification have been overcome: normalization of sample quantity and estimation of protein abundance. Classically, addition of stable isotopes through peptides, proteins or bacteria is used to improve the precision. However, labeled peptides or protein would be inadequate to normalize sample quantities through the experiment since bacteria quantities and bacterial lysis would not be exactly the same between samples. Moreover, labeling of bacteria is really cumbersome and expensive. Addition of stable isotopes was therefore discarded because of convenience and price. Tagging of samples was also possible but techniques were limited to 10 samples (McAlister et al., 2012) and sample preparation also became cumbersome and expensive. Consequently, a label-free approach was deliberately chosen. Quantification was based on the principle claiming a direct relationship between signal measured and amount of analytes present in a sample. For label-free quantification, all the issues that may impair quantification accuracy and precision accumulate. It was decided to keep sample preparation as short as possible, to use a very stable HPLC system (microflow chromatography) and to process all samples in the same experiment in order to prevent reproducibility issues.

In conclusion, the SRM method shows results that are equivalent or even better than those provided by RT-qPCR and the method is generic, rapid, multiplexed and applicable beyond genetic polymorphisms. It was able to detect antibiotic resistance mechanisms including the AmpC cephalosporinase, the OprD porin and the four major efflux pumps in both laboratory and clinical strains. A generic sample preparation without detergent enables to quantify integral membrane proteins. SRM-based predictions of antibiotic resistance proved to be accurate for cephalosporin and carbapenem susceptibilities. As multidrug resistance of *P. aeruginosa* involves several resistance mechanisms, this multiplexed method should allow a more educated choice of antimicrobial treatment.

## Author contributions

Yannick Charretier grew the bacteria, performed the sample preparation, developed the SRM method and performed the analysis of LC-ESI-SRM and RT-qPCR. Tiphaine Cecchini provided help and support with MS experiments and SRM method development. Chloé Bardet performed *P. aeruginosa*-specific candidate selection. Abdessalam Cherkaoui performed the disk diffusion assays. Abdessalam Cherkaoui, Thilo Köhler, Catherine Llanes, Pierre Bogaerts and Jacques Schrenzel provided input in the microbiological field and gave some strains for the study. Jacques Schrenzel and Sonia Chatellier supervised the research. Yannick Charretier interpreted the results. Yannick Charretier drafted the work and Thilo Köhler, Tiphaine Cecchini, Chloé Bardet, Catherine Llanes, Pierre Bogaerts, Jacques Schrenzel and Jean-Philippe Charrier edited the paper.

### Conflict of interest statement

Yannick Charretier, Tiphaine Cecchini, Chloé Bardet, Sonia Chatellier and Jean-Philippe Charrier are or were employed by BioMérieux and they have filed patents assigned to BioMérieux. Jacques Schrenzel reports research grants from BioMérieux. The authors declare that the research was conducted in the absence of any commercial or financial relationships that could be construed as a potential conflict of interest.

## Abbreviations

ESI: Electrospray Ionization
LC: Liquid Chromatography
MS: Mass Spectrometry
RT-qPCR: Reverse Transcription quantitative PCR
SRM: Selected Reaction Monitoring
