# Supplementary material for: Label-free SRM-based relative quantification of antibiotic resistance mechanisms in Pseudomonas aeruginosa clinical isolates
Source: Front Microbiol. 2015 Feb 10;6:81. doi: 10.3389/fmicb.2015.00081 (PMC4322712; doi:10.3389/fmicb.2015.00081)
Supplement: Supplementary file 1 [file Table1.PDF]

# *Supplementary Material*

# Label-free SRM-based relative quantification of antibiotic resistance mechanisms in *Pseudomonas aeruginosa* clinical isolates

Y. Charretier[1*], T.Köhler[2], T. Cecchini[3, 4], C. Bardet[5, 6], A. Cherkaoui[7], C. Llanes[8], P. Bogaerts[9], S. Chatellier[10], J-P. Charrier[4], J. Schrenzel[1, 7]

[1]Genomic Research Laboratory, Service of Infectious Diseases, Geneva University Hospitals, Geneva, Switzerland
[2]Department of Microbiology and Molecular Medicine, University of Geneva, Geneva, Switzerland
[3]Institute for Analytical Sciences, Joint Research Unit 5280 CNRS/Lyon 1 University, Villeurbanne, France
[4]Technology Research Department, bioMérieux SA, Marcy l'Etoile, France
[5]UMR1092 INSERM, Limoges University, France
[6]MD3, bioMérieux SA, Marcy l'Etoile, France
[7]Clinical Microbiology Laboratory, Service of Infectious Diseases, Geneva University Hospitals, Geneva, Switzerland
[8]Laboratoire de bactériologie, EA4266, Université de Franche-Comté, Besançon, France
[9]Laboratoire de bactériologie, Université Catholique de Louvain, CHU de Mont-Godinne, Yvoir, Belgique
[10]Microbiology Unit, bioMérieux SA, La Balme Les Grottes, France

*** Correspondence:** Yannick Charretier, Genomic Research Laboratory, Service of Infectious Diseases, Geneva University Hospitals, Rue Gabrielle-Perret-Gentil 4, CH-1211 GENEVA 14, Switzerland.
e-mail: yannick.charretier@genomic.ch

# 1. Supplementary Figures and Tables

## 1.1. Supplementary Tables

### Supplementary Table 1. Literature-based selected strains.

| Sample name | Mechanism of interest | Regulation | Genotype | Parent isolate | Additional features undescribed | Reference |
|---|---|---|---|---|---|---|
| 40-1 | MexEF-OprN | overexpressed | *nfxC* | blood culture isolate | | (Llanes et al., 2011) |
| 93-1 | MexEF-OprN ; AmpC | overexpressed | *nfxC* | blood culture isolate | | (Llanes et al., 2011) |
| 1113 | MexAB-OprM ; MexXY | overexpressed | *nalD, agrZ* | tracheal aspirate isolate | | (Llanes et al., 2004) |
| 1217 | MexAB-OprM ; MexXY | overexpressed | *nalD, agrZ* | urinary isolate | OprD | (Llanes et al., 2004) |
| 1237 | MexAB-OprM ; MexXY | overexpressed | *nalB, agrZ* | urinary isolate | OprD | (Llanes et al., 2004) |
| 1250 | MexAB-OprM ; MexXY | overexpressed | *nalC, agrZ* | blood catheter isolate | | (Llanes et al., 2004) |
| 1562 | MexAB-OprM ; MexXY | overexpressed | *nalD, agrZ* | urinary isolate | OprD | (Llanes et al., 2004) |
| 1727 | MexAB-OprM ; MexXY | overexpressed | *nalB, nalC, agrW* | tracheal aspirate isolate | | (Llanes et al., 2004) |
| 1738 | MexAB-OprM ; MexXY | overexpressed | *nalC, agrW* | tracheal aspirate isolate | | (Llanes et al., 2004) |
| 2085 | MexAB-OprM ; MexXY | overexpressed | *nalB, agrZ* | surgical wound isolate | | (Llanes et al., 2004) |
| 2151 | MexAB-OprM ; MexXY | overexpressed | *nalB, agrZ* | blood catheter isolate | | (Llanes et al., 2004) |
| 2172 | MexAB-OprM ; MexXY | overexpressed | *nalC, agrZ* | urinary isolate | OprD | (Llanes et al., 2004) |
| 3936 | MexEF-OprN | overexpressed | *nfxC* | respiratory isolate | | (Llanes et al., 2011) |
| PT1105 | OprD | decreased | *czcS* | PAO1 | | (Dumas et al., 2006) |
| PT1155 | AmpC | derepressed | *ampD* | PAO1 | | (Dumas et al., 2006) |
| PT1196 | MexXY | overexpressed | *agrZ* | PAO1 | | Kholer, unpublished data |
| 14 | MexAB-OprM | basal level | *wild type* | clinical isolate | MexXY | (Ziha-Zarifi et al., 1999) |
| 12 | MexAB-OprM | overexpressed | *nalB* | 14 | MexXY | (Ziha-Zarifi et al., 1999) |
| 615S | MexXY | basal level | *wild type* | cystic fibrosis isolate | MexEF-OprN | (Vogne et al., 2004) |
| 615R | MexXY | overexpressed | *agrZ* | 615S | | (Vogne et al., 2004) |
| 2112S | MexXY | basal level | *wild type* | cystic fibrosis isolate | AmpC, OprD | (Vogne et al., 2004) |
| 2112R | MexXY | overexpressed | *agrZ* | 2112S | AmpC, OprD | (Vogne et al., 2004) |
| PT149 | MexEF-OprN | overexpressed | *nfxC* | PAO1 | | (Dumas et al., 2006) |
| PT364 | OprD | loss | lack | PAO1 | | (Dumas et al., 2006) |
| PAO1 | wild type | basal level | *wild type* | reference strain | | |
| PT629 | MexAB-OprM | overexpressed | *nalB* | PAO1 | | (Dumas et al., 2006) |
| 302S | MexEF-OprN | basal level | *wild type* | blood culture isolate | | (Dumas et al., 2006) |
| 302R9N | MexEF-OprN | overexpressed | *nfxC* | 302S | | (Dumas et al., 2006) |
| 302R11N | MexEF-OprN | overexpressed | *nfxC* | 302S | | (Dumas et al., 2006) |

**Supplementary Table 2. Clinical-based selected strains.**

| Isolates | Date of isolation | Hospital center | Source of isolation |
|---|---|---|---|
| 113 | 2013-05-10 | HUG | Artificial joint |
| 124.1 | 2013-05-09 | HUG | Sputum |
| 124.2 | 2013-05-09 | HUG | Sputum |
| 138 | 2013-05-11 | HUG | Urine from urinary catheter |
| 188 | 2013-05-08 | HUG | Deep wound from sacrum |
| 504 | 2013-05-07 | HUG | Tracheotomy pus |
| Pa-001 | 2010-09-16 | CHU ST-PIERRE - BRUXELLES | Bronchial aspirate |
| Pa-002 | 2010-09-17 | CHU ST-PIERRE - BRUXELLES | Tracheobronchic aspirate |
| Pa-004 | 2010-03-13 | ST-JOSEPH-ARLON | Urine |
| Pa-005 | 2010-01-05 | UZ ANTWERPEN | Endotracheal aspirate |
| Pa-006 | 2011-06-27 | AZ JAN YPERMAN - IEPER | Wound/decubitus eschar |
| Pa-007 | 2009-03-17 | UZ GENT | Urine |
| Pa-008 | 2009-03-17 | UZ GENT | Pus/wound |
| Pa-009 | 2009-03-16 | AZ GROENINGE - KORTRIJK | Pus/wound |
| Pa-010 | 2011-08-19 | UZ GENT | Blood |

**Supplementary Table 3. Number of technical replicates for SRM analysis**

| Sample name | Number of technical replicates for SRM analysis |
|---|---|
| 40-1 | 3 |
| 93-1 | 3 |
| 1113 | 2 |
| 1217 | 2 |
| 1237 | 2 |
| 1250 | 2 |
| 1562 | 2 |
| 1727 | 2 |
| 1738 | 2 |
| 2085 | 2 |
| 2151 | 2 |
| 2172 | 2 |
| 3936 | 3 |
| PT1105 | 2 |
| PT1155 | 2 |
| PT1196 | 2 |
| 14 | 3 |
| 12 | 3 |
| 615S | 3 |
| 615R | 3 |
| 2112S | 3 |
| 2112R | 3 |
| PT149 | 3 |
| PT364 | 3 |
| PAO1 | 2 |
| PT629 | 3 |
| 302S | 3 |
| 302R9N | 2 |
| 302R11N | 3 |
| 113 | 3 |
| 124.1 | 3 |
| 124.2 | 2 |
| 138 | 2 |
| 188 | 2 |
| 504 | 2 |
| Pa-001 | 2 |
| Pa-002 | 2 |
| Pa-004 | 2 |
| Pa-005 | 2 |
| Pa-006 | 1 |
| Pa-007 | 2 |
| Pa-008 | 1 |
| Pa-009 | 1 |
| Pa-010 | 2 |

**Supplementary Table 4. Antimicrobial disk diffusion tests.**

| Sample | Zone diameter (in mm) of antibiotics[a] tested | | | | | | | | | | | | | |
|---|---|---|---|---|---|---|---|---|---|---|---|---|---|---|
| | PIP | TZP | CAZ | FEP | IPM | MEM | ATM | AMK | GEN | TOB | NOR | CIP | SXT | PB |
| 40-1 | 32 | 33 | 28 | 29 | 21 | 29 | 30 | 20 | 16 | 22 | 14 | 16 | 6 | 18 |
| 93-1 | 16 | 16 | 6 | 17 | 17 | 18 | 17 | 23 | 20 | 26 | 17 | 20 | 6 | 17 |
| 113 | 27 | 30 | 29 | 26 | 33 | 35 | 26 | 22 | 18 | 23 | 27 | 30 | 6 | 17 |
| 124.1 | 22 | 26 | 27 | 24 | 18 | 35 | 25 | 19 | 17 | 23 | 25 | 28 | 6 | 18 |
| 124.2 | 22 | 23 | 22 | 22 | 21 | 31 | 21 | 20 | 16 | 22 | 14 | 16 | 6 | 18 |
| 138 | 23 | 29 | 27 | 28 | 29 | 31 | 22 | 24 | 20 | 24 | 31 | 32 | 6 | 16 |
| 188 | 24 | 28 | 27 | 29 | 21 | 26 | 26 | 22 | 18 | 23 | 27 | 28 | 6 | 16 |
| 504 | 29 | 33 | 30 | 27 | 11 | 14 | 28 | 20 | 17 | 22 | 29 | 30 | 11 | 17 |
| 1113 | 24 | 25 | 25 | 21 | 24 | 24 | 19 | 17 | 14 | 20 | 25 | 27 | 6 | 16 |
| 1217 | 24 | 25 | 25 | 18 | 6 | 6 | 17 | 6 | 6 | 12 | 6 | 6 | 6 | 19 |
| 1237 | 22 | 23 | 26 | 21 | 9 | 6 | 21 | 14 | 6 | 6 | 6 | 6 | 6 | 17 |
| 1250 | 23 | 26 | 24 | 21 | 24 | 21 | 17 | 17 | 15 | 22 | 23 | 25 | 6 | 17 |
| 1562 | 25 | 27 | 27 | 22 | 6 | 6 | 23 | 6 | 6 | 6 | 6 | 6 | 6 | 16 |
| 1727 | 22 | 23 | 22 | 22 | 28 | 29 | 16 | 11 | 6 | 19 | 22 | 22 | 6 | 18 |
| 1738 | 25 | 27 | 28 | 26 | 9 | 24 | 23 | 16 | 13 | 22 | 21 | 23 | 6 | 17 |
| 2085 | 22 | 23 | 22 | 20 | 26 | 24 | 18 | 14 | 6 | 19 | 6 | 6 | 6 | 17 |
| 2151 | 24 | 26 | 24 | 20 | 18 | 16 | 22 | 18 | 14 | 21 | 6 | 6 | 6 | 16 |
| 2172 | 20 | 22 | 23 | 20 | 9 | 6 | 18 | 17 | 11 | 20 | 21 | 24 | 6 | 17 |
| 3936 | 33 | 34 | 29 | 32 | 19 | 29 | 32 | 27 | 21 | 26 | 23 | 25 | 6 | 16 |
| Pa-001 | 16 | 18 | 6 | 6 | 6 | 6 | 6 | 6 | 6 | 6 | 6 | 6 | 6 | 18 |
| Pa-002 | 6 | 6 | 17 | 6 | 10 | 6 | 7 | 6 | 6 | 6 | 6 | 6 | 6 | 18 |
| Pa-004 | 27 | 28 | 22 | 13 | 23 | 20 | 14 | 16 | 6 | 6 | 6 | 6 | 6 | 16 |
| Pa-005 | 6 | 16 | 6 | 6 | 12 | 6 | 6 | 6 | 6 | 6 | 6 | 6 | 6 | 17 |
| Pa-006 | 23 | 24 | 23 | 20 | 12 | 6 | 16 | 17 | 6 | 6 | 6 | 6 | 6 | 17 |
| Pa-007 | 18 | 20 | 13 | 21 | 22 | 25 | 12 | 6 | 15 | 24 | 6 | 6 | 6 | 18 |
| Pa-008 | 6 | 9 | 14 | 6 | 11 | 10 | 14 | 19 | 15 | 24 | 6 | 6 | 6 | 18 |
| Pa-009 | 6 | 7 | 8 | 14 | 8 | 8 | 12 | 18 | 6 | 6 | 15 | 14 | 6 | 17 |
| Pa-010 | 6 | 16 | 6 | 6 | 6 | 6 | 6 | 16 | 6 | 6 | 6 | 6 | 6 | 16 |
| PT1105 | 31 | 34 | 29 | 31 | 18 | 23 | 30 | 23 | 18 | 25 | 32 | 36 | 6 | 16 |
| PT1155 | 24 | 26 | 22 | 26 | 27 | 27 | 26 | 21 | 18 | 23 | 29 | 32 | 6 | 16 |
| PT1196 | 17 | 23 | 27 | 21 | 28 | 32 | 28 | 17 | 14 | 22 | 25 | 27 | 6 | 17 |
| 14 | 31 | 31 | 27 | 23 | 26 | 27 | 26 | 18 | 13 | 20 | 27 | 28 | 6 | 16 |
| 12 | 24 | 24 | 23 | 21 | 29 | 23 | 14 | 19 | 15 | 21 | 27 | 29 | 6 | 17 |
| 615S | 40 | 40 | 31 | 35 | 30 | 38 | 38 | 24 | 19 | 26 | 14 | 18 | 6 | 17 |
| 615R | 38 | 38 | 28 | 26 | 31 | 38 | 37 | 14 | 10 | 19 | 29 | 27 | 8 | 17 |
| 2112S | 16 | 18 | 6 | 6 | 13 | 6 | 6 | 21 | 20 | 26 | 26 | 27 | 6 | 18 |
| 2112R | 14 | 16 | 6 | 6 | 9 | 11 | 6 | 13 | 6 | 18 | 16 | 17 | 6 | 18 |
| PT149 | 32 | 34 | 27 | 31 | 21 | 29 | 32 | 25 | 20 | 26 | 16 | 18 | 6 | 16 |
| PT364 | 27 | 33 | 28 | 29 | 12 | 18 | 29 | 23 | 19 | 26 | 31 | 31 | 6 | 16 |
| PAO1 | 29 | 33 | 27 | 29 | 26 | 31 | 29 | 22 | 17 | 24 | 30 | 33 | 6 | 16 |
| PT629 | 24 | 26 | 24 | 24 | 26 | 25 | 19 | 22 | 17 | 23 | 25 | 26 | 6 | 16 |
| 302S | 29 | 34 | 27 | 29 | 27 | 36 | 28 | 21 | 15 | 21 | 31 | 32 | 8 | 16 |
| 302R9N | 29 | 34 | 27 | 28 | 16 | 33 | 26 | 21 | 15 | 21 | 16 | 20 | 6 | 16 |
| 302R11N | 31 | 35 | 29 | 30 | 19 | 33 | 28 | 20 | 17 | 22 | 24 | 27 | 6 | 16 |

[a]Antibiotics tested: PIP, piperacillin; TZP, piperacillin/tazobactam; CAZ, ceftazidime; FEP, cefepime; IPM, imipenem; MEM, Meropenem; ATM, aztreonam; AMK, amikacin; GEN, gentamicin; TOB, tobramycin; NOR, norfloxacin; CIP, ciprofloxacin; SXT, trimethoprim-

sulfamethoxazole; PB, polymyxin B

**Supplementary Table 5. Primers used in this study**

| Gene name | Primer Sequence (5'-3') | Reference |
|---|---|---|
| *ampC* | CGGCTCGGTGAGCAAGACCTTC | (Dumas et al., 2006) |
| *ampC* | AGTCGCGGATCTGTGCCTGGTC | (Dumas et al., 2006) |
| *mexA* | CGACCAGGCCGTGAGCAAGCAGC | (Dumas et al., 2006) |
| *mexA* | GGAGACCTTCGCCGCGTTGTCGC | (Dumas et al., 2006) |
| *mexB* | GGGTGACCAAGGCGGTGAAGAAC | this study |
| *mexB* | TACTGCGAGCCGAACACCTGGAA | this study |
| *mexC* | ATCCGGCACCGCTGAAGGCTGCG | (Dumas et al., 2006) |
| *mexC* | CGGATCGAGCTGCTGGATGCGCG | (Dumas et al., 2006) |
| *mexD* | TTCGCCTGGGTGGTGGCCCTGTT | this study |
| *mexD* | TCTCGGCGGTGCCGTTGGAGTTG | this study |
| *mexE* | CGACAACGCCAAGGGCGAGTTCACC | (Dumas et al., 2006) |
| *mexE* | CCAGGACCAGCACGAACTTCTTGC | (Dumas et al., 2006) |
| *mexF* | AGGCCTCGCCCGACCTGACCATG | this study |
| *mexF* | CTCGCGGATGGCGTTGACCACGT | this study |
| *mexX* | TGAAGGCGGCCCTGGACATCAGC | (Dumas et al., 2006) |
| *mexX* | GATCTGCTCGACGCGGGTCAGCG | (Dumas et al., 2006) |
| *mexY* | TGGTCAACGTCAGCGCCAGCTAT | this study |
| *mexY* | TCGACGATCTTCAGGCGGTTCTG | this study |
| *oprJ* | GTTCCGGGCCTGAATGCCGCTGC | (Dumas et al., 2006) |
| *oprJ* | TCGCGGCTGACCAGGGTCTGACG | (Dumas et al., 2006) |
| *oprM* | GATCCCCGACTACCAGCGCCCCG | (Dumas et al., 2006) |
| *oprM* | ATGCGGTACTGCGCCCGGAAGGC | (Dumas et al., 2006) |
| *oprN* | CAACCGGGAGTGACCGAGGACCG | (Dumas et al., 2006) |
| *oprN* | TGCTCAGGGCAATCTTCTCGCGC | (Dumas et al., 2006) |
| *rpsL* | GCAAGCGCATGGTCGACAAGA | (Dumas et al., 2006) |
| *rpsL* | CGCTGTGCTCTTGCAGGTTGTGA | (Dumas et al., 2006) |
| *oprD* | ACCAACCTCGAAGCCAAGTA | (Llanes et al., 2013) |
| *oprD* | ACAGGATCGACAGCGGATAG | (Llanes et al., 2013) |

## Supplementary Table 6. SRM method

| Protein name | Accession number | Gene name | Proteotypic peptide sequence | Q1 - Precursor ion (m/z) | Q3 - Fragment ion (m/z) | Target retention time (min) | Collision energy (Volts) | Conserved versus excluded transitions (literature-based analysis) |
|---|---|---|---|---|---|---|---|---|
| AmpC | P24735 | *ampC* | ALVDAAVQPVMK | 621.35 | 958.503 | 13.2 | 31.2 | excluded |
| AmpC | P24735 | *ampC* | ALVDAAVQPVMK | 621.35 | 772.439 | 13.2 | 31.2 | excluded |
| AmpC | P24735 | *ampC* | ALVDAAVQPVMK | 621.35 | 474.274 | 13.2 | 31.2 | excluded |
| AmpC | P24735 | *ampC* | DLGLVILANR | 542.33 | 685.436 | 18.6 | 28.4 | conserved |
| AmpC | P24735 | *ampC* | DLGLVILANR | 542.33 | 586.367 | 18.6 | 28.4 | conserved |
| AmpC | P24735 | *ampC* | DLGLVILANR | 542.33 | 286.14 | 18.6 | 28.4 | conserved |
| AmpC | P24735 | *ampC* | FVDANLHPER | 599.304 | 951.464 | 9.9 | 30.4 | conserved |
| AmpC | P24735 | *ampC* | FVDANLHPER | 599.304 | 538.273 | 9.9 | 30.4 | conserved |
| AmpC | P24735 | *ampC* | FVDANLHPER | 599.304 | 401.214 | 9.9 | 30.4 | conserved |
| AmpC | P24735 | *ampC* | LDRPWAQALDATHR | 550.622 | 783.411 | 14.2 | 27.5 | excluded |
| AmpC | P24735 | *ampC* | LDRPWAQALDATHR | 550.622 | 599.29 | 14.2 | 27.5 | excluded |
| AmpC | P24735 | *ampC* | LDRPWAQALDATHR | 550.622 | 484.263 | 14.2 | 27.5 | excluded |
| AmpC | P24735 | *ampC* | LPAPQALEGQR | 590.328 | 898.474 | 10 | 30.1 | conserved |
| AmpC | P24735 | *ampC* | LPAPQALEGQR | 590.328 | 673.363 | 10 | 30.1 | conserved |
| AmpC | P24735 | *ampC* | LPAPQALEGQR | 590.328 | 449.741 | 10 | 30.1 | conserved |
| AmpC | P24735 | *ampC* | LQAGNSTPMALQPHR | 540.947 | 949.504 | 11.1 | 27 | excluded |
| AmpC | P24735 | *ampC* | LQAGNSTPMALQPHR | 540.947 | 409.231 | 11.1 | 27 | excluded |
| AmpC | P24735 | *ampC* | LQAGNSTPMALQPHR | 540.947 | 690.346 | 11.1 | 27 | excluded |
| AmpC | P24735 | *ampC* | SLGQPFER | 467.243 | 733.363 | 10.6 | 25.7 | conserved |
| AmpC | P24735 | *ampC* | SLGQPFER | 467.243 | 548.283 | 10.6 | 25.7 | conserved |
| AmpC | P24735 | *ampC* | SLGQPFER | 467.243 | 451.23 | 10.6 | 25.7 | conserved |
| AmpC | P24735 | *ampC* | TGSTNGFGAYVAFVPGR | 850.923 | 908.499 | 18 | 39.5 | conserved |
| AmpC | P24735 | *ampC* | TGSTNGFGAYVAFVPGR | 850.923 | 646.367 | 18 | 39.5 | conserved |
| AmpC | P24735 | *ampC* | TGSTNGFGAYVAFVPGR | 850.923 | 329.193 | 18 | 39.5 | conserved |
| AmpC | P24735 | *ampC* | TSAADLLR | 423.738 | 658.388 | 11 | 24.1 | conserved |
| AmpC | P24735 | *ampC* | TSAADLLR | 423.738 | 587.351 | 11 | 24.1 | conserved |
| AmpC | P24735 | *ampC* | TSAADLLR | 423.738 | 401.287 | 11 | 24.1 | conserved |
| AmpC | P24735 | *ampC* | VGPGPLDAEGYGVK | 679.851 | 838.394 | 11.6 | 33.3 | conserved |
| AmpC | P24735 | *ampC* | VGPGPLDAEGYGVK | 679.851 | 630.317 | 11.6 | 33.3 | conserved |
| AmpC | P24735 | *ampC* | VGPGPLDAEGYGVK | 679.851 | 601.806 | 11.6 | 33.3 | conserved |
| CARB-4 | Q51355 | *carB4* | FQQVEQDAK | 546.77 | 817.405 | 6.3 | 28.5 | excluded |
| CARB-4 | Q51355 | *carB4* | FQQVEQDAK | 546.77 | 689.346 | 6.3 | 28.5 | excluded |
| CARB-4 | Q51355 | *carB4* | FQQVEQDAK | 546.77 | 590.278 | 6.3 | 28.5 | excluded |
| CARB-4 | Q51355 | *carB4* | SVLPEGWNIADR | 678.849 | 831.411 | 15.4 | 33.3 | excluded |
| CARB-4 | Q51355 | *carB4* | SVLPEGWNIADR | 678.849 | 529.257 | 15.4 | 33.3 | excluded |
| CARB-4 | Q51355 | *carB4* | SVLPEGWNIADR | 678.849 | 300.192 | 15.4 | 33.3 | excluded |
| Multidrug resistance protein MexA | P52477 | *mexA* | AILAPQQGVTR | 577.338 | 969.548 | 10.6 | 29.6 | conserved |
| Multidrug resistance protein MexA | P52477 | *mexA* | AILAPQQGVTR | 577.338 | 856.464 | 10.6 | 29.6 | conserved |
| Multidrug resistance protein MexA | P52477 | *mexA* | AILAPQQGVTR | 577.338 | 785.426 | 10.6 | 29.6 | conserved |
| Multidrug resistance protein MexA | P52477 | *mexA* | GQATALVVNAQNK | 657.362 | 772.431 | 9.7 | 32.5 | conserved |
| Multidrug resistance protein MexA | P52477 | *mexA* | GQATALVVNAQNK | 657.362 | 673.363 | 9.7 | 32.5 | conserved |
| Multidrug resistance protein MexA | P52477 | *mexA* | GQATALVVNAQNK | 657.362 | 574.294 | 9.7 | 32.5 | conserved |
| Multidrug resistance protein MexA | P52477 | *mexA* | IITEGLQFVQPGVEVK | 878.996 | 628.366 | 17.3 | 40.5 | conserved |
| Multidrug resistance protein MexA | P52477 | *mexA* | IITEGLQFVQPGVEVK | 586.333 | 756.425 | 17.3 | 29.5 | conserved |
| Multidrug resistance protein MexA | P52477 | *mexA* | IITEGLQFVQPGVEVK | 586.333 | 628.366 | 17.3 | 29.5 | conserved |
| Multidrug resistance protein MexA | P52477 | *mexA* | LEDGSQYPLEGR | 682.328 | 862.442 | 10.6 | 33.4 | conserved |
| Multidrug resistance protein MexA | P52477 | *mexA* | LEDGSQYPLEGR | 682.328 | 734.383 | 10.6 | 33.4 | conserved |
| Multidrug resistance protein MexA | P52477 | *mexA* | LEDGSQYPLEGR | 682.328 | 571.32 | 10.6 | 33.4 | conserved |

Table S6 contiuation

| Protein name | Accession number | Gene name | Proteotypic peptide sequence | Q1 - Precursor ion (m/z) | Q3 - Fragment ion (m/z) | Target retention time (min) | Collision energy (Volts) | Conserved versus excluded transitions (literature-based analysis) |
|---|---|---|---|---|---|---|---|---|
| Multidrug resistance protein MexB | P52002 | *mexB* | EDLSNYIVSNIQDPLSR | 654.997 | 715.373 | 19.5 | 33.2 | conserved |
| Multidrug resistance protein MexB | P52002 | *mexB* | EDLSNYIVSNIQDPLSR | 654.997 | 587.315 | 19.5 | 33.2 | conserved |
| Multidrug resistance protein MexB | P52002 | *mexB* | EDLSNYIVSNIQDPLSR | 654.997 | 472.288 | 19.5 | 33.2 | conserved |
| Multidrug resistance protein MexB | P52002 | *mexB* | GQQLNATIIGK | 571.83 | 957.573 | 12 | 29.4 | conserved |
| Multidrug resistance protein MexB | P52002 | *mexB* | GQQLNATIIGK | 571.83 | 716.43 | 12 | 29.4 | conserved |
| Multidrug resistance protein MexB | P52002 | *mexB* | GQQLNATIIGK | 571.83 | 317.218 | 12 | 29.4 | conserved |
| Multidrug resistance protein MexB | P52002 | *mexB* | LATGANALDTAK | 573.312 | 961.495 | 9.3 | 29.5 | conserved |
| Multidrug resistance protein MexB | P52002 | *mexB* | LATGANALDTAK | 573.312 | 860.447 | 9.3 | 29.5 | conserved |
| Multidrug resistance protein MexB | P52002 | *mexB* | LATGANALDTAK | 573.312 | 481.251 | 9.3 | 29.5 | conserved |
| Multidrug resistance protein MexB | P52002 | *mexB* | LQLATPLLPQEVQR | 803.47 | 756.4 | 16.1 | 37.8 | conserved |
| Multidrug resistance protein MexB | P52002 | *mexB* | LQLATPLLPQEVQR | 803.47 | 355.234 | 16.1 | 37.8 | conserved |
| Multidrug resistance protein MexB | P52002 | *mexB* | LQLATPLLPQEVQR | 803.47 | 850.54 | 16.1 | 37.8 | conserved |
| Multidrug resistance protein MexB | P52002 | *mexB* | NAILIVEFAK | 559.334 | 932.582 | 18.1 | 29 | excluded |
| Multidrug resistance protein MexB | P52002 | *mexB* | NAILIVEFAK | 559.334 | 819.497 | 18.1 | 29 | excluded |
| Multidrug resistance protein MexB | P52002 | *mexB* | NAILIVEFAK | 559.334 | 706.413 | 18.1 | 29 | excluded |
| Multidrug resistance protein MexB | P52002 | *mexB* | TQVVVDSMR | 517.769 | 805.424 | 10 | 27.5 | excluded |
| Multidrug resistance protein MexB | P52002 | *mexB* | TQVVVDSMR | 517.769 | 706.355 | 10 | 27.5 | excluded |
| Multidrug resistance protein MexB | P52002 | *mexB* | TQVVVDSMR | 517.769 | 607.287 | 10 | 27.5 | excluded |
| MexC | G3XD25 | *mexC* | AGDLLFQIDPAPLK | 749.419 | 640.366 | 20 | 35.8 | conserved |
| MexC | G3XD25 | *mexC* | AGDLLFQIDPAPLK | 749.419 | 525.34 | 20 | 35.8 | conserved |
| MexC | G3XD25 | *mexC* | AGDLLFQIDPAPLK | 749.419 | 357.25 | 20 | 35.8 | conserved |
| MexC | G3XD25 | *mexC* | AVLFEAQAR | 502.78 | 834.447 | 11.6 | 27 | conserved |
| MexC | G3XD25 | *mexC* | AVLFEAQAR | 502.78 | 721.363 | 11.6 | 27 | conserved |
| MexC | G3XD25 | *mexC* | AVLFEAQAR | 502.78 | 574.294 | 11.6 | 27 | conserved |
| MexC | G3XD25 | *mexC* | IQAVSQQDFDTATADLR | 939.963 | 747.4 | 14.1 | 42.7 | conserved |
| MexC | G3XD25 | *mexC* | IQAVSQQDFDTATADLR | 939.963 | 313.187 | 14.1 | 42.7 | conserved |
| MexC | G3XD25 | *mexC* | IQAVSQQDFDTATADLR | 939.963 | 755.405 | 14.1 | 42.7 | conserved |
| MexC | G3XD25 | *mexC* | LNLGYASVTAPISGR | 759.917 | 958.532 | 14.9 | 36.2 | conserved |
| MexC | G3XD25 | *mexC* | LNLGYASVTAPISGR | 759.917 | 887.495 | 14.9 | 36.2 | conserved |
| MexC | G3XD25 | *mexC* | LNLGYASVTAPISGR | 759.917 | 701.394 | 14.9 | 36.2 | conserved |
| MexC | G3XD25 | *mexC* | SAQADLETAR | 531.265 | 903.453 | 7.2 | 28 | conserved |
| MexC | G3XD25 | *mexC* | SAQADLETAR | 531.265 | 775.394 | 7.2 | 28 | conserved |
| MexC | G3XD25 | *mexC* | SAQADLETAR | 531.265 | 476.246 | 7.2 | 28 | conserved |
| MexC | G3XD25 | *mexC* | TPQGIDNQAILVPQR | 825.452 | 725.467 | 13.4 | 38.6 | conserved |
| MexC | G3XD25 | *mexC* | TPQGIDNQAILVPQR | 825.452 | 612.383 | 13.4 | 38.6 | conserved |
| MexC | G3XD25 | *mexC* | TPQGIDNQAILVPQR | 825.452 | 400.23 | 13.4 | 38.6 | conserved |
| MexC | G3XD25 | *mexC* | VIVGGLAAVQPGVK | 654.406 | 996.584 | 14.2 | 32.4 | conserved |
| MexC | G3XD25 | *mexC* | VIVGGLAAVQPGVK | 654.406 | 769.457 | 14.2 | 32.4 | conserved |
| MexC | G3XD25 | *mexC* | VIVGGLAAVQPGVK | 654.406 | 400.255 | 14.2 | 32.4 | conserved |
| MexD | Q9HVI9 | *mexD* | GTLDDPQEFGQVVLR | 837.428 | 947.531 | 16.4 | 39 | conserved |
| MexD | Q9HVI9 | *mexD* | GTLDDPQEFGQVVLR | 837.428 | 818.488 | 16.4 | 39 | conserved |
| MexD | Q9HVI9 | *mexD* | GTLDDPQEFGQVVLR | 837.428 | 586.825 | 16.4 | 39 | conserved |
| MexD | Q9HVI9 | *mexD* | LVGFGLSIDDVSNAIR | 838.454 | 889.437 | 21.8 | 39 | conserved |
| MexD | Q9HVI9 | *mexD* | LVGFGLSIDDVSNAIR | 838.454 | 774.41 | 21.8 | 39 | conserved |
| MexD | Q9HVI9 | *mexD* | LVGFGLSIDDVSNAIR | 838.454 | 659.383 | 21.8 | 39 | conserved |

| Table S6 contiuation | | | | | | | | |
|---|---|---|---|---|---|---|---|---|
| Protein name | Accession number | Gene name | Proteotypic peptide sequence | Q1 - Precursor ion (m/z) | Q3 - Fragment ion (m/z) | Target retention time (min) | Collision energy (Volts) | Conserved versus excluded transitions (literature-based analysis) |
| MexD | Q9HVI9 | *mexD* | NAILIVEFAK | 559.334 | 932.582 | 18.1 | 29 | excluded |
| MexD | Q9HVI9 | *mexD* | NAILIVEFAK | 559.334 | 819.497 | 18.1 | 29 | excluded |
| MexD | Q9HVI9 | *mexD* | NAILIVEFAK | 559.334 | 706.413 | 18.1 | 29 | excluded |
| MexD | Q9HVI9 | *mexD* | VGLITIIGLSAK | 592.884 | 915.587 | 20.9 | 30.2 | excluded |
| MexD | Q9HVI9 | *mexD* | VGLITIIGLSAK | 592.884 | 802.503 | 20.9 | 30.2 | excluded |
| MexD | Q9HVI9 | *mexD* | VGLITIIGLSAK | 592.884 | 305.182 | 20.9 | 30.2 | excluded |
| MexD | Q9HVI9 | *mexD* | VVIQAEQGNR | 557.304 | 915.464 | 7.1 | 28.9 | conserved |
| MexD | Q9HVI9 | *mexD* | VVIQAEQGNR | 557.304 | 802.38 | 7.1 | 28.9 | conserved |
| MexD | Q9HVI9 | *mexD* | VVIQAEQGNR | 557.304 | 674.322 | 7.1 | 28.9 | conserved |
| MexE | Q9I0Y9 | *mexE* | AAVAATQAQLDAAR | 678.865 | 973.506 | 10.3 | 33.3 | conserved |
| MexE | Q9I0Y9 | *mexE* | AAVAATQAQLDAAR | 678.865 | 872.458 | 10.3 | 33.3 | conserved |
| MexE | Q9I0Y9 | *mexE* | AAVAATQAQLDAAR | 678.865 | 744.4 | 10.3 | 33.3 | conserved |
| MexE | Q9I0Y9 | *mexE* | ASNAISAELADAR | 644.828 | 945.5 | 13.7 | 32.1 | conserved |
| MexE | Q9I0Y9 | *mexE* | ASNAISAELADAR | 644.828 | 832.416 | 13.7 | 32.1 | conserved |
| MexE | Q9I0Y9 | *mexE* | ASNAISAELADAR | 644.828 | 344.156 | 13.7 | 32.1 | conserved |
| MexE | Q9I0Y9 | *mexE* | DEAVGTDLGK | 502.748 | 689.383 | 7.9 | 27 | conserved |
| MexE | Q9I0Y9 | *mexE* | DEAVGTDLGK | 502.748 | 590.314 | 7.9 | 27 | conserved |
| MexE | Q9I0Y9 | *mexE* | DEAVGTDLGK | 502.748 | 316.114 | 7.9 | 27 | conserved |
| MexE | Q9I0Y9 | *mexE* | FVLVLDGDNK | 560.306 | 873.468 | 16.2 | 29 | conserved |
| MexE | Q9I0Y9 | *mexE* | FVLVLDGDNK | 560.306 | 760.384 | 16.2 | 29 | conserved |
| MexE | Q9I0Y9 | *mexE* | FVLVLDGDNK | 560.306 | 661.315 | 16.2 | 29 | conserved |
| MexE | Q9I0Y9 | *mexE* | LDFLDNQVNPR | 665.841 | 842.411 | 13.6 | 32.8 | conserved |
| MexE | Q9I0Y9 | *mexE* | LDFLDNQVNPR | 665.841 | 386.215 | 13.6 | 32.8 | conserved |
| MexE | Q9I0Y9 | *mexE* | LDFLDNQVNPR | 665.841 | 376.187 | 13.6 | 32.8 | conserved |
| MexE | Q9I0Y9 | *mexE* | LEAPESVELRPR | 465.925 | 541.357 | 10.9 | 22.9 | conserved |
| MexE | Q9I0Y9 | *mexE* | LEAPESVELRPR | 465.925 | 641.841 | 10.9 | 22.9 | conserved |
| MexE | Q9I0Y9 | *mexE* | LEAPESVELRPR | 465.925 | 577.32 | 10.9 | 22.9 | conserved |
| MexE | Q9I0Y9 | *mexE* | LEAQLQQAR | 528.793 | 814.453 | 7.6 | 27.9 | conserved |
| MexE | Q9I0Y9 | *mexE* | LEAQLQQAR | 528.793 | 743.416 | 7.6 | 27.9 | conserved |
| MexE | Q9I0Y9 | *mexE* | LEAQLQQAR | 528.793 | 615.357 | 7.6 | 27.9 | conserved |
| MexE | Q9I0Y9 | *mexE* | VYAYFDADER | 624.78 | 986.421 | 12.2 | 31.3 | conserved |
| MexE | Q9I0Y9 | *mexE* | VYAYFDADER | 624.78 | 915.384 | 12.2 | 31.3 | conserved |
| MexE | Q9I0Y9 | *mexE* | VYAYFDADER | 624.78 | 752.321 | 12.2 | 31.3 | conserved |
| MexF | Q9I0Y8 | *mexF* | ASHGYVGTVNR | 580.794 | 865.453 | 6.4 | 29.8 | conserved |
| MexF | Q9I0Y8 | *mexF* | ASHGYVGTVNR | 580.794 | 808.431 | 6.4 | 29.8 | conserved |
| MexF | Q9I0Y8 | *mexF* | ASHGYVGTVNR | 580.794 | 501.759 | 6.4 | 29.8 | conserved |
| MexF | Q9I0Y8 | *mexF* | GNQGYEELFK | 592.783 | 885.435 | 12.9 | 30.2 | conserved |
| MexF | Q9I0Y8 | *mexF* | GNQGYEELFK | 592.783 | 828.414 | 12.9 | 30.2 | conserved |
| MexF | Q9I0Y8 | *mexF* | GNQGYEELFK | 592.783 | 665.35 | 12.9 | 30.2 | conserved |
| MexF | Q9I0Y8 | *mexF* | LEPEQIGQLK | 577.824 | 912.515 | 11.1 | 29.7 | conserved |
| MexF | Q9I0Y8 | *mexF* | LEPEQIGQLK | 577.824 | 445.277 | 11.1 | 29.7 | conserved |
| MexF | Q9I0Y8 | *mexF* | LEPEQIGQLK | 577.824 | 456.761 | 11.1 | 29.7 | conserved |
| MexF | Q9I0Y8 | *mexF* | LVTEEEFENIIIR | 802.93 | 904.525 | 18.3 | 37.8 | conserved |
| MexF | Q9I0Y8 | *mexF* | LVTEEEFENIIIR | 802.93 | 401.287 | 18.3 | 37.8 | conserved |
| MexF | Q9I0Y8 | *mexF* | LVTEEEFENIIIR | 802.93 | 696.854 | 18.3 | 37.8 | conserved |
| MexF | Q9I0Y8 | *mexF* | NAILIVEFAK | 559.334 | 932.582 | 18.1 | 29 | excluded |
| MexF | Q9I0Y8 | *mexF* | NAILIVEFAK | 559.334 | 819.497 | 18.1 | 29 | excluded |
| MexF | Q9I0Y8 | *mexF* | NAILIVEFAK | 559.334 | 706.413 | 18.1 | 29 | excluded |
| MexF | Q9I0Y8 | *mexF* | NLTATDVVNAIR | 643.857 | 958.532 | 16.5 | 32 | conserved |
| MexF | Q9I0Y8 | *mexF* | NLTATDVVNAIR | 643.857 | 887.495 | 16.5 | 32 | conserved |
| MexF | Q9I0Y8 | *mexF* | NLTATDVVNAIR | 643.857 | 572.352 | 16.5 | 32 | conserved |
| MexF | Q9I0Y8 | *mexF* | NNLGEMVPLASFIK | 766.911 | 874.54 | 22 | 36.5 | excluded |
| MexF | Q9I0Y8 | *mexF* | NNLGEMVPLASFIK | 766.911 | 775.471 | 22 | 36.5 | excluded |
| MexF | Q9I0Y8 | *mexF* | NNLGEMVPLASFIK | 766.911 | 342.177 | 22 | 36.5 | excluded |
| MexF | Q9I0Y8 | *mexF* | VAAVLEACR | 494.766 | 818.419 | 8.7 | 26.7 | conserved |
| MexF | Q9I0Y8 | *mexF* | VAAVLEACR | 494.766 | 648.313 | 8.7 | 26.7 | conserved |
| MexF | Q9I0Y8 | *mexF* | VAAVLEACR | 494.766 | 535.229 | 8.7 | 26.7 | conserved |
| MexX | G3XD21 | *mexX* | AVNPQAITVPR | 583.338 | 881.52 | 12 | 29.9 | conserved |
| MexX | G3XD21 | *mexX* | AVNPQAITVPR | 583.338 | 656.409 | 12 | 29.9 | conserved |
| MexX | G3XD21 | *mexX* | AVNPQAITVPR | 583.338 | 585.372 | 12 | 29.9 | conserved |

Table S6 contiuation

| Protein name | Accession number | Gene name | Proteotypic peptide sequence | Q1 - Precursor ion (m/z) | Q3 - Fragment ion (m/z) | Target retention time (min) | Collision energy (Volts) | Conserved versus excluded transitions (literature-based analysis) |
|---|---|---|---|---|---|---|---|---|
| MexX | G3XD21 | *mexX* | EYTEAQTDAR | 592.265 | 891.417 | 5.5 | 30.2 | excluded |
| MexX | G3XD21 | *mexX* | EYTEAQTDAR | 592.265 | 790.369 | 5.5 | 30.2 | excluded |
| MexX | G3XD21 | *mexX* | EYTEAQTDAR | 592.265 | 661.326 | 5.5 | 30.2 | excluded |
| MexX | G3XD21 | *mexX* | LGYATVTAPIDGR | 667.359 | 929.505 | 12.9 | 32.9 | conserved |
| MexX | G3XD21 | *mexX* | LGYATVTAPIDGR | 667.359 | 729.389 | 12.9 | 32.9 | conserved |
| MexX | G3XD21 | *mexX* | LGYATVTAPIDGR | 667.359 | 557.304 | 12.9 | 32.9 | conserved |
| MexX | G3XD21 | *mexX* | LYEEGQDVR | 554.767 | 832.38 | 8.8 | 28.8 | conserved |
| MexX | G3XD21 | *mexX* | LYEEGQDVR | 554.767 | 703.337 | 8.8 | 28.8 | conserved |
| MexX | G3XD21 | *mexX* | LYEEGQDVR | 554.767 | 574.294 | 8.8 | 28.8 | conserved |
| MexY | G3XCW2 | *mexY* | AAWTLGPPQLTR | 655.864 | 982.568 | 16.6 | 32.5 | excluded |
| MexY | G3XCW2 | *mexY* | AAWTLGPPQLTR | 655.864 | 768.436 | 16.6 | 32.5 | excluded |
| MexY | G3XCW2 | *mexY* | AAWTLGPPQLTR | 655.864 | 711.415 | 16.6 | 32.5 | excluded |
| MexY | G3XCW2 | *mexY* | EASQHVGAIVER | 648.339 | 880.5 | 8.5 | 32.2 | conserved |
| MexY | G3XCW2 | *mexY* | EASQHVGAIVER | 648.339 | 743.441 | 8.5 | 32.2 | conserved |
| MexY | G3XCW2 | *mexY* | EASQHVGAIVER | 648.339 | 644.373 | 8.5 | 32.2 | conserved |
| MexY | G3XCW2 | *mexY* | MAPGSNAVATAK | 559.287 | 915.489 | 6.9 | 29 | excluded |
| MexY | G3XCW2 | *mexY* | MAPGSNAVATAK | 559.287 | 818.437 | 6.9 | 29 | excluded |
| MexY | G3XCW2 | *mexY* | MAPGSNAVATAK | 559.287 | 458.248 | 6.9 | 29 | excluded |
| MexY | G3XCW2 | *mexY* | VGLITIIGLSAK | 592.884 | 915.587 | 20.9 | 30.2 | excluded |
| MexY | G3XCW2 | *mexY* | VGLITIIGLSAK | 592.884 | 802.503 | 20.9 | 30.2 | excluded |
| MexY | G3XCW2 | *mexY* | VGLITIIGLSAK | 592.884 | 305.182 | 20.9 | 30.2 | excluded |
| MexY | G3XCW2 | *mexY* | VVEEAVTAIIER | 664.875 | 872.52 | 15.1 | 32.8 | conserved |
| MexY | G3XCW2 | *mexY* | VVEEAVTAIIER | 664.875 | 801.483 | 15.1 | 32.8 | conserved |
| MexY | G3XCW2 | *mexY* | VVEEAVTAIIER | 664.875 | 702.414 | 15.1 | 32.8 | conserved |
| OprD | Q6QR54 | *oprD* | ANADEGEGDQNEFR | 776.319 | 865.38 | 7.3 | 36.8 | conserved |
| OprD | Q6QR54 | *oprD* | ANADEGEGDQNEFR | 776.319 | 693.331 | 7.3 | 36.8 | conserved |
| OprD | Q6QR54 | *oprD* | ANADEGEGDQNEFR | 776.319 | 501.194 | 7.3 | 36.8 | conserved |
| OprD | Q02IC0 | *oprD* | ANADQAEGDQNEFR | 782.835 | 994.422 | 7.2 | 37 | conserved |
| OprD | Q02IC0 | *oprD* | ANADQAEGDQNEFR | 782.835 | 865.38 | 7.2 | 37 | conserved |
| OprD | Q02IC0 | *oprD* | ANADQAEGDQNEFR | 782.835 | 700.29 | 7.2 | 37 | conserved |
| OprD | P32722 | *oprD* | ANADQGEGDQNEFR | 775.827 | 994.422 | 6.7 | 36.8 | conserved |
| OprD | P32722 | *oprD* | ANADQGEGDQNEFR | 775.827 | 865.38 | 6.7 | 36.8 | conserved |
| OprD | P32722 | *oprD* | ANADQGEGDQNEFR | 775.827 | 693.331 | 6.7 | 36.8 | conserved |
| OprD | P32722 | *oprD* | GFIEDSSLDLLLR | 739.398 | 916.546 | 20 | 35.5 | conserved |
| OprD | P32722 | *oprD* | GFIEDSSLDLLLR | 739.398 | 829.514 | 20 | 35.5 | conserved |
| OprD | P32722 | *oprD* | GFIEDSSLDLLLR | 739.398 | 447.224 | 20 | 35.5 | conserved |
| OprD | K1BSA2 | *oprD* | GFIEDSSLNLLLR | 738.906 | 915.562 | 19.6 | 35.5 | conserved |
| OprD | K1BSA2 | *oprD* | GFIEDSSLNLLLR | 738.906 | 828.53 | 19.6 | 35.5 | conserved |
| OprD | K1BSA2 | *oprD* | GFIEDSSLNLLLR | 738.906 | 628.414 | 19.6 | 35.5 | conserved |
| OprD | P32722 | *oprD* | HHETNLEAK | 539.767 | 941.469 | 4.8 | 28.3 | excluded |
| OprD | P32722 | *oprD* | HHETNLEAK | 539.767 | 471.238 | 4.8 | 28.3 | excluded |
| OprD | P32722 | *oprD* | HHETNLEAK | 539.767 | 619.258 | 4.8 | 28.3 | excluded |
| OprD | Q02IC0 | *oprD* | SGTGNLPVMNDGTPR | 758.365 | 986.472 | 11.2 | 36.2 | excluded |
| OprD | Q02IC0 | *oprD* | SGTGNLPVMNDGTPR | 758.365 | 430.241 | 11.2 | 36.2 | excluded |
| OprD | Q02IC0 | *oprD* | SGTGNLPVMNDGTPR | 758.365 | 530.257 | 11.2 | 36.2 | excluded |
| OprD | P32722 | *oprD* | TGTGNLPVMNDGKPR | 519.6 | 686.358 | 9.9 | 25.9 | excluded |
| OprD | P32722 | *oprD* | TGTGNLPVMNDGKPR | 519.6 | 457.288 | 9.9 | 25.9 | excluded |
| OprD | P32722 | *oprD* | TGTGNLPVMNDGKPR | 519.6 | 507.263 | 9.9 | 25.9 | excluded |
| OprD | Q6QR54 | *oprD* | VDSSSSYAGLYGEDGK | 817.863 | 909.431 | 11.6 | 38.3 | conserved |
| OprD | Q6QR54 | *oprD* | VDSSSSYAGLYGEDGK | 817.863 | 838.394 | 11.6 | 38.3 | conserved |
| OprD | Q6QR54 | *oprD* | VDSSSSYAGLYGEDGK | 817.863 | 505.225 | 11.6 | 38.3 | conserved |
| OprD | P32722 | *oprD* | VHGDQPFDYIGFGR | 536.592 | 549.314 | 15.9 | 26.8 | conserved |
| OprD | P32722 | *oprD* | VHGDQPFDYIGFGR | 536.592 | 436.23 | 15.9 | 26.8 | conserved |
| OprD | P32722 | *oprD* | VHGDQPFDYIGFGR | 536.592 | 537.242 | 15.9 | 26.8 | conserved |
| OprD | Q6QR54 | *oprD* | YVVQAGPAK | 466.764 | 670.388 | 7.5 | 25.7 | conserved |
| OprD | Q6QR54 | *oprD* | YVVQAGPAK | 466.764 | 571.32 | 7.5 | 25.7 | conserved |
| OprD | Q6QR54 | *oprD* | YVVQAGPAK | 466.764 | 443.261 | 7.5 | 25.7 | conserved |
| OprD | P32722 | *oprD* | YVVQSGPAK | 474.761 | 686.383 | 7.1 | 25.9 | conserved |
| OprD | P32722 | *oprD* | YVVQSGPAK | 474.761 | 587.315 | 7.1 | 25.9 | conserved |
| OprD | P32722 | *oprD* | YVVQSGPAK | 474.761 | 459.256 | 7.1 | 25.9 | conserved |

Table S6 contiuation

| Protein name | Accession number | Gene name | Proteotypic peptide sequence | Q1 - Precursor ion (m/z) | Q3 - Fragment ion (m/z) | Target retention time (min) | Collision energy (Volts) | Conserved versus excluded transitions (literature-based analysis) |
|---|---|---|---|---|---|---|---|---|
| OprF | P13794 | *oprF* | ASLDGQYGLEK | 590.796 | 909.431 | 10.6 | 30.1 | conserved |
| OprF | P13794 | *oprF* | ASLDGQYGLEK | 590.796 | 794.404 | 10.6 | 30.1 | conserved |
| OprF | P13794 | *oprF* | ASLDGQYGLEK | 590.796 | 609.324 | 10.6 | 30.1 | conserved |
| OprF | P13794 | *oprF* | DVLVNEYGVEGGR | 703.849 | 980.443 | 13.2 | 34.2 | conserved |
| OprF | P13794 | *oprF* | DVLVNEYGVEGGR | 703.849 | 737.358 | 13.2 | 34.2 | conserved |
| OprF | P13794 | *oprF* | DVLVNEYGVEGGR | 703.849 | 328.187 | 13.2 | 34.2 | conserved |
| OprF | P13794 | *oprF* | DVLVNEYGVEGGR | 469.569 | 980.443 | 13.2 | 23.1 | conserved |
| OprF | P13794 | *oprF* | DVLVNEYGVEGGR | 469.569 | 574.294 | 13.2 | 23.1 | conserved |
| OprF | P13794 | *oprF* | DVLVNEYGVEGGR | 469.569 | 418.204 | 13.2 | 23.1 | conserved |
| OprF | P13794 | *oprF* | QQMTMANIGAGLK | 681.847 | 975.529 | 13.1 | 33.4 | excluded |
| OprF | P13794 | *oprF* | QQMTMANIGAGLK | 681.847 | 874.482 | 13.1 | 33.4 | excluded |
| OprF | P13794 | *oprF* | QQMTMANIGAGLK | 681.847 | 672.404 | 13.1 | 33.4 | excluded |
| OprF | P13794 | *oprF* | VEAEVEAEAK | 537.769 | 975.463 | 6 | 28.2 | conserved |
| OprF | P13794 | *oprF* | VEAEVEAEAK | 537.769 | 846.42 | 6 | 28.2 | conserved |
| OprF | P13794 | *oprF* | VEAEVEAEAK | 537.769 | 646.341 | 6 | 28.2 | conserved |
| OprF | P13794 | *oprF* | YYFTENFFAK | 665.311 | 856.42 | 17.3 | 32.8 | conserved |
| OprF | P13794 | *oprF* | YYFTENFFAK | 665.311 | 755.372 | 17.3 | 32.8 | conserved |
| OprF | P13794 | *oprF* | YYFTENFFAK | 665.311 | 365.218 | 17.3 | 32.8 | conserved |
| OprJ | Q51397 | *oprJ* | EVADALAASDTLR | 666.344 | 846.468 | 13.1 | 32.8 | conserved |
| OprJ | Q51397 | *oprJ* | EVADALAASDTLR | 666.344 | 733.384 | 13.1 | 32.8 | conserved |
| OprJ | Q51397 | *oprJ* | EVADALAASDTLR | 666.344 | 662.347 | 13.1 | 32.8 | conserved |
| OprJ | Q51397 | *oprJ* | EYSFALIDQR | 621.312 | 949.51 | 14.7 | 31.2 | conserved |
| OprJ | Q51397 | *oprJ* | EYSFALIDQR | 621.312 | 715.41 | 14.7 | 31.2 | conserved |
| OprJ | Q51397 | *oprJ* | EYSFALIDQR | 621.312 | 644.373 | 14.7 | 31.2 | conserved |
| OprJ | Q51397 | *oprJ* | QIALVDLFR | 537.819 | 833.488 | 18 | 28.2 | excluded |
| OprJ | Q51397 | *oprJ* | QIALVDLFR | 537.819 | 649.367 | 18 | 28.2 | excluded |
| OprJ | Q51397 | *oprJ* | QIALVDLFR | 537.819 | 435.271 | 18 | 28.2 | excluded |
| OprJ | Q51397 | *oprJ* | QTLLDIEAAR | 565.314 | 787.431 | 13.7 | 29.2 | excluded |
| OprJ | Q51397 | *oprJ* | QTLLDIEAAR | 565.314 | 674.347 | 13.7 | 29.2 | excluded |
| OprJ | Q51397 | *oprJ* | QTLLDIEAAR | 565.314 | 559.32 | 13.7 | 29.2 | excluded |
| OprJ | Q51397 | *oprJ* | SSFLNEIAFIDGSTQR | 595.632 | 776.39 | 21.8 | 30 | conserved |
| OprJ | Q51397 | *oprJ* | SSFLNEIAFIDGSTQR | 595.632 | 663.306 | 21.8 | 30 | conserved |
| OprJ | Q51397 | *oprJ* | SSFLNEIAFIDGSTQR | 595.632 | 862.43 | 21.8 | 30 | excluded |
| OprM | Q51487 | *oprM* | ADQAQLQLTK | 558.306 | 801.483 | 9 | 29 | conserved |
| OprM | Q51487 | *oprM* | ADQAQLQLTK | 558.306 | 602.387 | 9 | 29 | conserved |
| OprM | Q51487 | *oprM* | ADQAQLQLTK | 558.306 | 361.245 | 9 | 29 | conserved |
| OprM | Q51487 | *oprM* | AIQTAFQEVADGLAAR | 554.293 | 673.363 | 20 | 27.7 | conserved |
| OprM | Q51487 | *oprM* | AIQTAFQEVADGLAAR | 554.293 | 602.326 | 20 | 27.7 | conserved |
| OprM | Q51487 | *oprM* | AIQTAFQEVADGLAAR | 554.293 | 487.299 | 20 | 27.7 | conserved |
| OprM | Q51487 | *oprM* | DQALEQYLATEQAQR | 882.432 | 916.485 | 13.6 | 40.6 | conserved |
| OprM | Q51487 | *oprM* | DQALEQYLATEQAQR | 882.432 | 803.401 | 13.6 | 40.6 | conserved |
| OprM | Q51487 | *oprM* | DQALEQYLATEQAQR | 882.432 | 315.13 | 13.6 | 40.6 | conserved |
| OprM | Q51487 | *oprM* | GTFTEQLQAQR | 639.826 | 973.506 | 11.1 | 31.9 | conserved |
| OprM | Q51487 | *oprM* | GTFTEQLQAQR | 639.826 | 743.416 | 11.1 | 31.9 | conserved |
| OprM | Q51487 | *oprM* | GTFTEQLQAQR | 639.826 | 615.357 | 11.1 | 31.9 | conserved |
| OprM | Q51487 | *oprM* | LNQLTSEVNLYK | 711.385 | 953.494 | 13.8 | 34.5 | conserved |
| OprM | Q51487 | *oprM* | LNQLTSEVNLYK | 711.385 | 852.446 | 13.8 | 34.5 | conserved |
| OprM | Q51487 | *oprM* | LNQLTSEVNLYK | 711.385 | 356.193 | 13.8 | 34.5 | conserved |
| OprM | Q51487 | *oprM* | SLFTAQQQLITDR | 760.907 | 873.479 | 15 | 36.2 | conserved |
| OprM | Q51487 | *oprM* | SLFTAQQQLITDR | 760.907 | 617.362 | 15 | 36.2 | conserved |
| OprM | Q51487 | *oprM* | SLFTAQQQLITDR | 760.907 | 391.194 | 15 | 36.2 | conserved |
| OprM | Q51487 | *oprM* | SYDVGVASALDLR | 683.354 | 901.51 | 16.1 | 33.5 | conserved |
| OprM | Q51487 | *oprM* | SYDVGVASALDLR | 683.354 | 745.42 | 16.1 | 33.5 | conserved |
| OprM | Q51487 | *oprM* | SYDVGVASALDLR | 683.354 | 366.13 | 16.1 | 33.5 | conserved |
| OprM | Q51487 | *oprM* | TGVDNYLTLLDAQR | 789.91 | 929.541 | 19.6 | 37.3 | conserved |
| OprM | Q51487 | *oprM* | TGVDNYLTLLDAQR | 789.91 | 816.457 | 19.6 | 37.3 | conserved |
| OprM | Q51487 | *oprM* | TGVDNYLTLLDAQR | 789.91 | 602.326 | 19.6 | 37.3 | conserved |
| OprM | Q51487 | *oprM* | VAALNVEAFR | 545.306 | 990.537 | 15.8 | 28.5 | conserved |
| OprM | Q51487 | *oprM* | VAALNVEAFR | 545.306 | 848.462 | 15.8 | 28.5 | conserved |
| OprM | Q51487 | *oprM* | VAALNVEAFR | 545.306 | 735.378 | 15.8 | 28.5 | conserved |

Table S6 contiuation

| Protein name | Accession number | Gene name | Proteotypic peptide sequence | Q1 - Precursor ion (m/z) | Q3 - Fragment ion (m/z) | Target retention time (min) | Collision energy (Volts) | Conserved versus excluded transitions (literature-based analysis) |
|---|---|---|---|---|---|---|---|---|
| OprN | Q9I0Y7 | *oprN* | AAAQQAAIR | 450.257 | 757.432 | 5.7 | 25.1 | conserved |
| OprN | Q9I0Y7 | *oprN* | AAAQQAAIR | 450.257 | 686.394 | 5.7 | 25.1 | conserved |
| OprN | Q9I0Y7 | *oprN* | AAAQQAAIR | 450.257 | 558.336 | 5.7 | 25.1 | conserved |
| OprN | Q9I0Y7 | *oprN* | ALPIGDPGELLR | 625.859 | 856.452 | 18.5 | 31.4 | conserved |
| OprN | Q9I0Y7 | *oprN* | ALPIGDPGELLR | 625.859 | 684.404 | 18.5 | 31.4 | conserved |
| OprN | Q9I0Y7 | *oprN* | ALPIGDPGELLR | 625.859 | 533.798 | 18.5 | 31.4 | conserved |
| OprN | Q9I0Y7 | *oprN* | DAGVGAELDVLR | 607.822 | 872.484 | 16.5 | 30.7 | conserved |
| OprN | Q9I0Y7 | *oprN* | DAGVGAELDVLR | 607.822 | 815.462 | 16.5 | 30.7 | conserved |
| OprN | Q9I0Y7 | *oprN* | DAGVGAELDVLR | 607.822 | 343.161 | 16.5 | 30.7 | conserved |
| OprN | Q9I0Y7 | *oprN* | IALSNLENQK | 565.314 | 945.5 | 12.5 | 29.2 | conserved |
| OprN | Q9I0Y7 | *oprN* | IALSNLENQK | 565.314 | 832.416 | 12.5 | 29.2 | conserved |
| OprN | Q9I0Y7 | *oprN* | IALSNLENQK | 565.314 | 631.341 | 12.5 | 29.2 | excluded |
| OprN | Q9I0Y7 | *oprN* | IATLLGQRPEELTVDLSPR | 703.395 | 587.315 | 18.5 | 35.9 | conserved |
| OprN | Q9I0Y7 | *oprN* | IATLLGQRPEELTVDLSPR | 703.395 | 962.529 | 18.5 | 35.9 | conserved |
| OprN | Q9I0Y7 | *oprN* | IATLLGQRPEELTVDLSPR | 703.395 | 798.921 | 18.5 | 35.9 | conserved |
| OprN | Q9I0Y7 | *oprN* | IDATASKPYDR | 412.879 | 550.262 | 6.7 | 20.1 | conserved |
| OprN | Q9I0Y7 | *oprN* | IDATASKPYDR | 412.879 | 562.273 | 6.7 | 20.1 | conserved |
| OprN | Q9I0Y7 | *oprN* | IDATASKPYDR | 412.879 | 504.759 | 6.7 | 20.1 | conserved |
| ModA | A3KT69 | *modA* | LEQEGEIVPGSR | 657.338 | 814.442 | 10 | 32.5 | conserved |
| ModA | A3KT69 | *modA* | LEQEGEIVPGSR | 657.338 | 515.294 | 10 | 32.5 | conserved |
| ModA | A3KT69 | *modA* | LEQEGEIVPGSR | 657.338 | 416.225 | 10 | 32.5 | conserved |
| GnyD | A3KTK8 | *gnyD* | GFVAAELQPR | 544.298 | 784.431 | 12.6 | 28.5 | conserved |
| GnyD | A3KTK8 | *gnyD* | GFVAAELQPR | 544.298 | 713.394 | 12.6 | 28.5 | conserved |
| GnyD | A3KTK8 | *gnyD* | GFVAAELQPR | 544.298 | 642.357 | 12.6 | 28.5 | conserved |
| putative uncharacterized protein | A3KU99 | *uncharacterized* | LGGAGGTLADAVER | 643.839 | 988.506 | 11.7 | 32 | conserved |
| putative uncharacterized protein | A3KU99 | *uncharacterized* | LGGAGGTLADAVER | 643.839 | 931.484 | 11.7 | 32 | conserved |
| putative uncharacterized protein | A3KU99 | *uncharacterized* | LGGAGGTLADAVER | 643.839 | 660.331 | 11.7 | 32 | conserved |
| putative uncharacterized protein | A3KVW0 | *uncharacterized* | TGLTDIDDVVPFIR | 780.917 | 960.515 | 19.9 | 37 | conserved |
| putative uncharacterized protein | A3KVW0 | *uncharacterized* | TGLTDIDDVVPFIR | 780.917 | 631.393 | 19.9 | 37 | conserved |
| putative uncharacterized protein | A3KVW0 | *uncharacterized* | TGLTDIDDVVPFIR | 780.917 | 532.324 | 19.9 | 37 | conserved |
| N-succinylglutamate 5-semialdehyde dehydrogenase | A3L5G2 | *astD* | GADATQVDAAVR | 587.297 | 859.463 | 7.7 | 30 | conserved |
| N-succinylglutamate 5-semialdehyde dehydrogenase | A3L5G2 | *astD* | GADATQVDAAVR | 587.297 | 758.416 | 7.7 | 30 | conserved |
| N-succinylglutamate 5-semialdehyde dehydrogenase | A3L5G2 | *astD* | GADATQVDAAVR | 587.297 | 416.262 | 7.7 | 30 | conserved |
| putative uncharacterized protein | A3L8T1 | *uncharacterized* | EDPIVIVSAVR | 599.346 | 743.477 | 14.4 | 30.4 | conserved |
| putative uncharacterized protein | A3L8T1 | *uncharacterized* | EDPIVIVSAVR | 599.346 | 644.409 | 14.4 | 30.4 | conserved |
| putative uncharacterized protein | A3L8T1 | *uncharacterized* | EDPIVIVSAVR | 599.346 | 531.325 | 14.4 | 30.4 | conserved |

| Table S6 contiuation | | | | | | | | |
|---|---|---|---|---|---|---|---|---|
| Protein name | Accession number | Gene name | Proteotypic peptide sequence | Q1 - Precursor ion (m/z) | Q3 - Fragment ion (m/z) | Target retention time (min) | Collision energy (Volts) | Conserved versus excluded transitions (literature-based analysis) |
| aminopeptidase N | A3LA70 | *pepN* | LEGEAVAQGGNR | 600.802 | 958.47 | 6.1 | 30.5 | conserved |
| aminopeptidase N | A3LA70 | *pepN* | LEGEAVAQGGNR | 600.802 | 602.3 | 6.1 | 30.5 | conserved |
| aminopeptidase N | A3LA70 | *pepN* | LEGEAVAQGGNR | 600.802 | 531.263 | 6.1 | 30.5 | conserved |
| Acetyltransferase component of pyruvate dehydrogenase complex | A3LJB0 | *aceF* | ADTPAPVGAPSR | 569.796 | 851.473 | 6.8 | 29.4 | conserved |
| Acetyltransferase component of pyruvate dehydrogenase complex | A3LJB0 | *aceF* | ADTPAPVGAPSR | 569.796 | 683.383 | 6.8 | 29.4 | conserved |
| Acetyltransferase component of pyruvate dehydrogenase complex | A3LJB0 | *aceF* | ADTPAPVGAPSR | 569.796 | 487.262 | 6.8 | 29.4 | conserved |
| Insulin-cleaving metalloproteinase outer membrane protein | A3LLD0 | *icmP* | ADAAANDTLK | 495.249 | 803.426 | 6.5 | 26.7 | conserved |
| Insulin-cleaving metalloproteinase outer membrane protein | A3LLD0 | *icmP* | ADAAANDTLK | 495.249 | 732.389 | 6.5 | 26.7 | conserved |
| Insulin-cleaving metalloproteinase outer membrane protein | A3LLD0 | *icmP* | ADAAANDTLK | 495.249 | 661.352 | 6.5 | 26.7 | conserved |
| B-type flagellin | P72151 | *fliC* | GVLTITSATGENVK | 695.383 | 906.453 | 12.3 | 33.9 | excluded |
| B-type flagellin | P72151 | *fliC* | GVLTITSATGENVK | 695.383 | 805.405 | 12.3 | 33.9 | excluded |
| B-type flagellin | P72151 | *fliC* | GVLTITSATGENVK | 695.383 | 546.288 | 12.3 | 33.9 | excluded |
| B-type flagellin | P72151 | *fliC* | LGITASINDK | 516.29 | 918.489 | 10.6 | 27.4 | excluded |
| B-type flagellin | P72151 | *fliC* | LGITASINDK | 516.29 | 748.384 | 10.6 | 27.4 | excluded |
| B-type flagellin | P72151 | *fliC* | LGITASINDK | 516.29 | 647.336 | 10.6 | 27.4 | excluded |
| B-type flagellin | P72151 | *fliC* | LSNQISGLNVATR | 686.881 | 930.537 | 12.8 | 33.6 | excluded |
| B-type flagellin | P72151 | *fliC* | LSNQISGLNVATR | 686.881 | 817.453 | 12.8 | 33.6 | excluded |
| B-type flagellin | P72151 | *fliC* | LSNQISGLNVATR | 686.881 | 730.421 | 12.8 | 33.6 | excluded |
| B-type flagellin | P72151 | *fliC* | NIAIAAGDSAK | 515.78 | 803.426 | 8.4 | 27.4 | excluded |
| B-type flagellin | P72151 | *fliC* | NIAIAAGDSAK | 515.78 | 732.389 | 8.4 | 27.4 | excluded |
| B-type flagellin | P72151 | *fliC* | NIAIAAGDSAK | 515.78 | 619.305 | 8.4 | 27.4 | excluded |
| 50S ribosomal protein L25 | Q9HVC4 | *rplY* | QAGGEISHTISEVEVSCLPK | 714.356 | 832.423 | 14.8 | 36.5 | conserved |
| 50S ribosomal protein L25 | Q9HVC4 | *rplY* | QAGGEISHTISEVEVSCLPK | 714.356 | 703.381 | 14.8 | 36.5 | conserved |
| 50S ribosomal protein L25 | Q9HVC4 | *rplY* | QAGGEISHTISEVEVSCLPK | 714.356 | 604.312 | 14.8 | 36.5 | conserved |

**Supplementary Table 7. Evaluation of quantotypic peptides by calculating pairwise variation.** A stepwise exclusion was performed until internal control protein-stability measures were below 0.7.

| | OprF DVLVNEYGVEGGR | A3KT69 LEQEGEIVPGSR | A3KTK8 GFVAAELQPR | A3KU99 LGGAGGTLADAVER | A3KVW0 TGLTDIDDVVPFIR | A3L5G2 GADATQVDAAVR | A3L8T1 EDPIVIVSAVR | A3LA70 LEGEAVAQGGNR | A3LJB0 ADTPAPVGAPSR | A3LLD0 ADAAANDTLK | RL25 QAGGEISHTISEVEVSCLPK | Internal control protein-stability measure |
|---|---|---|---|---|---|---|---|---|---|---|---|---|
| OprF DVLVNEYGVEGGR | 0.10 | 0.52 | 1.42 | 0.68 | 1.16 | 1.93 | 0.99 | 0.42 | 0.27 | 0.38 | 0.98 | included |
| OprF DVLVNEYGVEGGR | 0.09 | 0.53 | 1.41 | 0.64 | 1.14 | 1.96 | 0.95 | 0.38 | 0.24 | 0.37 | 0.98 | included |
| OprF DVLVNEYGVEGGR | 0.08 | 0.53 | 1.43 | 0.67 | 1.17 | 1.93 | 0.98 | 0.41 | 0.27 | 0.40 | 1.01 | included |
| A3KT69 LEQEGEIVPGSR | 0.52 | 0.16 | 1.52 | 1.10 | 1.50 | 2.24 | 1.27 | 0.77 | 0.65 | 0.56 | 1.04 | included |
| A3KT69 LEQEGEIVPGSR | 0.59 | 0.18 | 1.58 | 1.16 | 1.54 | 2.29 | 1.33 | 0.83 | 0.70 | 0.61 | 1.08 | included |
| A3KT69 LEQEGEIVPGSR | 0.47 | 0.17 | 1.53 | 1.03 | 1.42 | 2.21 | 1.22 | 0.69 | 0.56 | 0.47 | 0.96 | included |
| A3KTK8 GFVAAELQPR | 1.43 | 1.55 | 0.04 | 1.30 | 1.27 | 2.33 | 1.10 | 1.32 | 1.49 | 1.63 | 1.43 | excluded |
| A3KTK8 GFVAAELQPR | 1.41 | 1.54 | 0.06 | 1.28 | 1.26 | 2.32 | 1.08 | 1.30 | 1.47 | 1.61 | 1.43 | excluded |
| A3KTK8 GFVAAELQPR | 1.42 | 1.55 | 0.06 | 1.31 | 1.28 | 2.32 | 1.10 | 1.32 | 1.48 | 1.63 | 1.43 | excluded |
| A3KU99 LGGAGGTLADAVER | 0.63 | 1.04 | 1.27 | 0.22 | 0.74 | 1.81 | 0.74 | 0.38 | 0.52 | 0.69 | 0.92 | included |
| A3KU99 LGGAGGTLADAVER | 0.67 | 1.14 | 1.35 | 0.25 | 0.73 | 1.81 | 0.73 | 0.45 | 0.56 | 0.77 | 1.04 | included |
| A3KU99 LGGAGGTLADAVER | 0.69 | 1.11 | 1.26 | 0.20 | 0.68 | 1.84 | 0.68 | 0.43 | 0.58 | 0.76 | 0.96 | included |
| A3KVW0 TGLTDIDDVVPFIR | 1.18 | 1.49 | 1.40 | 0.76 | 0.42 | 2.15 | 0.66 | 0.82 | 1.01 | 1.22 | 0.85 | excluded |
| A3KVW0 TGLTDIDDVVPFIR | 1.29 | 1.61 | 1.14 | 0.85 | 0.49 | 2.12 | 0.67 | 0.96 | 1.18 | 1.42 | 1.10 | excluded |
| A3KVW0 TGLTDIDDVVPFIR | 1.01 | 1.35 | 1.28 | 0.53 | 0.39 | 2.02 | 0.60 | 0.64 | 0.84 | 1.06 | 0.82 | excluded |
| A3L5G2 GADATQVDAAVR | 2.09 | 2.39 | 2.48 | 1.98 | 2.26 | 0.44 | 2.52 | 2.15 | 2.10 | 2.14 | 2.29 | excluded |
| A3L5G2 GADATQVDAAVR | 2.21 | 2.52 | 2.65 | 2.12 | 2.41 | 0.53 | 2.66 | 2.29 | 2.23 | 2.26 | 2.45 | excluded |
| A3L5G2 GADATQVDAAVR | 1.53 | 1.85 | 1.84 | 1.37 | 1.62 | 0.77 | 1.87 | 1.54 | 1.53 | 1.60 | 1.68 | excluded |
| A3L8T1 EDPIVIVSAVR | 1.03 | 1.32 | 1.11 | 0.75 | 0.62 | 2.39 | 0.16 | 0.73 | 0.95 | 1.18 | 1.05 | excluded |
| A3L8T1 EDPIVIVSAVR | 0.96 | 1.24 | 1.04 | 0.70 | 0.60 | 2.31 | 0.16 | 0.67 | 0.88 | 1.13 | 1.01 | excluded |
| A3L8T1 EDPIVIVSAVR | 0.94 | 1.25 | 1.12 | 0.71 | 0.71 | 2.36 | 0.18 | 0.68 | 0.89 | 1.13 | 1.12 | excluded |
| A3LA70 LEGEAVAQGGNR | 0.41 | 0.78 | 1.35 | 0.40 | 0.80 | 1.97 | 0.72 | 0.08 | 0.23 | 0.48 | 0.78 | included |
| A3LA70 LEGEAVAQGGNR | 0.41 | 0.76 | 1.30 | 0.43 | 0.80 | 2.01 | 0.68 | 0.07 | 0.26 | 0.50 | 0.76 | included |
| A3LA70 LEGEAVAQGGNR | 0.38 | 0.75 | 1.29 | 0.43 | 0.82 | 2.00 | 0.68 | 0.07 | 0.25 | 0.50 | 0.79 | included |
| A3LJB0 ADTPAPVGAPSR | 0.23 | 0.60 | 1.48 | 0.58 | 1.03 | 1.97 | 0.92 | 0.27 | 0.08 | 0.29 | 0.85 | included |
| A3LJB0 ADTPAPVGAPSR | 0.26 | 0.64 | 1.48 | 0.55 | 1.01 | 1.94 | 0.92 | 0.25 | 0.07 | 0.30 | 0.85 | included |
| A3LJB0 ADTPAPVGAPSR | 0.28 | 0.67 | 1.48 | 0.52 | 0.99 | 1.95 | 0.89 | 0.23 | 0.08 | 0.32 | 0.86 | included |
| A3LLD0 ADAAANDTLK | 0.39 | 0.53 | 1.64 | 0.77 | 1.27 | 2.01 | 1.18 | 0.53 | 0.33 | 0.07 | 0.93 | included |
| A3LLD0 ADAAANDTLK | 0.38 | 0.55 | 1.61 | 0.73 | 1.22 | 2.00 | 1.13 | 0.48 | 0.29 | 0.05 | 0.90 | included |
| A3LLD0 ADAAANDTLK | 0.38 | 0.57 | 1.62 | 0.72 | 1.21 | 1.99 | 1.13 | 0.47 | 0.28 | 0.06 | 0.89 | included |
| RL25 QAGGEISHTISEVEVSCLPK | 1.04 | 1.08 | 1.48 | 1.00 | 0.93 | 2.16 | 1.09 | 0.82 | 0.89 | 0.95 | 0.17 | excluded |
| RL25 QAGGEISHTISEVEVSCLPK | 0.97 | 1.00 | 1.41 | 0.96 | 0.92 | 2.13 | 1.05 | 0.76 | 0.83 | 0.89 | 0.13 | excluded |
| RL25 QAGGEISHTISEVEVSCLPK | 0.96 | 0.99 | 1.40 | 0.96 | 0.92 | 2.12 | 1.04 | 0.75 | 0.83 | 0.89 | 0.12 | excluded |

**Supplementary Table 8. Pearson correlation of log-normalized areas between transitions among efflux systems (A-H) and AmpC cephalosporinase (I).** A critical value of >0.8 was fixed to be a relevant transition as surrogate of protein level. Critical values <0.8 appeared in blue whereas critical values >0.8 appeared in red.

**(A)** MexA

| | MexA AILAPQQGVTR | MexA AILAPQQGVTR | MexA AILAPQQGVTR | MexA GQATALVVNAQNK | MexA GQATALVVNAQNK | MexA GQATALVVNAQNK | MexA IITEGLQFVQPGVEVK | MexA IITEGLQFVQPGVEVK | MexA IITEGLQFVQPGVEVK | MexA LEDGSQYPLEGR | MexA LEDGSQYPLEGR | MexA LEDGSQYPLEGR |
|---|---|---|---|---|---|---|---|---|---|---|---|---|
| MexA AILAPQQGVTR | 1.0 | 1.0 | 1.0 | 0.9 | 0.9 | 0.9 | 1.0 | 1.0 | 1.0 | 1.0 | 1.0 | 1.0 |
| MexA AILAPQQGVTR | 1.0 | 1.0 | 1.0 | 0.9 | 0.9 | 0.9 | 1.0 | 1.0 | 1.0 | 1.0 | 1.0 | 1.0 |
| MexA AILAPQQGVTR | 1.0 | 1.0 | 1.0 | 0.9 | 0.9 | 0.9 | 1.0 | 1.0 | 1.0 | 1.0 | 1.0 | 1.0 |
| MexA GQATALVVNAQNK | 0.9 | 0.9 | 0.9 | 1.0 | 1.0 | 1.0 | 1.0 | 1.0 | 1.0 | 0.9 | 1.0 | 1.0 |
| MexA GQATALVVNAQNK | 0.9 | 0.9 | 0.9 | 1.0 | 1.0 | 1.0 | 1.0 | 1.0 | 1.0 | 0.9 | 1.0 | 1.0 |
| MexA GQATALVVNAQNK | 0.9 | 0.9 | 0.9 | 1.0 | 1.0 | 1.0 | 1.0 | 1.0 | 1.0 | 0.9 | 1.0 | 1.0 |
| MexA IITEGLQFVQPGVEVK | 1.0 | 1.0 | 1.0 | 1.0 | 1.0 | 1.0 | 1.0 | 1.0 | 1.0 | 1.0 | 1.0 | 1.0 |
| MexA IITEGLQFVQPGVEVK | 1.0 | 1.0 | 1.0 | 1.0 | 1.0 | 1.0 | 1.0 | 1.0 | 1.0 | 1.0 | 1.0 | 1.0 |
| MexA IITEGLQFVQPGVEVK | 1.0 | 1.0 | 1.0 | 1.0 | 1.0 | 1.0 | 1.0 | 1.0 | 1.0 | 1.0 | 1.0 | 1.0 |
| MexA LEDGSQYPLEGR | 1.0 | 1.0 | 1.0 | 0.9 | 0.9 | 0.9 | 1.0 | 1.0 | 1.0 | 1.0 | 1.0 | 1.0 |
| MexA LEDGSQYPLEGR | 1.0 | 1.0 | 1.0 | 1.0 | 1.0 | 1.0 | 1.0 | 1.0 | 1.0 | 1.0 | 1.0 | 1.0 |
| MexA LEDGSQYPLEGR | 1.0 | 1.0 | 1.0 | 1.0 | 1.0 | 1.0 | 1.0 | 1.0 | 1.0 | 1.0 | 1.0 | 1.0 |

**(B)** MexB

| | MexB EDLSNYIVSNIQDPLSR | MexB EDLSNYIVSNIQDPLSR | MexB EDLSNYIVSNIQDPLSR | MexB GQQLNATIIGK | MexB GQQLNATIIGK | MexB GQQLNATIIGK | MexB LATGANALDTAK | MexB LATGANALDTAK | MexB LATGANALDTAK | MexB LQLATPLLPQEVQR | MexB LQLATPLLPQEVQR | MexB LQLATPLLPQEVQR | MexB NAILIVEFAK | MexB NAILIVEFAK | MexB NAILIVEFAK |
|---|---|---|---|---|---|---|---|---|---|---|---|---|---|---|---|
| MexB EDLSNYIVSNIQDPLSR | 1.0 | 1.0 | 1.0 | 0.9 | 0.9 | 0.9 | 0.9 | 0.9 | 0.9 | 0.9 | 0.9 | 0.9 | 0.7 | 0.7 | 0.7 |
| MexB EDLSNYIVSNIQDPLSR | 1.0 | 1.0 | 1.0 | 0.9 | 0.9 | 0.9 | 0.9 | 0.9 | 0.9 | 0.9 | 0.9 | 0.9 | 0.7 | 0.7 | 0.7 |
| MexB EDLSNYIVSNIQDPLSR | 1.0 | 1.0 | 1.0 | 0.9 | 0.9 | 0.9 | 0.9 | 0.9 | 0.9 | 0.9 | 0.9 | 0.9 | 0.7 | 0.7 | 0.7 |
| MexB GQQLNATIIGK | 0.9 | 0.9 | 0.9 | 1.0 | 1.0 | 1.0 | 1.0 | 1.0 | 1.0 | 1.0 | 1.0 | 1.0 | 0.7 | 0.7 | 0.7 |
| MexB GQQLNATIIGK | 0.9 | 0.9 | 0.9 | 1.0 | 1.0 | 1.0 | 1.0 | 1.0 | 1.0 | 1.0 | 1.0 | 1.0 | 0.7 | 0.7 | 0.7 |
| MexB GQQLNATIIGK | 0.9 | 0.9 | 0.9 | 1.0 | 1.0 | 1.0 | 1.0 | 1.0 | 1.0 | 1.0 | 1.0 | 1.0 | 0.7 | 0.7 | 0.7 |
| MexB LATGANALDTAK | 0.9 | 0.9 | 0.9 | 1.0 | 1.0 | 1.0 | 1.0 | 1.0 | 1.0 | 1.0 | 1.0 | 1.0 | 0.6 | 0.6 | 0.6 |
| MexB LATGANALDTAK | 0.9 | 0.9 | 0.9 | 1.0 | 1.0 | 1.0 | 1.0 | 1.0 | 1.0 | 1.0 | 1.0 | 1.0 | 0.6 | 0.6 | 0.6 |
| MexB LATGANALDTAK | 0.9 | 0.9 | 0.9 | 1.0 | 1.0 | 1.0 | 1.0 | 1.0 | 1.0 | 1.0 | 1.0 | 1.0 | 0.6 | 0.6 | 0.6 |
| MexB LQLATPLLPQEVQR | 0.9 | 0.9 | 0.9 | 1.0 | 1.0 | 1.0 | 1.0 | 1.0 | 1.0 | 1.0 | 1.0 | 1.0 | 0.7 | 0.7 | 0.7 |
| MexB LQLATPLLPQEVQR | 0.9 | 0.9 | 0.9 | 1.0 | 1.0 | 1.0 | 1.0 | 1.0 | 1.0 | 1.0 | 1.0 | 1.0 | 0.7 | 0.7 | 0.7 |
| MexB LQLATPLLPQEVQR | 0.9 | 0.9 | 0.9 | 1.0 | 1.0 | 1.0 | 1.0 | 1.0 | 1.0 | 1.0 | 1.0 | 1.0 | 0.7 | 0.7 | 0.7 |
| MexB NAILIVEFAK | 0.7 | 0.7 | 0.7 | 0.7 | 0.7 | 0.7 | 0.6 | 0.6 | 0.6 | 0.7 | 0.7 | 0.7 | 1.0 | 1.0 | 1.0 |
| MexB NAILIVEFAK | 0.7 | 0.7 | 0.7 | 0.7 | 0.7 | 0.7 | 0.6 | 0.6 | 0.6 | 0.7 | 0.7 | 0.7 | 1.0 | 1.0 | 1.0 |
| MexB NAILIVEFAK | 0.7 | 0.7 | 0.7 | 0.7 | 0.7 | 0.7 | 0.6 | 0.6 | 0.6 | 0.7 | 0.7 | 0.7 | 1.0 | 1.0 | 1.0 |

**(C)** OprM

| | OprM ADQAQLQLTK | OprM ADQAQLQLTK | OprM ADQAQLQLTK | OprM AIQTAFQEVADGLAAR | OprM AIQTAFQEVADGLAAR | OprM AIQTAFQEVADGLAAR | OprM DQALEQYLATEQAQR | OprM DQALEQYLATEQAQR | OprM DQALEQYLATEQAQR | OprM GTFTEQLQAQR | OprM GTFTEQLQAQR | OprM GTFTEQLQAQR | OprM LNQLTSEVNLYK | OprM LNQLTSEVNLYK | OprM LNQLTSEVNLYK | OprM SLFTAQQQLITDR | OprM SLFTAQQQLITDR | OprM SLFTAQQQLITDR | OprM SYDVGVASALDLR | OprM SYDVGVASALDLR | OprM SYDVGVASALDLR | OprM TGVDNYLTLLDAQR | OprM TGVDNYLTLLDAQR | OprM TGVDNYLTLLDAQR | OprM VAALNVEAFR | OprM VAALNVEAFR | OprM VAALNVEAFR |
|---|---|---|---|---|---|---|---|---|---|---|---|---|---|---|---|---|---|---|---|---|---|---|---|---|---|---|---|
| ADQ… | 1.0 | 1.0 | 1.0 | 1.0 | 1.0 | 1.0 | 1.0 | 1.0 | 1.0 | 1.0 | 1.0 | 1.0 | 1.0 | 1.0 | 1.0 | 1.0 | 1.0 | 1.0 | 1.0 | 1.0 | 1.0 | 1.0 | 1.0 | 1.0 | 1.0 | 1.0 | 1.0 |
| ADQ… | 1.0 | 1.0 | 1.0 | 1.0 | 1.0 | 1.0 | 1.0 | 1.0 | 1.0 | 1.0 | 1.0 | 1.0 | 1.0 | 1.0 | 1.0 | 1.0 | 1.0 | 1.0 | 1.0 | 1.0 | 1.0 | 1.0 | 1.0 | 1.0 | 1.0 | 1.0 | 1.0 |
| ADQ… | 1.0 | 1.0 | 1.0 | 1.0 | 1.0 | 1.0 | 1.0 | 1.0 | 1.0 | 1.0 | 1.0 | 1.0 | 1.0 | 1.0 | 1.0 | 1.0 | 1.0 | 1.0 | 1.0 | 1.0 | 1.0 | 1.0 | 1.0 | 1.0 | 1.0 | 1.0 | 1.0 |
| AIQ… | 1.0 | 1.0 | 1.0 | 1.0 | 1.0 | 1.0 | 1.0 | 1.0 | 1.0 | 1.0 | 1.0 | 1.0 | 1.0 | 1.0 | 1.0 | 1.0 | 1.0 | 1.0 | 1.0 | 1.0 | 1.0 | 1.0 | 1.0 | 1.0 | 1.0 | 1.0 | 1.0 |
| AIQ… | 1.0 | 1.0 | 1.0 | 1.0 | 1.0 | 1.0 | 1.0 | 1.0 | 1.0 | 1.0 | 1.0 | 1.0 | 1.0 | 1.0 | 1.0 | 1.0 | 1.0 | 1.0 | 1.0 | 1.0 | 1.0 | 1.0 | 1.0 | 1.0 | 1.0 | 1.0 | 1.0 |
| AIQ… | 1.0 | 1.0 | 1.0 | 1.0 | 1.0 | 1.0 | 1.0 | 1.0 | 1.0 | 1.0 | 1.0 | 1.0 | 1.0 | 1.0 | 1.0 | 1.0 | 1.0 | 1.0 | 1.0 | 1.0 | 1.0 | 1.0 | 1.0 | 1.0 | 1.0 | 1.0 | 1.0 |
| DQA… | 1.0 | 1.0 | 1.0 | 1.0 | 1.0 | 1.0 | 1.0 | 1.0 | 1.0 | 1.0 | 1.0 | 1.0 | 0.9 | 0.9 | 0.9 | 0.9 | 0.9 | 0.9 | 1.0 | 1.0 | 1.0 | 1.0 | 1.0 | 1.0 | 0.9 | 0.9 | 0.9 |
| DQA… | 1.0 | 1.0 | 1.0 | 1.0 | 1.0 | 1.0 | 1.0 | 1.0 | 1.0 | 1.0 | 1.0 | 1.0 | 1.0 | 1.0 | 1.0 | 0.9 | 0.9 | 0.9 | 1.0 | 1.0 | 1.0 | 1.0 | 1.0 | 1.0 | 0.9 | 0.9 | 0.9 |
| DQA… | 1.0 | 1.0 | 1.0 | 1.0 | 1.0 | 1.0 | 1.0 | 1.0 | 1.0 | 1.0 | 1.0 | 1.0 | 1.0 | 1.0 | 1.0 | 0.9 | 0.9 | 0.9 | 1.0 | 1.0 | 1.0 | 1.0 | 1.0 | 1.0 | 0.9 | 0.9 | 0.9 |
| GFT… | 1.0 | 1.0 | 1.0 | 1.0 | 1.0 | 1.0 | 1.0 | 1.0 | 1.0 | 1.0 | 1.0 | 1.0 | 1.0 | 1.0 | 1.0 | 1.0 | 1.0 | 1.0 | 1.0 | 1.0 | 1.0 | 1.0 | 1.0 | 1.0 | 1.0 | 1.0 | 1.0 |
| GFT… | 1.0 | 1.0 | 1.0 | 1.0 | 1.0 | 1.0 | 1.0 | 1.0 | 1.0 | 1.0 | 1.0 | 1.0 | 1.0 | 1.0 | 1.0 | 1.0 | 1.0 | 1.0 | 1.0 | 1.0 | 1.0 | 1.0 | 1.0 | 1.0 | 0.9 | 0.9 | 1.0 |
| GFT… | 1.0 | 1.0 | 1.0 | 1.0 | 1.0 | 1.0 | 1.0 | 1.0 | 1.0 | 1.0 | 1.0 | 1.0 | 1.0 | 1.0 | 1.0 | 1.0 | 1.0 | 1.0 | 1.0 | 1.0 | 1.0 | 1.0 | 1.0 | 1.0 | 0.9 | 0.9 | 1.0 |
| LNQ… | 1.0 | 1.0 | 1.0 | 1.0 | 1.0 | 1.0 | 0.9 | 1.0 | 1.0 | 1.0 | 1.0 | 1.0 | 1.0 | 1.0 | 1.0 | 1.0 | 1.0 | 1.0 | 1.0 | 1.0 | 1.0 | 1.0 | 1.0 | 1.0 | 1.0 | 1.0 | 1.0 |
| LNQ… | 1.0 | 1.0 | 1.0 | 1.0 | 1.0 | 1.0 | 0.9 | 1.0 | 1.0 | 1.0 | 1.0 | 1.0 | 1.0 | 1.0 | 1.0 | 1.0 | 1.0 | 1.0 | 1.0 | 1.0 | 1.0 | 1.0 | 1.0 | 1.0 | 1.0 | 1.0 | 1.0 |
| LNQ… | 1.0 | 1.0 | 1.0 | 1.0 | 1.0 | 1.0 | 0.9 | 1.0 | 1.0 | 1.0 | 1.0 | 1.0 | 1.0 | 1.0 | 1.0 | 1.0 | 1.0 | 1.0 | 1.0 | 1.0 | 1.0 | 1.0 | 1.0 | 1.0 | 1.0 | 1.0 | 1.0 |
| SLF… | 1.0 | 1.0 | 1.0 | 1.0 | 1.0 | 1.0 | 0.9 | 0.9 | 0.9 | 1.0 | 1.0 | 1.0 | 1.0 | 1.0 | 1.0 | 1.0 | 1.0 | 1.0 | 1.0 | 1.0 | 1.0 | 0.9 | 0.9 | 0.9 | 1.0 | 1.0 | 1.0 |
| SLF… | 1.0 | 1.0 | 1.0 | 1.0 | 1.0 | 1.0 | 0.9 | 0.9 | 0.9 | 1.0 | 1.0 | 1.0 | 1.0 | 1.0 | 1.0 | 1.0 | 1.0 | 1.0 | 1.0 | 1.0 | 1.0 | 1.0 | 0.9 | 0.9 | 1.0 | 1.0 | 1.0 |
| SLF… | 1.0 | 1.0 | 1.0 | 1.0 | 1.0 | 1.0 | 0.9 | 0.9 | 0.9 | 1.0 | 1.0 | 1.0 | 1.0 | 1.0 | 1.0 | 1.0 | 1.0 | 1.0 | 1.0 | 1.0 | 1.0 | 0.9 | 0.9 | 0.9 | 1.0 | 1.0 | 1.0 |
| SYD… | 1.0 | 1.0 | 1.0 | 1.0 | 1.0 | 1.0 | 1.0 | 1.0 | 1.0 | 1.0 | 1.0 | 1.0 | 1.0 | 1.0 | 1.0 | 1.0 | 1.0 | 1.0 | 1.0 | 1.0 | 1.0 | 1.0 | 1.0 | 1.0 | 0.9 | 0.9 | 1.0 |
| SYD… | 1.0 | 1.0 | 1.0 | 1.0 | 1.0 | 1.0 | 1.0 | 1.0 | 1.0 | 1.0 | 1.0 | 1.0 | 1.0 | 1.0 | 1.0 | 1.0 | 1.0 | 1.0 | 1.0 | 1.0 | 1.0 | 1.0 | 1.0 | 1.0 | 0.9 | 0.9 | 1.0 |
| SYD… | 1.0 | 1.0 | 1.0 | 1.0 | 1.0 | 1.0 | 1.0 | 1.0 | 1.0 | 1.0 | 1.0 | 1.0 | 1.0 | 1.0 | 1.0 | 1.0 | 1.0 | 1.0 | 1.0 | 1.0 | 1.0 | 1.0 | 1.0 | 1.0 | 0.9 | 1.0 | 1.0 |
| TGV… | 1.0 | 1.0 | 1.0 | 1.0 | 1.0 | 1.0 | 1.0 | 1.0 | 1.0 | 1.0 | 1.0 | 1.0 | 1.0 | 1.0 | 1.0 | 0.9 | 1.0 | 0.9 | 1.0 | 1.0 | 1.0 | 1.0 | 1.0 | 1.0 | 0.9 | 0.9 | 0.9 |
| TGV… | 1.0 | 1.0 | 1.0 | 1.0 | 1.0 | 1.0 | 1.0 | 1.0 | 1.0 | 1.0 | 1.0 | 1.0 | 1.0 | 1.0 | 1.0 | 0.9 | 0.9 | 0.9 | 1.0 | 1.0 | 1.0 | 1.0 | 1.0 | 1.0 | 0.9 | 0.9 | 0.9 |
| TGV… | 1.0 | 1.0 | 1.0 | 1.0 | 1.0 | 1.0 | 1.0 | 1.0 | 1.0 | 1.0 | 1.0 | 1.0 | 1.0 | 1.0 | 1.0 | 0.9 | 0.9 | 0.9 | 1.0 | 1.0 | 1.0 | 1.0 | 1.0 | 1.0 | 0.9 | 0.9 | 0.9 |
| VAA… | 1.0 | 1.0 | 1.0 | 1.0 | 1.0 | 1.0 | 0.9 | 0.9 | 0.9 | 1.0 | 0.9 | 0.9 | 1.0 | 1.0 | 1.0 | 1.0 | 1.0 | 1.0 | 0.9 | 0.9 | 0.9 | 0.9 | 0.9 | 0.9 | 1.0 | 1.0 | 1.0 |
| VAA… | 1.0 | 1.0 | 1.0 | 1.0 | 1.0 | 1.0 | 0.9 | 0.9 | 0.9 | 1.0 | 0.9 | 0.9 | 1.0 | 1.0 | 1.0 | 1.0 | 1.0 | 1.0 | 0.9 | 0.9 | 1.0 | 0.9 | 0.9 | 0.9 | 1.0 | 1.0 | 1.0 |
| VAA… | 1.0 | 1.0 | 1.0 | 1.0 | 1.0 | 1.0 | 0.9 | 0.9 | 0.9 | 1.0 | 1.0 | 1.0 | 1.0 | 1.0 | 1.0 | 1.0 | 1.0 | 1.0 | 1.0 | 1.0 | 1.0 | 0.9 | 0.9 | 0.9 | 1.0 | 1.0 | 1.0 |

**(D)** OprN

| | OprN AAAQQAAIR | OprN AAAQQAAIR | OprN AAAQQAAIR | OprN ALPIGDPGELLR | OprN ALPIGDPGELLR | OprN ALPIGDPGELLR | OprN DAGVGAELDVLR | OprN DAGVGAELDVLR | OprN DAGVGAELDVLR | OprN IALSNLENQK | OprN IALSNLENQK | OprN IALSNLENQK | OprN IATLLGQRPEELTVDLSPR | OprN IATLLGQRPEELTVDLSPR | OprN IATLLGQRPEELTVDLSPR | OprN IDATASKPYDR | OprN IDATASKPYDR | OprN IDATASKPYDR |
|---|---|---|---|---|---|---|---|---|---|---|---|---|---|---|---|---|---|---|
| OprN AAAQQAAIR | 1.0 | 1.0 | 1.0 | 0.9 | 0.9 | 0.9 | 0.9 | 0.9 | 0.9 | 0.9 | 0.9 | 0.7 | 0.9 | 0.9 | 0.9 | 0.9 | 0.9 | 0.9 |
| OprN AAAQQAAIR | 1.0 | 1.0 | 1.0 | 0.9 | 0.9 | 0.9 | 0.9 | 0.9 | 0.9 | 0.9 | 0.9 | 0.7 | 0.9 | 0.9 | 0.9 | 0.9 | 0.9 | 0.9 |
| OprN AAAQQAAIR | 1.0 | 1.0 | 1.0 | 0.9 | 0.9 | 0.9 | 0.9 | 0.9 | 0.9 | 0.9 | 0.9 | 0.7 | 0.9 | 0.9 | 0.9 | 0.9 | 0.9 | 0.9 |
| OprN ALPIGDPGELLR | 0.9 | 0.9 | 0.9 | 1.0 | 1.0 | 1.0 | 1.0 | 1.0 | 1.0 | 1.0 | 1.0 | 0.6 | 1.0 | 1.0 | 1.0 | 1.0 | 1.0 | 1.0 |
| OprN ALPIGDPGELLR | 0.9 | 0.9 | 0.9 | 1.0 | 1.0 | 1.0 | 1.0 | 1.0 | 1.0 | 1.0 | 1.0 | 0.6 | 1.0 | 1.0 | 1.0 | 1.0 | 1.0 | 1.0 |
| OprN ALPIGDPGELLR | 0.9 | 0.9 | 0.9 | 1.0 | 1.0 | 1.0 | 1.0 | 1.0 | 1.0 | 1.0 | 1.0 | 0.6 | 1.0 | 1.0 | 1.0 | 1.0 | 1.0 | 1.0 |
| OprN DAGVGAELDVLR | 0.9 | 0.9 | 0.9 | 1.0 | 1.0 | 1.0 | 1.0 | 1.0 | 1.0 | 1.0 | 1.0 | 0.6 | 1.0 | 1.0 | 1.0 | 1.0 | 1.0 | 1.0 |
| OprN DAGVGAELDVLR | 0.9 | 0.9 | 0.9 | 1.0 | 1.0 | 1.0 | 1.0 | 1.0 | 1.0 | 1.0 | 1.0 | 0.6 | 1.0 | 1.0 | 1.0 | 1.0 | 1.0 | 1.0 |
| OprN DAGVGAELDVLR | 0.9 | 0.9 | 0.9 | 1.0 | 1.0 | 1.0 | 1.0 | 1.0 | 1.0 | 1.0 | 1.0 | 0.6 | 1.0 | 1.0 | 1.0 | 1.0 | 1.0 | 1.0 |
| OprN IALSNLENQK | 0.9 | 0.9 | 0.9 | 1.0 | 1.0 | 1.0 | 1.0 | 1.0 | 1.0 | 1.0 | 1.0 | 0.6 | 0.9 | 0.9 | 0.9 | 1.0 | 1.0 | 1.0 |
| OprN IALSNLENQK | 0.9 | 0.9 | 0.9 | 1.0 | 1.0 | 1.0 | 1.0 | 1.0 | 1.0 | 1.0 | 1.0 | 0.6 | 1.0 | 1.0 | 1.0 | 1.0 | 1.0 | 1.0 |
| OprN IALSNLENQK | 0.7 | 0.7 | 0.7 | 0.6 | 0.6 | 0.6 | 0.6 | 0.6 | 0.6 | 0.6 | 0.6 | 1.0 | 0.6 | 0.7 | 0.7 | 0.7 | 0.6 | 0.6 |
| OprN IATLLGQRPEELTVDLSPR | 0.9 | 0.9 | 0.9 | 1.0 | 1.0 | 1.0 | 1.0 | 1.0 | 1.0 | 0.9 | 1.0 | 0.6 | 1.0 | 1.0 | 1.0 | 1.0 | 1.0 | 1.0 |
| OprN IATLLGQRPEELTVDLSPR | 0.9 | 0.9 | 0.9 | 1.0 | 1.0 | 1.0 | 1.0 | 1.0 | 1.0 | 0.9 | 1.0 | 0.7 | 1.0 | 1.0 | 1.0 | 1.0 | 1.0 | 1.0 |
| OprN IATLLGQRPEELTVDLSPR | 0.9 | 0.9 | 0.9 | 1.0 | 1.0 | 1.0 | 1.0 | 1.0 | 1.0 | 0.9 | 1.0 | 0.7 | 1.0 | 1.0 | 1.0 | 1.0 | 1.0 | 1.0 |
| OprN IDATASKPYDR | 0.9 | 0.9 | 0.9 | 1.0 | 1.0 | 1.0 | 1.0 | 1.0 | 1.0 | 1.0 | 1.0 | 0.7 | 1.0 | 1.0 | 1.0 | 1.0 | 1.0 | 1.0 |
| OprN IDATASKPYDR | 0.9 | 0.9 | 0.9 | 1.0 | 1.0 | 1.0 | 1.0 | 1.0 | 1.0 | 1.0 | 1.0 | 0.6 | 1.0 | 1.0 | 1.0 | 1.0 | 1.0 | 1.0 |
| OprN IDATASKPYDR | 0.9 | 0.9 | 0.9 | 1.0 | 1.0 | 1.0 | 1.0 | 1.0 | 1.0 | 1.0 | 1.0 | 0.6 | 1.0 | 1.0 | 1.0 | 1.0 | 1.0 | 1.0 |

**(E)** MexF

| | MexF ASHGYVGTVNR | MexF ASHGYVGTVNR | MexF ASHGYVGTVNR | MexF GNQGYEELFK | MexF GNQGYEELFK | MexF GNQGYEELFK | MexF LEPEQIGQLK | MexF LEPEQIGQLK | MexF LEPEQIGQLK | MexF LVTEEEFENIIIR | MexF LVTEEEFENIIIR | MexF LVTEEEFENIIIR | MexF NLTATDVVNAIR | MexF NLTATDVVNAIR | MexF NLTATDVVNAIR | MexF VAAVLEACR | MexF VAAVLEACR | MexF VAAVLEACR | MexF NAILIVEFAK | MexF NAILIVEFAK | MexF NAILIVEFAK |
|---|---|---|---|---|---|---|---|---|---|---|---|---|---|---|---|---|---|---|---|---|---|
| MexF ASHGYVGTVNR | 1.0 | 1.0 | 1.0 | 0.9 | 0.8 | 0.8 | 0.9 | 0.9 | 0.9 | 1.0 | 1.0 | 1.0 | 1.0 | 1.0 | 1.0 | 0.9 | 0.9 | 0.9 | 0.8 | 0.8 | 0.8 |
| MexF ASHGYVGTVNR | 1.0 | 1.0 | 1.0 | 0.9 | 0.8 | 0.8 | 0.9 | 0.9 | 0.9 | 1.0 | 1.0 | 1.0 | 1.0 | 1.0 | 1.0 | 0.9 | 0.9 | 0.9 | 0.8 | 0.8 | 0.8 |
| MexF ASHGYVGTVNR | 1.0 | 1.0 | 1.0 | 0.8 | 0.8 | 0.8 | 0.9 | 0.9 | 0.9 | 1.0 | 1.0 | 1.0 | 1.0 | 1.0 | 1.0 | 0.9 | 0.9 | 0.9 | 0.8 | 0.8 | 0.8 |
| MexF GNQGYEELFK | 0.9 | 0.9 | 0.8 | 1.0 | 1.0 | 1.0 | 0.9 | 0.9 | 0.9 | 0.9 | 0.9 | 0.9 | 0.9 | 0.9 | 0.9 | 0.8 | 0.8 | 0.8 | 0.8 | 0.8 | 0.8 |
| MexF GNQGYEELFK | 0.8 | 0.8 | 0.8 | 1.0 | 1.0 | 1.0 | 0.9 | 0.9 | 0.9 | 0.9 | 0.9 | 0.8 | 0.9 | 0.9 | 0.9 | 0.8 | 0.8 | 0.8 | 0.7 | 0.8 | 0.8 |
| MexF GNQGYEELFK | 0.8 | 0.8 | 0.8 | 1.0 | 1.0 | 1.0 | 0.9 | 0.9 | 0.9 | 0.9 | 0.9 | 0.8 | 0.9 | 0.9 | 0.9 | 0.8 | 0.8 | 0.8 | 0.7 | 0.8 | 0.8 |
| MexF LEPEQIGQLK | 0.9 | 0.9 | 0.9 | 0.9 | 0.9 | 0.9 | 1.0 | 1.0 | 1.0 | 0.9 | 1.0 | 0.9 | 1.0 | 1.0 | 1.0 | 0.9 | 0.9 | 0.9 | 0.8 | 0.8 | 0.8 |
| MexF LEPEQIGQLK | 0.9 | 0.9 | 0.9 | 0.9 | 0.9 | 0.9 | 1.0 | 1.0 | 1.0 | 1.0 | 1.0 | 0.9 | 1.0 | 1.0 | 1.0 | 0.9 | 0.9 | 0.9 | 0.8 | 0.8 | 0.8 |
| MexF LEPEQIGQLK | 0.9 | 0.9 | 0.9 | 0.9 | 0.9 | 0.9 | 1.0 | 1.0 | 1.0 | 0.9 | 1.0 | 0.9 | 1.0 | 1.0 | 1.0 | 0.9 | 0.9 | 0.9 | 0.8 | 0.9 | 0.8 |
| MexF LVTEEEFENIIIR | 1.0 | 1.0 | 1.0 | 0.9 | 0.9 | 0.9 | 0.9 | 1.0 | 0.9 | 1.0 | 1.0 | 1.0 | 1.0 | 0.9 | 0.9 | 1.0 | 1.0 | 1.0 | 0.9 | 0.9 | 0.9 |
| MexF LVTEEEFENIIIR | 1.0 | 1.0 | 1.0 | 0.9 | 0.9 | 0.9 | 1.0 | 1.0 | 1.0 | 1.0 | 1.0 | 1.0 | 1.0 | 1.0 | 1.0 | 1.0 | 1.0 | 1.0 | 0.9 | 0.9 | 0.9 |
| MexF LVTEEEFENIIIR | 1.0 | 1.0 | 1.0 | 0.9 | 0.8 | 0.8 | 0.9 | 0.9 | 0.9 | 1.0 | 1.0 | 1.0 | 0.9 | 0.9 | 0.9 | 1.0 | 1.0 | 1.0 | 0.9 | 0.9 | 0.9 |
| MexF NLTATDVVNAIR | 1.0 | 1.0 | 1.0 | 0.9 | 0.9 | 0.9 | 1.0 | 1.0 | 1.0 | 1.0 | 1.0 | 0.9 | 1.0 | 1.0 | 1.0 | 0.9 | 0.9 | 0.9 | 0.8 | 0.8 | 0.8 |
| MexF NLTATDVVNAIR | 1.0 | 1.0 | 1.0 | 0.9 | 0.9 | 0.9 | 1.0 | 1.0 | 1.0 | 0.9 | 1.0 | 0.9 | 1.0 | 1.0 | 1.0 | 0.9 | 0.9 | 0.9 | 0.8 | 0.8 | 0.8 |
| MexF NLTATDVVNAIR | 1.0 | 1.0 | 1.0 | 0.9 | 0.9 | 0.9 | 1.0 | 1.0 | 1.0 | 0.9 | 1.0 | 0.9 | 1.0 | 1.0 | 1.0 | 0.9 | 0.9 | 0.9 | 0.8 | 0.8 | 0.8 |
| MexF VAAVLEACR | 0.9 | 0.9 | 0.9 | 0.8 | 0.8 | 0.8 | 0.9 | 0.9 | 0.9 | 1.0 | 1.0 | 1.0 | 0.9 | 0.9 | 0.9 | 1.0 | 1.0 | 1.0 | 0.9 | 0.9 | 0.9 |
| MexF VAAVLEACR | 0.9 | 0.9 | 0.9 | 0.8 | 0.8 | 0.8 | 0.9 | 0.9 | 0.9 | 1.0 | 1.0 | 1.0 | 0.9 | 0.9 | 0.9 | 1.0 | 1.0 | 1.0 | 0.9 | 0.9 | 0.9 |
| MexF VAAVLEACR | 0.9 | 0.9 | 0.9 | 0.8 | 0.8 | 0.8 | 0.9 | 0.9 | 0.9 | 1.0 | 1.0 | 1.0 | 0.9 | 0.9 | 0.9 | 1.0 | 1.0 | 1.0 | 0.9 | 0.9 | 0.9 |
| MexF NAILIVEFAK | 0.8 | 0.8 | 0.8 | 0.8 | 0.7 | 0.7 | 0.8 | 0.8 | 0.8 | 0.9 | 0.9 | 0.9 | 0.8 | 0.8 | 0.8 | 0.9 | 0.9 | 0.9 | 1.0 | 1.0 | 1.0 |
| MexF NAILIVEFAK | 0.8 | 0.8 | 0.8 | 0.8 | 0.8 | 0.8 | 0.8 | 0.8 | 0.9 | 0.9 | 0.9 | 0.9 | 0.8 | 0.8 | 0.8 | 0.9 | 0.9 | 0.9 | 1.0 | 1.0 | 1.0 |
| MexF NAILIVEFAK | 0.8 | 0.8 | 0.8 | 0.8 | 0.8 | 0.8 | 0.8 | 0.8 | 0.8 | 0.9 | 0.9 | 0.9 | 0.8 | 0.8 | 0.8 | 0.9 | 0.9 | 0.9 | 1.0 | 1.0 | 1.0 |

**(F)** MexE

| | MexE AAVAATQAQLDAAR | MexE AAVAATQAQLDAAR | MexE AAVAATQAQLDAAR | MexE ASNAISAELADAR | MexE ASNAISAELADAR | MexE ASNAISAELADAR | MexE DEAVGTDLGK | MexE DEAVGTDLGK | MexE DEAVGTDLGK | MexE FVLVLDGDNK | MexE FVLVLDGDNK | MexE FVLVLDGDNK | MexE LDFLDNQVNPR | MexE LDFLDNQVNPR | MexE LDFLDNQVNPR | MexE LEAPESVELRPR | MexE LEAPESVELRPR | MexE LEAPESVELRPR | MexE LEAQLQQAR | MexE LEAQLQQAR | MexE LEAQLQQAR | MexE VYAYFDADER | MexE VYAYFDADER | MexE VYAYFDADER |
|---|---|---|---|---|---|---|---|---|---|---|---|---|---|---|---|---|---|---|---|---|---|---|---|---|
| AAV… | 1.0 | 1.0 | 1.0 | 1.0 | 1.0 | 1.0 | 1.0 | 1.0 | 1.0 | 1.0 | 1.0 | 1.0 | 1.0 | 1.0 | 1.0 | 1.0 | 1.0 | 1.0 | 0.9 | 0.9 | 0.9 | 1.0 | 1.0 | 1.0 |
| AAV… | 1.0 | 1.0 | 1.0 | 1.0 | 1.0 | 1.0 | 0.9 | 0.9 | 0.9 | 1.0 | 1.0 | 1.0 | 1.0 | 1.0 | 1.0 | 1.0 | 1.0 | 1.0 | 0.9 | 0.9 | 0.9 | 1.0 | 1.0 | 1.0 |
| AAV… | 1.0 | 1.0 | 1.0 | 1.0 | 1.0 | 1.0 | 0.9 | 0.9 | 0.9 | 1.0 | 1.0 | 1.0 | 1.0 | 1.0 | 1.0 | 1.0 | 1.0 | 1.0 | 0.9 | 0.9 | 0.9 | 1.0 | 1.0 | 1.0 |
| ASN… | 1.0 | 1.0 | 1.0 | 1.0 | 1.0 | 1.0 | 0.9 | 0.9 | 0.9 | 1.0 | 1.0 | 1.0 | 1.0 | 1.0 | 1.0 | 1.0 | 1.0 | 1.0 | 0.9 | 0.9 | 0.9 | 1.0 | 1.0 | 1.0 |
| ASN… | 1.0 | 1.0 | 1.0 | 1.0 | 1.0 | 1.0 | 0.9 | 0.9 | 0.9 | 1.0 | 1.0 | 1.0 | 1.0 | 1.0 | 1.0 | 1.0 | 1.0 | 1.0 | 0.9 | 0.9 | 0.9 | 1.0 | 1.0 | 1.0 |
| ASN… | 1.0 | 1.0 | 1.0 | 1.0 | 1.0 | 1.0 | 0.9 | 0.9 | 0.9 | 1.0 | 1.0 | 1.0 | 1.0 | 1.0 | 1.0 | 1.0 | 1.0 | 1.0 | 0.9 | 0.9 | 0.9 | 1.0 | 1.0 | 1.0 |
| DEA… | 1.0 | 0.9 | 0.9 | 0.9 | 0.9 | 0.9 | 1.0 | 1.0 | 1.0 | 1.0 | 0.9 | 0.9 | 1.0 | 1.0 | 1.0 | 1.0 | 1.0 | 1.0 | 0.9 | 0.9 | 0.9 | 1.0 | 1.0 | 1.0 |
| DEA… | 1.0 | 0.9 | 0.9 | 0.9 | 0.9 | 0.9 | 1.0 | 1.0 | 1.0 | 1.0 | 0.9 | 0.9 | 1.0 | 1.0 | 1.0 | 1.0 | 1.0 | 1.0 | 0.9 | 0.9 | 0.9 | 1.0 | 1.0 | 1.0 |
| DEA… | 1.0 | 0.9 | 0.9 | 0.9 | 0.9 | 0.9 | 1.0 | 1.0 | 1.0 | 0.9 | 0.9 | 0.9 | 1.0 | 1.0 | 1.0 | 1.0 | 1.0 | 1.0 | 0.9 | 0.9 | 0.9 | 1.0 | 0.9 | 0.9 |
| FVL… | 1.0 | 1.0 | 1.0 | 1.0 | 1.0 | 1.0 | 1.0 | 1.0 | 0.9 | 1.0 | 1.0 | 1.0 | 1.0 | 1.0 | 1.0 | 1.0 | 1.0 | 1.0 | 0.9 | 0.9 | 0.9 | 1.0 | 1.0 | 1.0 |
| FVL… | 1.0 | 1.0 | 1.0 | 1.0 | 1.0 | 1.0 | 0.9 | 0.9 | 0.9 | 1.0 | 1.0 | 1.0 | 1.0 | 1.0 | 1.0 | 1.0 | 1.0 | 1.0 | 0.9 | 0.9 | 0.9 | 1.0 | 1.0 | 1.0 |
| FVL… | 1.0 | 1.0 | 1.0 | 1.0 | 1.0 | 1.0 | 0.9 | 0.9 | 0.9 | 1.0 | 1.0 | 1.0 | 1.0 | 1.0 | 1.0 | 1.0 | 1.0 | 1.0 | 0.9 | 0.9 | 0.9 | 1.0 | 1.0 | 1.0 |
| LDF… | 1.0 | 1.0 | 1.0 | 1.0 | 1.0 | 1.0 | 1.0 | 1.0 | 1.0 | 1.0 | 1.0 | 1.0 | 1.0 | 1.0 | 1.0 | 1.0 | 1.0 | 1.0 | 0.9 | 0.9 | 0.9 | 1.0 | 1.0 | 1.0 |
| LDF… | 1.0 | 1.0 | 1.0 | 1.0 | 1.0 | 1.0 | 1.0 | 1.0 | 1.0 | 1.0 | 1.0 | 1.0 | 1.0 | 1.0 | 1.0 | 1.0 | 1.0 | 1.0 | 0.9 | 0.9 | 0.9 | 1.0 | 1.0 | 1.0 |
| LDF… | 1.0 | 1.0 | 1.0 | 1.0 | 1.0 | 1.0 | 1.0 | 1.0 | 1.0 | 1.0 | 1.0 | 1.0 | 1.0 | 1.0 | 1.0 | 1.0 | 1.0 | 1.0 | 0.9 | 0.9 | 0.9 | 1.0 | 1.0 | 1.0 |
| LEAP… | 1.0 | 1.0 | 1.0 | 1.0 | 1.0 | 1.0 | 1.0 | 1.0 | 1.0 | 1.0 | 1.0 | 1.0 | 1.0 | 1.0 | 1.0 | 1.0 | 1.0 | 1.0 | 0.9 | 0.9 | 0.9 | 1.0 | 1.0 | 1.0 |
| LEAP… | 1.0 | 1.0 | 1.0 | 1.0 | 1.0 | 1.0 | 1.0 | 1.0 | 1.0 | 1.0 | 1.0 | 1.0 | 1.0 | 1.0 | 1.0 | 1.0 | 1.0 | 1.0 | 0.9 | 0.9 | 0.9 | 1.0 | 1.0 | 1.0 |
| LEAP… | 1.0 | 1.0 | 1.0 | 1.0 | 1.0 | 1.0 | 1.0 | 1.0 | 1.0 | 1.0 | 1.0 | 1.0 | 1.0 | 1.0 | 1.0 | 1.0 | 1.0 | 1.0 | 0.9 | 0.9 | 0.9 | 1.0 | 1.0 | 1.0 |
| LEAQ… | 0.9 | 0.9 | 0.9 | 0.9 | 0.9 | 0.9 | 0.9 | 0.9 | 0.9 | 0.9 | 0.9 | 0.9 | 0.9 | 0.9 | 0.9 | 0.9 | 0.9 | 0.9 | 1.0 | 1.0 | 1.0 | 0.9 | 0.9 | 0.9 |
| LEAQ… | 0.9 | 0.9 | 0.9 | 0.9 | 0.9 | 0.9 | 0.9 | 0.9 | 0.9 | 0.9 | 0.9 | 0.9 | 0.9 | 0.9 | 0.9 | 0.9 | 0.9 | 0.9 | 1.0 | 1.0 | 1.0 | 0.9 | 0.9 | 0.9 |
| LEAQ… | 0.9 | 0.9 | 0.9 | 0.9 | 0.9 | 0.9 | 0.9 | 0.9 | 0.9 | 0.9 | 0.9 | 0.9 | 0.9 | 0.9 | 0.9 | 0.9 | 0.9 | 0.9 | 1.0 | 1.0 | 1.0 | 0.9 | 0.9 | 0.9 |
| VYA… | 1.0 | 1.0 | 1.0 | 1.0 | 1.0 | 1.0 | 1.0 | 1.0 | 1.0 | 1.0 | 1.0 | 1.0 | 1.0 | 1.0 | 1.0 | 1.0 | 1.0 | 1.0 | 0.9 | 0.9 | 0.9 | 1.0 | 1.0 | 1.0 |
| VYA… | 1.0 | 1.0 | 1.0 | 1.0 | 1.0 | 1.0 | 1.0 | 1.0 | 0.9 | 1.0 | 1.0 | 1.0 | 1.0 | 1.0 | 1.0 | 1.0 | 1.0 | 1.0 | 0.9 | 0.9 | 0.9 | 1.0 | 1.0 | 1.0 |
| VYA… | 1.0 | 1.0 | 1.0 | 1.0 | 1.0 | 1.0 | 1.0 | 1.0 | 0.9 | 1.0 | 1.0 | 1.0 | 1.0 | 1.0 | 1.0 | 1.0 | 1.0 | 1.0 | 0.9 | 0.9 | 0.9 | 1.0 | 1.0 | 1.0 |

**(G)** MexX

| | MexX AVNPQAITVPR | MexX AVNPQAITVPR | MexX AVNPQAITVPR | MexX EYTEAQTDAR | MexX EYTEAQTDAR | MexX EYTEAQTDAR | MexX LGYATVTAPIDGR | MexX LGYATVTAPIDGR | MexX LGYATVTAPIDGR | MexX LYEEGQDVR | MexX LYEEGQDVR | MexX LYEEGQDVR |
|---|---|---|---|---|---|---|---|---|---|---|---|---|
| MexX AVNPQAITVPR | 1.0 | 1.0 | 1.0 | 0.6 | 0.6 | 0.6 | 0.9 | 0.9 | 0.9 | 0.6 | 0.6 | 0.6 |
| MexX AVNPQAITVPR | 1.0 | 1.0 | 1.0 | 0.7 | 0.7 | 0.7 | 0.9 | 1.0 | 0.9 | 0.7 | 0.7 | 0.6 |
| MexX AVNPQAITVPR | 1.0 | 1.0 | 1.0 | 0.6 | 0.7 | 0.6 | 0.9 | 0.9 | 0.9 | 0.6 | 0.6 | 0.6 |
| MexX EYTEAQTDAR | 0.6 | 0.7 | 0.6 | 1.0 | 1.0 | 1.0 | 0.8 | 0.8 | 0.8 | 0.9 | 0.9 | 1.0 |
| MexX EYTEAQTDAR | 0.6 | 0.7 | 0.7 | 1.0 | 1.0 | 1.0 | 0.8 | 0.8 | 0.8 | 0.9 | 0.9 | 1.0 |
| MexX EYTEAQTDAR | 0.6 | 0.7 | 0.6 | 1.0 | 1.0 | 1.0 | 0.8 | 0.8 | 0.8 | 0.9 | 0.9 | 1.0 |
| MexX LGYATVTAPIDGR | 0.9 | 0.9 | 0.9 | 0.8 | 0.8 | 0.8 | 1.0 | 1.0 | 1.0 | 0.8 | 0.8 | 0.8 |
| MexX LGYATVTAPIDGR | 0.9 | 1.0 | 0.9 | 0.8 | 0.8 | 0.8 | 1.0 | 1.0 | 1.0 | 0.8 | 0.8 | 0.8 |
| MexX LGYATVTAPIDGR | 0.9 | 0.9 | 0.9 | 0.8 | 0.8 | 0.8 | 1.0 | 1.0 | 1.0 | 0.8 | 0.8 | 0.8 |
| MexX LYEEGQDVR | 0.6 | 0.7 | 0.6 | 0.9 | 0.9 | 0.9 | 0.8 | 0.8 | 0.8 | 1.0 | 1.0 | 1.0 |
| MexX LYEEGQDVR | 0.6 | 0.7 | 0.6 | 0.9 | 0.9 | 0.9 | 0.8 | 0.8 | 0.8 | 1.0 | 1.0 | 1.0 |
| MexX LYEEGQDVR | 0.6 | 0.6 | 0.6 | 1.0 | 1.0 | 1.0 | 0.8 | 0.8 | 0.8 | 1.0 | 1.0 | 1.0 |

**(H)** MexY

| | MexY AAWTLGPPQLTR | MexY AAWTLGPPQLTR | MexY AAWTLGPPQLTR | MexY EASQHVGAIVER | MexY EASQHVGAIVER | MexY EASQHVGAIVER | MexY VGLITIIGLSAK | MexY VGLITIIGLSAK | MexY VGLITIIGLSAK | MexY VVEEAVTAIIER | MexY VVEEAVTAIIER | MexY VVEEAVTAIIER |
|---|---|---|---|---|---|---|---|---|---|---|---|---|
| MexY AAWTLGPPQLTR | 1.0 | 0.8 | 0.8 | 0.3 | 0.2 | 0.3 | 0.0 | 0.0 | 0.0 | 0.2 | 0.3 | 0.3 |
| MexY AAWTLGPPQLTR | 0.8 | 1.0 | 1.0 | 0.1 | 0.1 | 0.1 | -0.1 | -0.1 | -0.1 | 0.0 | 0.1 | 0.1 |
| MexY AAWTLGPPQLTR | 0.8 | 1.0 | 1.0 | 0.1 | 0.1 | 0.1 | 0.0 | -0.1 | 0.0 | 0.0 | 0.1 | 0.1 |
| MexY EASQHVGAIVER | 0.3 | 0.1 | 0.1 | 1.0 | 0.9 | 1.0 | 0.6 | 0.6 | 0.7 | 0.9 | 0.9 | 0.9 |
| MexY EASQHVGAIVER | 0.2 | 0.1 | 0.1 | 0.9 | 1.0 | 1.0 | 0.6 | 0.7 | 0.7 | 0.9 | 0.9 | 0.9 |
| MexY EASQHVGAIVER | 0.3 | 0.1 | 0.1 | 1.0 | 1.0 | 1.0 | 0.6 | 0.6 | 0.7 | 1.0 | 1.0 | 1.0 |
| MexY VGLITIIGLSAK | 0.0 | -0.1 | 0.0 | 0.6 | 0.6 | 0.6 | 1.0 | 1.0 | 1.0 | 0.7 | 0.6 | 0.6 |
| MexY VGLITIIGLSAK | 0.0 | -0.1 | -0.1 | 0.6 | 0.7 | 0.6 | 1.0 | 1.0 | 1.0 | 0.7 | 0.6 | 0.6 |
| MexY VGLITIIGLSAK | 0.0 | -0.1 | 0.0 | 0.7 | 0.7 | 0.7 | 1.0 | 1.0 | 1.0 | 0.8 | 0.7 | 0.7 |
| MexY VVEEAVTAIIER | 0.2 | 0.0 | 0.0 | 0.9 | 0.9 | 1.0 | 0.7 | 0.7 | 0.8 | 1.0 | 1.0 | 1.0 |
| MexY VVEEAVTAIIER | 0.3 | 0.1 | 0.1 | 0.9 | 0.9 | 1.0 | 0.6 | 0.6 | 0.7 | 1.0 | 1.0 | 1.0 |
| MexY VVEEAVTAIIER | 0.3 | 0.1 | 0.1 | 0.9 | 0.9 | 1.0 | 0.6 | 0.6 | 0.7 | 1.0 | 1.0 | 1.0 |

**(I)** AmpC Cephalosporinase

| | AmpC DLGLVILANR | AmpC DLGLVILANR | AmpC DLGLVILANR | AmpC FVDANLHPER | AmpC FVDANLHPER | AmpC FVDANLHPER | AmpC LPAPQALEGQR | AmpC LPAPQALEGQR | AmpC LPAPQALEGQR | AmpC SLGQPFER | AmpC SLGQPFER | AmpC SLGQPFER | AmpC TGSTNGFGAYVAFVPGR | AmpC TGSTNGFGAYVAFVPGR | AmpC TGSTNGFGAYVAFVPGR | AmpC TSAADLLR | AmpC TSAADLLR | AmpC TSAADLLR | AmpC VGPGPLDAEGYGVK | AmpC VGPGPLDAEGYGVK | AmpC VGPGPLDAEGYGVK |
|---|---|---|---|---|---|---|---|---|---|---|---|---|---|---|---|---|---|---|---|---|---|
| AmpC DLGLVILANR | 1.0 | 1.0 | 1.0 | 1.0 | 1.0 | 1.0 | 1.0 | 1.0 | 1.0 | 0.9 | 1.0 | 1.0 | 1.0 | 1.0 | 1.0 | 1.0 | 1.0 | 1.0 | 1.0 | 1.0 | 1.0 |
| AmpC DLGLVILANR | 1.0 | 1.0 | 1.0 | 1.0 | 1.0 | 1.0 | 1.0 | 1.0 | 1.0 | 0.9 | 1.0 | 1.0 | 1.0 | 1.0 | 1.0 | 1.0 | 1.0 | 1.0 | 1.0 | 1.0 | 1.0 |
| AmpC DLGLVILANR | 1.0 | 1.0 | 1.0 | 1.0 | 1.0 | 1.0 | 1.0 | 1.0 | 1.0 | 0.9 | 1.0 | 1.0 | 1.0 | 1.0 | 1.0 | 1.0 | 1.0 | 1.0 | 1.0 | 1.0 | 1.0 |
| AmpC FVDANLHPER | 1.0 | 1.0 | 1.0 | 1.0 | 1.0 | 1.0 | 1.0 | 1.0 | 1.0 | 0.9 | 1.0 | 1.0 | 1.0 | 0.9 | 1.0 | 1.0 | 1.0 | 1.0 | 1.0 | 1.0 | 1.0 |
| AmpC FVDANLHPER | 1.0 | 1.0 | 1.0 | 1.0 | 1.0 | 1.0 | 1.0 | 1.0 | 1.0 | 0.9 | 1.0 | 1.0 | 1.0 | 0.9 | 1.0 | 1.0 | 1.0 | 1.0 | 1.0 | 1.0 | 1.0 |
| AmpC FVDANLHPER | 1.0 | 1.0 | 1.0 | 1.0 | 1.0 | 1.0 | 1.0 | 1.0 | 1.0 | 0.9 | 1.0 | 1.0 | 0.9 | 0.9 | 0.9 | 1.0 | 1.0 | 1.0 | 1.0 | 1.0 | 1.0 |
| AmpC LPAPQALEGQR | 1.0 | 1.0 | 1.0 | 1.0 | 1.0 | 1.0 | 1.0 | 1.0 | 1.0 | 0.9 | 1.0 | 1.0 | 1.0 | 0.9 | 1.0 | 1.0 | 1.0 | 1.0 | 1.0 | 1.0 | 1.0 |
| AmpC LPAPQALEGQR | 1.0 | 1.0 | 1.0 | 1.0 | 1.0 | 1.0 | 1.0 | 1.0 | 1.0 | 0.9 | 1.0 | 1.0 | 1.0 | 1.0 | 1.0 | 1.0 | 1.0 | 1.0 | 1.0 | 1.0 | 1.0 |
| AmpC LPAPQALEGQR | 1.0 | 1.0 | 1.0 | 1.0 | 1.0 | 1.0 | 1.0 | 1.0 | 1.0 | 0.9 | 1.0 | 1.0 | 1.0 | 1.0 | 1.0 | 1.0 | 1.0 | 1.0 | 1.0 | 1.0 | 1.0 |
| AmpC SLGQPFER | 0.9 | 0.9 | 0.9 | 0.9 | 0.9 | 0.9 | 0.9 | 0.9 | 0.9 | 1.0 | 0.9 | 0.9 | 0.9 | 0.9 | 0.9 | 0.9 | 0.9 | 0.9 | 0.9 | 0.9 | 0.9 |
| AmpC SLGQPFER | 1.0 | 1.0 | 1.0 | 1.0 | 1.0 | 1.0 | 1.0 | 1.0 | 1.0 | 0.9 | 1.0 | 1.0 | 0.9 | 0.9 | 0.9 | 1.0 | 1.0 | 1.0 | 1.0 | 1.0 | 1.0 |
| AmpC SLGQPFER | 1.0 | 1.0 | 1.0 | 1.0 | 1.0 | 1.0 | 1.0 | 1.0 | 1.0 | 0.9 | 1.0 | 1.0 | 1.0 | 0.9 | 1.0 | 1.0 | 1.0 | 1.0 | 1.0 | 1.0 | 1.0 |
| AmpC TGSTNGFGAYVAFVPGR | 1.0 | 1.0 | 1.0 | 1.0 | 1.0 | 0.9 | 1.0 | 1.0 | 1.0 | 0.9 | 0.9 | 1.0 | 1.0 | 1.0 | 1.0 | 0.9 | 0.9 | 0.9 | 1.0 | 1.0 | 1.0 |
| AmpC TGSTNGFGAYVAFVPGR | 1.0 | 1.0 | 1.0 | 0.9 | 0.9 | 0.9 | 0.9 | 1.0 | 1.0 | 0.9 | 0.9 | 0.9 | 1.0 | 1.0 | 1.0 | 0.9 | 0.9 | 0.9 | 0.9 | 1.0 | 0.9 |
| AmpC TGSTNGFGAYVAFVPGR | 1.0 | 1.0 | 1.0 | 1.0 | 1.0 | 0.9 | 1.0 | 1.0 | 1.0 | 0.9 | 0.9 | 1.0 | 1.0 | 1.0 | 1.0 | 0.9 | 0.9 | 0.9 | 1.0 | 1.0 | 0.9 |
| AmpC TSAADLLR | 1.0 | 1.0 | 1.0 | 1.0 | 1.0 | 1.0 | 1.0 | 1.0 | 1.0 | 0.9 | 1.0 | 1.0 | 0.9 | 0.9 | 0.9 | 1.0 | 1.0 | 1.0 | 1.0 | 1.0 | 1.0 |
| AmpC TSAADLLR | 1.0 | 1.0 | 1.0 | 1.0 | 1.0 | 1.0 | 1.0 | 1.0 | 1.0 | 0.9 | 1.0 | 1.0 | 0.9 | 0.9 | 0.9 | 1.0 | 1.0 | 1.0 | 1.0 | 1.0 | 1.0 |
| AmpC TSAADLLR | 1.0 | 1.0 | 1.0 | 1.0 | 1.0 | 1.0 | 1.0 | 1.0 | 1.0 | 0.9 | 1.0 | 1.0 | 0.9 | 0.9 | 0.9 | 1.0 | 1.0 | 1.0 | 1.0 | 1.0 | 1.0 |
| AmpC VGPGPLDAEGYGVK | 1.0 | 1.0 | 1.0 | 1.0 | 1.0 | 1.0 | 1.0 | 1.0 | 1.0 | 0.9 | 1.0 | 1.0 | 1.0 | 0.9 | 1.0 | 1.0 | 1.0 | 1.0 | 1.0 | 1.0 | 1.0 |
| AmpC VGPGPLDAEGYGVK | 1.0 | 1.0 | 1.0 | 1.0 | 1.0 | 1.0 | 1.0 | 1.0 | 1.0 | 0.9 | 1.0 | 1.0 | 1.0 | 1.0 | 1.0 | 1.0 | 1.0 | 1.0 | 1.0 | 1.0 | 1.0 |
| AmpC VGPGPLDAEGYGVK | 1.0 | 1.0 | 1.0 | 1.0 | 1.0 | 1.0 | 1.0 | 1.0 | 1.0 | 0.9 | 1.0 | 1.0 | 1.0 | 0.9 | 0.9 | 1.0 | 1.0 | 1.0 | 1.0 | 1.0 | 1.0 |

**Supplementary Table 9. Location of putative proteotypic peptides in representative sequences of OprD protein and association of SRM profiles to representative sequences.**

| Peptide sequence | SRM method detection | amino acid position in representative sequences | | | | | | |
|---|---|---|---|---|---|---|---|---|
| | | P32722 | J7DBR3 | E2ZPX5 | G5FXJ2 | A3LMK1 | F8J2B1 | A6VA14 |
| ANADEGEGDQNEFR | detected | NA | NA | NA | 418-431 | NA | NA | NA |
| ANADQAEGDQNEFR | detected | NA | NA | 420-433 | NA | NA | NA | NA |
| ANADQGEGDQDEFR | excluded | NA | NA | NA | NA | NA | NA | 418-431 |
| ANADQGEGDQNEFR | detected | 420-433 | 420-433 | NA | NA | 418-431 | NA | NA |
| GELYATYAGETAK | excluded | 193-205 | 193-205 | 193-205 | NA | NA | 193-205 | NA |
| GELYATYAGQTAK | excluded | NA | NA | NA | 193-205 | 193-205 | NA | 193-205 |
| GFIEDSSLDLLLR | detected | 35-47 | 35-47 | 35-47 | 35-47 | NA | 35-47 | 35-47 |
| GFIEDSSLNLLLR | detected | NA | NA | NA | NA | 35-47 | NA | NA |
| HHETNFEAK | excluded | NA | NA | NA | NA | NA | NA | 388-396 |
| HHETNLEAK | not detected | 390-398 | 390-398 | 390-398 | 388-396 | 388-396 | 390-398 | NA |
| LDGTSDK | excluded | 96-102 | 96-102 | 96-102 | 96-102 | 96-102 | 96-102 | 96-102 |
| MSDNNVGYK | excluded | 372-380 | 372-380 | 372-380 | NA | NA | 372-380 | NA |
| NYGYGEDGK | excluded | 381-389 | 381-389 | 381-389 | NA | NA | 381-389 | NA |
| SADFAGGR | excluded | NA | NA | NA | 206-213 | 206-213 | NA | 206-213 |
| SADFIGGR | excluded | 206-213 | 206-213 | 206-213 | NA | NA | 206-213 | NA |
| SGTGNLPVMNDGTPR | not detected | NA | 103-117 | 103-117 | NA | NA | 103-117 | NA |
| TGTGNLPVMNDGKPR | not detected | 103-117 | NA | NA | 103-117 | 103-117 | NA | 103-117 |
| VDSSSAYAGLYGEDGK | excluded | NA | NA | NA | NA | NA | NA | 372-387 |
| VDSSSSYAGLYGEDGK | detected | NA | NA | NA | 372-387 | 372-387 | NA | NA |
| VHGDQPFDYIGFGR | detected | 297-310 | 297-310 | NA | NA | NA | NA | NA |
| WGEMQPTAPVFAAGGSR | excluded | 138-154 | 138-154 | 138-154 | 138-154 | 138-154 | 138-154 | 138-154 |
| YAITDNLSASLYGAELK | excluded | NA | NA | NA | 214-230 | 214-230 | NA | 214-230 |
| YDLNLASYGVPGLTFMLR | excluded | NA | NA | NA | NA | 343-360 | NA | NA |
| YDLNLASYGVPGLTFMVR | excluded | 343-360 | 343-360 | 343-360 | NA | NA | 343-360 | NA |
| YDLNMASYGVPGLTFMVR | excluded | NA | NA | NA | 343-360 | NA | NA | 343-360 |
| YVVQAGPAK | detected | NA | NA | NA | 397-405 | NA | NA | NA |
| YVVQSGPAK | detected | 399-407 | 399-407 | 399-407 | NA | 397-405 | 399-407 | 397-405 |
| | SRM profile | 1 | 1 | 2 | 3 | 4 | NA | NA |
| | Corrective factor | 1 | 1 | 0.742 | 1.099 | 0.496 | NA | NA |

**Supplementary Table 10. Protein and mRNA measurements of MexAB-OprM efflux system in the literature-based and clinical-based sets of strains.**

| Sample name | MexAB-OprM Status | Protein measurements[a] | | | | mRNA measurements[c] | | | |
|---|---|---|---|---|---|---|---|---|---|
| | | MexA | MexB | OprM | MexAB-OprM interpretation[b] | *mexA* | *mexB* | *oprM* | *mexAB-oprM* interpretation[d] |
| 113 | unknown | 0.20 | 1.05 | 0.72 | basal-level | 1.17 | 2.01 | 0.45 | basal-level |
| 124.1 | unknown | 0.88 | 0.96 | 0.83 | basal-level | 0.44 | 1.29 | 0.36 | basal-level |
| 124.2 | unknown | 0.89 | 0.92 | 0.90 | basal-level | 0.46 | 1.13 | 0.30 | basal-level |
| 138 | unknown | 0.81 | 0.84 | 0.84 | basal-level | 1.24 | 1.87 | 0.44 | basal-level |
| 188 | unknown | 0.86 | 0.93 | 0.88 | basal-level | 0.67 | 0.83 | 0.25 | basal-level |
| 504 | unknown | 0.84 | 0.84 | 0.53 | basal-level | 0.55 | 0.64 | 0.40 | basal-level |
| Pa-001 | unknown | **2.06** | **2.08** | 1.47 | overproduced | 0.94 | 2.03 | 0.75 | basal-level |
| Pa-002 | unknown | **1.93** | **1.97** | 1.22 | overproduced | 0.66 | 1.29 | 0.47 | basal-level |
| Pa-004 | unknown | **1.45** | 1.58 | 1.60 | basal-level | 0.97 | 1.26 | 0.34 | basal-level |
| Pa-005 | unknown | **2.00** | **2.17** | **2.00** | overproduced | 1.65 | **3.04** | 0.83 | basal-level |
| Pa-006 | unknown | **7.28** | **7.87** | **8.54** | overproduced | **2.67** | **5.99** | 2.41 | over-expressed |
| Pa-007 | unknown | **5.16** | **4.96** | **4.87** | overproduced | 1.58 | 2.04 | 0.56 | basal-level |
| Pa-008 | unknown | 1.24 | 1.24 | 1.13 | basal-level | **1.70** | 1.25 | 0.31 | basal-level |
| Pa-009 | unknown | 1.04 | 1.05 | 0.77 | basal-level | 1.08 | 1.70 | 0.50 | basal-level |
| Pa-010 | unknown | **3.80** | **3.55** | **2.74** | overproduced | 0.59 | 1.43 | 0.27 | basal-level |
| 1113 | over-expressed | **3.78** | **3.14** | **1.91** | overproduced | **2.98** | **2.70** | **4.79** | over-expressed |
| 1217 | over-expressed | **2.17** | **2.26** | **1.81** | overproduced | 1.27 | 1.30 | 1.37 | basal-level |
| 1237 | over-expressed | **2.22** | **2.07** | 1.39 | overproduced | **2.77** | **2.69** | 2.48 | over-expressed |
| 1250 | over-expressed | **2.39** | **2.30** | 1.69 | overproduced | **2.23** | **2.28** | 2.74 | over-expressed |
| 1562 | over-expressed | **2.30** | **2.59** | **2.30** | overproduced | 1.56 | 1.70 | 1.68 | basal-level |
| 1727 | over-expressed | **8.13** | **6.75** | **6.42** | overproduced | **7.65** | **9.95** | **11.58** | over-expressed |
| 1738 | over-expressed | **3.88** | **3.67** | **3.22** | overproduced | **3.06** | **2.91** | 3.89 | over-expressed |
| 2085 | over-expressed | **2.32** | **2.25** | **2.19** | overproduced | **1.79** | 1.88 | 1.68 | basal-level |
| 2151 | over-expressed | **2.44** | **2.20** | 1.65 | overproduced | 1.50 | 1.16 | 1.24 | basal-level |
| 2172 | over-expressed | **3.08** | **2.58** | 1.78 | overproduced | **2.37** | 1.79 | 1.92 | basal-level |
| 12 | over-expressed | **8.22** | **7.38** | **6.83** | overproduced | **4.50** | **4.26** | **4.68** | over-expressed |
| PT629 | over-expressed | **4.23** | **3.76** | **3.07** | overproduced | **5.01** | **4.16** | **4.70** | over-expressed |
| 40-1 | basal-level | 0.78 | 0.82 | 0.87 | basal-level | 1.55 | 1.59 | 2.72 | basal-level |
| 93-1 | basal-level | 0.72 | 0.72 | 0.68 | basal-level | 0.80 | 0.91 | 1.01 | basal-level |
| 3936 | basal-level | 0.65 | 0.61 | 0.47 | basal-level | 0.72 | 0.82 | 1.22 | basal-level |
| PT1105 | basal-level | 1.11 | 1.19 | 1.02 | basal-level | 0.76 | 0.83 | 0.75 | basal-level |
| PT1155 | basal-level | 0.94 | 0.92 | 0.75 | basal-level | 0.75 | 0.56 | 0.70 | basal-level |
| PT1196 | basal-level | 0.98 | 0.82 | 0.55 | basal-level | 0.63 | 0.50 | 0.79 | basal-level |
| 14 | basal-level | 0.95 | 0.90 | 1.07 | basal-level | 0.64 | 0.75 | 0.95 | basal-level |
| 615S | basal-level | 0.77 | 0.77 | 0.77 | basal-level | 0.67 | 0.69 | 0.87 | basal-level |
| 615R | basal-level | **1.34** | 1.40 | 1.33 | basal-level | 1.26 | 1.57 | 1.57 | basal-level |
| 2112S | basal-level | 0.98 | **1.76** | 1.75 | basal-level | **1.75** | **2.49** | **5.43** | over-expressed |
| 2112R | basal-level | 0.75 | 1.41 | **1.96** | basal-level | 0.80 | 1.41 | 3.61 | basal-level |
| PT149 | basal-level | 0.61 | 0.63 | 0.60 | basal-level | 0.60 | 0.42 | 0.58 | basal-level |
| PT364 | basal-level | 0.82 | 0.79 | 0.59 | basal-level | 1.15 | 0.72 | 1.11 | basal-level |
| PAO1 | basal-level | 1.00 | 1.00 | 1.00 | basal-level | 1.00 | 1.00 | 1.00 | basal-level |
| 302S | basal-level | 0.74 | 0.77 | 0.75 | basal-level | 1.20 | 0.91 | 1.00 | basal-level |
| 302R9N | basal-level | 0.73 | 0.76 | 0.85 | basal-level | 1.04 | 0.88 | 1.20 | basal-level |
| 302R11N | basal-level | 0.59 | 0.64 | 0.65 | basal-level | 0.59 | 0.62 | 0.73 | basal-level |

[a]Assessed by LC-ESI-SRM, ratio relative to PAO1 strain, bold values were statistically significantly different from basal-level (two-sample two-sided t-test, P<0.05). [b]MexAB-OprM interpretation was based on MexA and MexB protein levels, expression was considered “basal-level” when none or one out of two was significantly overproduced and “overproduced” when MexA and MexB were significantly overproduced. [c]Assessed by RT-qPCR, ratio relative to PAO1 strain, bold values were statistically significantly different from basal-level (two-sample two-sided t-test, P<0.05). [d]Expression was based on *mexA* and *mexB* mRNA levels, expression was considered “basal-level” when none or one out of two was significantly over-expressed and “over-expressed” when *mexA* and *mexB* were significantly overproduced.

**Supplementary Table 11. Protein and mRNA measurements of MexCD-OprJ efflux system in the literature-based and clinical-based sets of strains.**

| Sample name | MexCD-OprJ Status | Protein measurements[a] | | | | mRNA measurements[c] | | | |
|---|---|---|---|---|---|---|---|---|---|
| | | MexC | MexD | OprJ | MexCD-OprJ interpretation[b] | *mexC* | *mexD* | *oprJ* | *mexCD-oprJ* interpretation[d] |
| 113 | unknown | ND | ND | ND | basal-level | 0.61 | 1.55 | 0.68 | basal-level |
| 124.1 | unknown | ND | ND | ND | basal-level | 0.57 | 1.20 | 0.37 | basal-level |
| 124.2 | unknown | 1.00 | 1.00 | 1.00 | overproduced | **3.78** | **10.31** | **5.12** | over-expressed |
| 138 | unknown | ND | ND | ND | basal-level | 0.59 | 1.22 | 0.88 | basal-level |
| 188 | unknown | ND | ND | ND | basal-level | 0.53 | 1.33 | 0.72 | basal-level |
| 504 | unknown | ND | ND | ND | basal-level | 0.71 | 0.89 | 0.87 | basal-level |
| Pa-01 | unknown | ND | ND | ND | basal-level | 1.01 | 2.47 | 1.62 | basal-level |
| Pa-02 | unknown | ND | ND | ND | basal-level | 1.30 | 2.23 | 1.15 | basal-level |
| Pa-04 | unknown | ND | ND | ND | basal-level | 0.55 | 0.87 | 0.51 | basal-level |
| Pa-05 | unknown | 2.27 | 1.42 | 5.28 | overproduced | **7.88** | **78.67** | **23.05** | over-expressed |
| Pa-06 | unknown | ND | ND | ND | basal-level | 0.57 | 0.71 | 0.76 | basal-level |
| Pa-07 | unknown | ND | ND | ND | basal-level | 0.93 | **4.35** | 2.49 | basal-level |
| Pa-08 | unknown | ND | ND | ND | basal-level | 0.59 | **5.61** | 2.87 | basal-level |
| Pa-09 | unknown | 2.30 | 1.89 | 2.42 | overproduced | **8.30** | **49.56** | **33.44** | over-expressed |
| Pa-10 | unknown | ND | ND | ND | basal-level | 0.14 | 0.25 | 0.28 | basal-level |
| 40-1 | basal-level | ND | ND | ND | basal-level | 2.86 | 0.71 | 3.66 | basal-level |
| 93-1 | basal-level | ND | ND | ND | basal-level | 1.07 | 0.65 | 1.32 | basal-level |
| 1113 | basal-level | ND | ND | ND | basal-level | 2.07 | 2.26 | **5.70** | basal-level |
| 1217 | basal-level | ND | ND | ND | basal-level | 0.98 | 0.51 | 1.77 | basal-level |
| 1237 | basal-level | ND | ND | ND | basal-level | 2.13 | **4.43** | **9.74** | basal-level |
| 1250 | basal-level | ND | ND | ND | basal-level | **3.38** | 2.94 | **5.00** | basal-level |
| 1562 | basal-level | ND | ND | ND | basal-level | 0.70 | 0.24 | 0.77 | basal-level |
| 1727 | basal-level | ND | ND | ND | basal-level | 2.19 | 0.23 | 1.18 | basal-level |
| 1738 | basal-level | ND | ND | ND | basal-level | 1.90 | 0.77 | 2.25 | basal-level |
| 2085 | basal-level | ND | ND | ND | basal-level | 0.99 | 2.31 | 4.14 | basal-level |
| 2151 | basal-level | ND | ND | ND | basal-level | 0.48 | 0.91 | 3.23 | basal-level |
| 2172 | basal-level | ND | ND | ND | basal-level | 0.73 | 0.32 | 1.32 | basal-level |
| 3936 | basal-level | ND | ND | ND | basal-level | 1.67 | 1.36 | 2.05 | basal-level |
| 302S | basal-level | ND | ND | ND | basal-level | 0.99 | 1.05 | 1.90 | basal-level |
| PT1105 | basal-level | ND | ND | ND | basal-level | 0.94 | 1.24 | 1.06 | basal-level |
| PT1155 | basal-level | ND | ND | ND | basal-level | 0.61 | 0.43 | 0.83 | basal-level |
| PT1196 | basal-level | ND | ND | ND | basal-level | 0.60 | 0.22 | 0.60 | basal-level |
| 14 | basal-level | ND | ND | ND | basal-level | 0.71 | 1.03 | 1.73 | basal-level |
| 12 | basal-level | ND | ND | ND | basal-level | 0.68 | 0.88 | 2.79 | basal-level |

| | | | | | | | | | |
|---|---|---|---|---|---|---|---|---|---|
| 615S | basal-level | ND | ND | ND | basal-level | 1.10 | 1.07 | 1.79 | basal-level |
| 615R | basal-level | ND | ND | ND | basal-level | 0.74 | 0.70 | 1.33 | basal-level |
| 2112S | basal-level | ND | ND | ND | basal-level | 2.78 | 1.10 | 2.33 | basal-level |
| 2112R | basal-level | ND | ND | ND | basal-level | 1.79 | 1.77 | 1.77 | basal-level |
| PT149 | basal-level | ND | ND | ND | basal-level | 1.09 | 2.00 | 2.36 | basal-level |
| PT364 | basal-level | ND | ND | ND | basal-level | 1.39 | 1.86 | 3.91 | basal-level |
| PAO1 | basal-level | ND | ND | ND | basal-level | 1.00 | 1.00 | 1.00 | basal-level |
| PT629 | basal-level | ND | ND | ND | basal-level | 1.05 | 1.60 | 3.12 | basal-level |
| 302R9N | basal-level | ND | ND | ND | basal-level | 1.03 | 1.16 | 1.66 | basal-level |
| 302R11N | basal-level | ND | ND | ND | basal-level | 0.55 | 0.48 | 0.75 | basal-level |

[a]Assessed by LC-ESI-SRM, ratio relative to 124.2 strain. [b]Strains with basal-level expression did not have detectable MexC or MexD protein. MexCD-OprJ was considered overproduced when MexC and MexD were detectable. [c]Assessed by RT-qPCR, ratio relative to PAO1 strain, bold values were statistically significantly different from basal-level (two-sample two-sided t-test, P<0.05). [d]Expression was based on *mexC* and *mexD* mRNA levels, *mexCD-oprJ* was considered "basal-level" when none or one out of two was significantly over-expressed and "over-expressed" when *mexC* and *mexD* were significantly overproduced. ND for Not Detected.

## Supplementary Table 12. Protein and mRNA measurements of MexEF-OprN efflux system in the literature-based and clinical-based sets of strains.

| Sample name | MexEF-OprN Status | Protein measurements[a] | | | | mRNA measurements[c] | | | |
|---|---|---|---|---|---|---|---|---|---|
| | | MexE | MexF | OprN | MexEF-OpN interpretation[b] | *mexE* | *mexF* | *oprN* | MexEF-OprN interpretation[d] |
| 113 | unknown | ND | ND | ND | basal-level | 5.19 | **78.14** | 2.53 | basal-level |
| 124.1 | unknown | ND | ND | ND | basal-level | 2.42 | 7.01 | 1.31 | basal-level |
| 124.2 | unknown | ND | ND | ND | basal-level | 6.35 | 11.59 | 2.29 | basal-level |
| 138 | unknown | ND | ND | ND | basal-level | 2.14 | 3.70 | 1.07 | basal-level |
| 188 | unknown | ND | ND | ND | basal-level | 1.22 | 22.03 | 0.90 | basal-level |
| 504 | unknown | ND | ND | ND | basal-level | 2.14 | 2.93 | 1.39 | basal-level |
| Pa-001 | unknown | ND | ND | ND | basal-level | 1.38 | 2.09 | 1.58 | basal-level |
| Pa-002 | unknown | ND | ND | ND | basal-level | 2.46 | 3.50 | 1.37 | basal-level |
| Pa-004 | unknown | ND | ND | ND | basal-level | 2.67 | **43.95** | 1.57 | basal-level |
| Pa-005 | unknown | ND | ND | ND | basal-level | 12.05 | 13.59 | 4.72 | basal-level |
| Pa-006 | unknown | ND | ND | ND | basal-level | 2.38 | **50.89** | **13.51** | basal-level |
| Pa-007 | unknown | ND | ND | ND | basal-level | 1.90 | 3.99 | 6.70 | basal-level |
| Pa-008 | unknown | ND | ND | ND | basal-level | 14.30 | 17.25 | **13.95** | basal-level |
| Pa-009 | unknown | ND | ND | ND | basal-level | 3.85 | 6.37 | **7.57** | basal-level |
| Pa-010 | unknown | ND | ND | ND | basal-level | 0.74 | 1.32 | 0.48 | basal-level |
| 40-1 | over-expressed | **0.58** | **0.62** | **0.44** | over-expressed | **110.06** | **89.60** | **37.51** | over-expressed |
| 93-1 | over-expressed | **0.99** | **1.04** | **1.01** | over-expressed | **62.82** | **76.06** | **18.91** | over-expressed |
| 3936 | over-expressed | **1.46** | **1.31** | **1.42** | over-expressed | **105.91** | **99.51** | **20.39** | over-expressed |
| 615S | over-expressed | **0.86** | **0.78** | **0.75** | over-expressed | **105.65** | **112.52** | **27.57** | over-expressed |
| PT149 | over-expressed | **1.00** | **1.00** | **1.00** | over-expressed | **149.44** | **142.52** | **24.44** | over-expressed |
| 302R9N | over-expressed | **0.25** | **0.31** | **0.45** | over-expressed | **199.78** | **231.75** | **37.70** | over-expressed |
| 302R11N | over-expressed | **0.27** | **0.50** | **0.41** | over-expressed | **94.41** | **97.36** | **20.26** | over-expressed |
| 1113 | basal-level | ND | ND | ND | basal-level | 4.11 | 5.43 | 4.04 | basal-level |
| 1217 | basal-level | ND | ND | ND | basal-level | 1.52 | 2.43 | 2.49 | basal-level |
| 1237 | basal-level | ND | ND | ND | basal-level | 21.41 | 21.77 | **7.77** | basal-level |
| 1250 | basal-level | 0.03 | 0.04 | 0.05 | basal-level | 4.66 | 7.65 | 4.61 | basal-level |

| | | | | | | | | | |
|---|---|---|---|---|---|---|---|---|---|
| 1562 | basal-level | ND | ND | ND | basal-level | 1.08 | 1.57 | 1.12 | basal-level |
| 1727 | basal-level | ND | ND | ND | basal-level | 4.10 | 5.44 | 3.57 | basal-level |
| 1738 | basal-level | 0.03 | 0.04 | ND | basal-level | 22.25 | 23.31 | 6.69 | basal-level |
| 2085 | basal-level | ND | ND | ND | basal-level | 2.49 | 3.42 | 1.33 | basal-level |
| 2151 | basal-level | ND | ND | ND | basal-level | 4.29 | 4.62 | 2.15 | basal-level |
| 2172 | basal-level | 0.04 | 0.05 | 0.0132 | basal-level | 9.69 | 8.08 | 2.95 | basal-level |
| 302S | basal-level | ND | ND | ND | basal-level | 6.96 | 8.09 | 3.04 | basal-level |
| PT1105 | basal-level | ND | ND | ND | basal-level | 1.59 | 1.83 | 0.96 | basal-level |
| PT1155 | basal-level | ND | ND | ND | basal-level | 1.16 | 1.81 | 0.86 | basal-level |
| PT1196 | basal-level | ND | ND | ND | basal-level | 0.71 | 1.20 | 0.67 | basal-level |
| 14 | basal-level | ND | ND | ND | basal-level | 2.25 | 3.48 | 1.50 | basal-level |
| 12 | basal-level | ND | ND | ND | basal-level | 5.07 | 6.77 | 2.51 | basal-level |
| 615R | basal-level | 0.03 | 0.03 | 0.02 | basal-level | **33.29** | **37.93** | 5.98 | over-expressed |
| 2112S | basal-level | ND | ND | ND | basal-level | 3.93 | 6.03 | 3.96 | basal-level |
| 2112R | basal-level | ND | ND | ND | basal-level | 3.47 | 4.43 | 1.78 | basal-level |
| PT364 | basal-level | ND | ND | ND | basal-level | 1.57 | 2.06 | 2.42 | basal-level |
| PAO1 | basal-level | ND | ND | ND | basal-level | 1.00 | 1.00 | 1.00 | basal-level |
| PT629 | basal-level | ND | ND | ND | basal-level | 1.10 | 1.68 | 0.93 | basal-level |

[a]Assessed by LC-ESI-SRM, ratio relative to PT149 strain, bold values were statistically significantly different from basal-level (two-sample two-sided t-test, P<0.05). [b]MexEF-OprN interpretation was based on MexE and MexF protein levels, MexEF-OprN was considered "basal-level" when none or one out of two was significantly overproduced and "overproduced" when MexE and MexF were significantly overproduced. [c]Assessed by RT-qPCR, ratio relative to PAO1 strain, bold values were statistically significantly different from basal-level (two-sample two-sided t-test, P<0.05). [d]*mexEF-oprN* interpretation was based on *mexC* and *mexD* mRNA levels, *mexEF-oprN* was considered "basal-level" when none or one out of two was significantly over-expressed and "over-expressed" when *mexC* and *mexD* were significantly overproduced. ND for Not Detected.

## Supplementary Table 13. Protein and mRNA measurements of MexXY(-OprM) efflux system in the literature-based and clinical-based sets of strains.

| Sample name | MexXY Status | Protein measurements[a] | | | | mRNA measurements[c] | | | |
|---|---|---|---|---|---|---|---|---|---|
| | | MexX | MexY | OprM | MexXY interpretation[b] | *mexX* | *mexY* | *oprM* | MexXY interpretation[d] |
| 113 | unknown | 0.47 | 0.71 | 0.72 | basal-level | 0.80 | 1.20 | 0.45 | basal-level |
| 124.1 | unknown | **21.16** | **18.11** | 0.83 | overproduced | **14.31** | **67.34** | 0.36 | over-expressed |
| 124.2 | unknown | 1.00 | 1.00 | 0.90 | basal-level | 2.23 | **5.07** | 0.30 | basal-level |
| 138 | unknown | 0.50 | 0.96 | 0.84 | basal-level | 1.28 | 1.63 | 0.44 | basal-level |
| 188 | unknown | 0.60 | 1.08 | 0.88 | basal-level | 0.70 | 1.07 | 0.25 | basal-level |
| 504 | unknown | 0.80 | 0.72 | 0.53 | basal-level | 1.35 | 2.10 | 0.40 | basal-level |
| Pa-001 | unknown | **11.17** | **10.00** | 1.47 | overproduced | **4.58** | **12.88** | 0.75 | over-expressed |
| Pa-002 | unknown | **15.04** | **12.10** | 1.22 | overproduced | **5.83** | **23.44** | 0.47 | over-expressed |
| Pa-004 | unknown | **15.75** | **13.61** | 1.60 | overproduced | **10.42** | **30.53** | 0.34 | over-expressed |
| Pa-005 | unknown | **13.83** | **9.70** | 2.00 | overproduced | **14.73** | **38.01** | 0.83 | over-expressed |
| Pa-006 | unknown | **19.84** | **15.31** | **8.54** | overproduced | **11.09** | **25.28** | 2.41 | over-expressed |
| Pa-007 | unknown | **14.10** | **8.61** | **4.87** | overproduced | 2.15 | **10.30** | 0.56 | basal-level |
| Pa-008 | unknown | **13.87** | **11.68** | 1.13 | overproduced | **9.31** | **54.11** | 0.31 | over-expressed |
| Pa-009 | unknown | **6.56** | **7.20** | 0.77 | overproduced | **4.62** | **13.06** | 0.50 | over-expressed |
| Pa-010 | unknown | **7.53** | **6.27** | **2.74** | overproduced | 3.11 | **6.04** | 0.27 | basal-level |
| 1113 | over-expressed | **16.30** | **12.32** | 1.91 | overproduced | **34.06** | **88.64** | 1.37 | over-expressed |

| | | | | | | | | | |
|---|---|---|---|---|---|---|---|---|---|
| 1217 | over-expressed | **28.06** | **19.63** | 1.81 | overproduced | **45.77** | **95.30** | 2.48 | over-expressed |
| 1237 | over-expressed | **14.67** | **10.07** | 1.39 | overproduced | **32.68** | **57.70** | 2.74 | over-expressed |
| 1250 | over-expressed | **14.82** | **11.10** | 1.69 | overproduced | **31.59** | **69.89** | 1.68 | over-expressed |
| 1562 | over-expressed | **25.18** | **17.85** | 2.30 | overproduced | **27.08** | **74.97** | **11.58** | over-expressed |
| 1727 | over-expressed | **21.65** | **17.93** | **6.42** | overproduced | **39.66** | **91.04** | **3.89** | over-expressed |
| 1738 | over-expressed | **18.86** | **13.79** | **3.22** | overproduced | **28.81** | **63.57** | 1.68 | over-expressed |
| 2085 | over-expressed | **18.36** | **13.30** | 2.19 | overproduced | **31.68** | **52.33** | 1.24 | over-expressed |
| 2151 | over-expressed | **18.32** | **13.80** | 1.65 | overproduced | **16.18** | **28.09** | 1.92 | over-expressed |
| 2172 | over-expressed | **12.07** | **8.83** | 1.78 | overproduced | **14.36** | **43.13** | 0.79 | over-expressed |
| PT1196 | over-expressed | **18.30** | **13.00** | 0.55 | overproduced | **27.84** | **85.11** | 0.95 | over-expressed |
| 14 | over-expressed | **18.82** | **15.41** | 1.07 | overproduced | **36.66** | **71.95** | **4.68** | over-expressed |
| 12 | over-expressed | **17.17** | **13.63** | **6.83** | overproduced | **17.22** | **40.18** | 1.57 | over-expressed |
| 615R | over-expressed | **14.00** | **12.47** | 1.33 | overproduced | **38.91** | **69.91** | **5.43** | over-expressed |
| 2112R | over-expressed | **12.58** | **11.22** | 1.96 | overproduced | **29.05** | **62.36** | 3.61 | over-expressed |
| 3936 | basal-level | 0.45 | 0.43 | 0.47 | basal-level | 1.52 | 1.36 | 1.22 | basal-level |
| 40-1 | basal-level | 0.99 | 1.09 | 0.87 | basal-level | 2.51 | 1.67 | 2.72 | basal-level |
| 93-1 | basal-level | 0.96 | 0.69 | 0.68 | basal-level | 1.53 | 1.63 | 1.01 | basal-level |
| 302S | basal-level | 0.64 | 0.85 | 0.75 | basal-level | 4.02 | 2.99 | 1.00 | basal-level |
| PT1105 | basal-level | 0.59 | 0.83 | 1.02 | basal-level | 1.41 | 1.36 | 0.75 | basal-level |
| PT1155 | basal-level | 0.45 | 0.62 | 0.75 | basal-level | 1.33 | 1.04 | 0.70 | basal-level |
| 615S | basal-level | **1.93** | 0.81 | 0.77 | basal-level | 0.83 | 0.64 | 0.87 | basal-level |
| 2112S | basal-level | 1.26 | **1.45** | 1.75 | basal-level | **34.07** | **67.69** | **4.79** | over-expressed |
| PT149 | basal-level | 0.83 | 0.72 | 0.60 | basal-level | 0.89 | 1.09 | 0.58 | basal-level |
| PT364 | basal-level | 0.49 | 0.54 | 0.59 | basal-level | 3.20 | 2.80 | 1.11 | basal-level |
| PAO1 | basal-level | 0.60 | 1.06 | 1.00 | basal-level | 1.00 | 1.00 | 1.00 | basal-level |
| PT629 | basal-level | 0.67 | 0.57 | **3.07** | basal-level | 2.17 | 2.32 | **4.70** | basal-level |
| 302R9N | basal-level | 0.64 | 0.81 | 0.85 | basal-level | 3.70 | 3.09 | 1.20 | basal-level |
| 302R11N | basal-level | 0.47 | 0.63 | 0.65 | basal-level | 2.23 | 1.82 | 0.73 | basal-level |

[a]Assessed by LC-ESI-SRM, ratio relative to 124.2 strain, bold values were statistically significantly different from basal-level (two-sample two-sided t-test, P<0.05). [b]MexXY interpretation was based on MexX and MexY protein levels, MexXY was considered "basal-level" when none or one out of two was significantly overproduced and "overproduced" when MexX and MexY were significantly overproduced. [c]Assessed by RT-qPCR, ratio relative to PAO1 strain, bold values were statistically significantly different from basal-level (two-sample two-sided t-test, P<0.05). [d]*mexXY* interpretation was based on *mexX* and *mexY* mRNA levels, *mexXY* was considered "basal-level" when none or one out of two was significantly over-expressed and "over-expressed" when *mexX* and *mexY* were significantly overproduced. ND for Not Detected.

**Supplementary Table 14. Protein and mRNA measurements of AmpC cephalosporinase in the literature-based and clinical-based sets of strains.**

| Sample | Protein measurements[a] | mRNA measurements[b] |
|---|---|---|

| name | AmpC | AmpC interpretation | *ampC* | AmpC interpretation |
|---|---|---|---|---|
| 40-1 | 1.27 | basal-level | 3.19 | basal-level |
| 93-1 | **66.94** | **derepressed** | **30.94** | **derepressed** |
| 113 | 0.27 | basal-level | 10.17 | basal-level |
| 124.1 | 1.45 | basal-level | 5.38 | basal-level |
| 124.2 | 1.13 | basal-level | 5.24 | basal-level |
| 138 | 0.52 | basal-level | 3.03 | basal-level |
| 188 | 0.73 | basal-level | 1.76 | basal-level |
| 504 | 0.64 | basal-level | 4.90 | basal-level |
| 1113 | 0.63 | basal-level | 2.18 | basal-level |
| 1217 | 0.68 | basal-level | 1.84 | basal-level |
| 1237 | 0.96 | basal-level | **30.03** | **derepressed** |
| 1250 | 2.61 | basal-level | 1.40 | basal-level |
| 1562 | 0.48 | basal-level | 0.90 | basal-level |
| 1727 | 0.60 | basal-level | 1.31 | basal-level |
| 1738 | 0.61 | basal-level | 1.45 | basal-level |
| 2085 | 0.52 | basal-level | 1.79 | basal-level |
| 2151 | 0.53 | basal-level | 1.90 | basal-level |
| 2172 | 0.39 | basal-level | 1.82 | basal-level |
| 3936 | 0.59 | basal-level | 1.19 | basal-level |
| Pa-001 | 1.21 | basal-level | **26.83** | **derepressed** |
| Pa-002 | **6.81** | **derepressed** | **25.96** | **derepressed** |
| Pa-004 | 0.53 | basal-level | 2.67 | basal-level |
| Pa-005 | 0.71 | basal-level | 7.01 | basal-level |
| Pa-006 | 0.59 | basal-level | 3.01 | basal-level |
| Pa-007 | **4.84** | **derepressed** | **25.98** | **derepressed** |
| Pa-008 | **23.05** | **derepressed** | **174.93** | **derepressed** |
| Pa-009 | **353.29** | **derepressed** | **847.65** | **derepressed** |
| Pa-010 | 0.94 | basal-level | 1.93 | basal-level |
| 302S | 0.90 | basal-level | 1.72 | basal-level |
| PT1105 | 0.95 | basal-level | 0.89 | basal-level |
| PT1155 | **10.47** | **derepressed** | 5.76 | basal-level |
| PT1196 | 0.55 | basal-level | 0.71 | basal-level |
| 14 | 0.52 | basal-level | 0.71 | basal-level |
| 12 | 0.52 | basal-level | 1.22 | basal-level |
| 615S | 0.51 | basal-level | 1.57 | basal-level |
| 615R | 0.33 | basal-level | 0.94 | basal-level |
| 2112S | **3.85** | **derepressed** | **17.82** | **derepressed** |
| 2112R | **94.30** | **derepressed** | **394.91** | **derepressed** |
| PT149 | 0.80 | basal-level | 0.65 | basal-level |
| PT364 | 0.63 | basal-level | 1.06 | basal-level |
| PAO1 | 1.00 | basal-level | 1.00 | basal-level |
| PT629 | 0.71 | basal-level | 1.52 | basal-level |
| 302R9N | 1.04 | basal-level | 1.85 | basal-level |
| 302R11N | 0.65 | basal-level | 1.40 | basal-level |

[a]Assessed by LC-ESI-SRM, ratio relative to PAO1 strain, bold values were at least three times higher produced than those of PAO1. [b]Assessed by RT-qPCR, ratio relative to PAO1 strain, bold values were at least fifteen times higher expressed than those of PAO1.

**Supplementary Table 15. Protein and mRNA measurements of porin OprD in the literature-based and clinical-based sets of strains.**

| Sample name | Protein measurements[a] | | mRNA measurements[b] | |
|---|---|---|---|---|
| | OprD | OprD interpretation | *oprD* | OprD interpretation |
| 40-1 | 0.28 | basal-level | 2.33 | basal-level |
| 93-1 | 0.14 | basal-level | 0.98 | basal-level |
| 113 | 0.72 | basal-level | 0.51 | basal-level |
| 124.1 | 0.84 | basal-level | 0.68 | basal-level |
| 124.2 | 0.68 | basal-level | 0.40 | basal-level |
| 138 | 0.61 | basal-level | 3.42 | basal-level |
| 188 | 1.10 | basal-level | 2.03 | basal-level |
| 504 | **0.01** | **repressed** | 4.92 | basal-level |
| 1113 | 0.38 | basal-level | 7.81 | basal-level |
| 1217 | **0.03** | **repressed** | **0.07** | **repressed** |
| 1237 | **0.01** | **repressed** | 0.69 | basal-level |
| 1250 | 0.51 | basal-level | 4.72 | basal-level |
| 1738 | 0.36 | basal-level | 2.10 | basal-level |
| 2085 | 0.21 | basal-level | 1.24 | basal-level |
| 2151 | 0.70 | basal-level | 1.99 | basal-level |
| 2172 | **0.01** | **repressed** | **0.13** | **repressed** |
| 3936 | 0.11 | basal-level | 0.93 | basal-level |
| Pa-001 | **ND** | **repressed** | **0.03** | **repressed** |
| Pa-002 | **ND** | **repressed** | **0.06** | **repressed** |
| Pa-004 | 0.55 | basal-level | 3.32 | basal-level |
| Pa-005 | 0.29 | basal-level | 3.79 | basal-level |
| Pa-006 | **0.01** | **repressed** | **0.14** | **repressed** |
| Pa-007 | 0.08 | basal-level | 1.07 | basal-level |
| Pa-008 | **ND** | **repressed** | **0.08** | **repressed** |
| Pa-009 | **ND** | **repressed** | **0.12** | **repressed** |
| Pa-010 | **0.02** | **repressed** | **0.17** | **repressed** |
| 302S | 0.67 | basal-level | 1.15 | basal-level |
| PT1105 | **0.02** | **repressed** | **0.05** | **repressed** |
| PT1155 | 0.77 | basal-level | 0.71 | basal-level |
| PT1196 | 0.58 | basal-level | 0.70 | basal-level |
| 14 | 0.66 | basal-level | 2.50 | basal-level |
| 12 | 0.39 | basal-level | 2.70 | basal-level |
| 615S | 0.11 | basal-level | 1.01 | basal-level |
| 615R | 0.19 | basal-level | 1.13 | basal-level |
| 2112S | **ND** | **repressed** | 0.89 | basal-level |
| 2112R | **0.03** | **repressed** | 0.34 | basal-level |
| PT149 | 0.28 | basal-level | 0.23 | basal-level |
| PT364 | **0.00** | **repressed** | **0.03** | **repressed** |
| PAO1 | 1.00 | basal-level | 1.00 | basal-level |
| PT629 | 0.60 | basal-level | 1.62 | basal-level |
| 302R9N | 0.62 | basal-level | 0.90 | basal-level |

[a]Assessed by LC-ESI-SRM, ratio relative to PAO1 strain, bold values were at least twenty times lower produced than those of PAO1. [b]Assessed by RT-qPCR, ratio relative to PAO1 strain, bold values were at least

five times lower expressed than those of PAO1.

## 1.2. Supplementary Figures

**Supplementary Figure 1. Correlation between MexA and MexB ratios measured by LC-ESI-SRM in strains of literature-based set.**

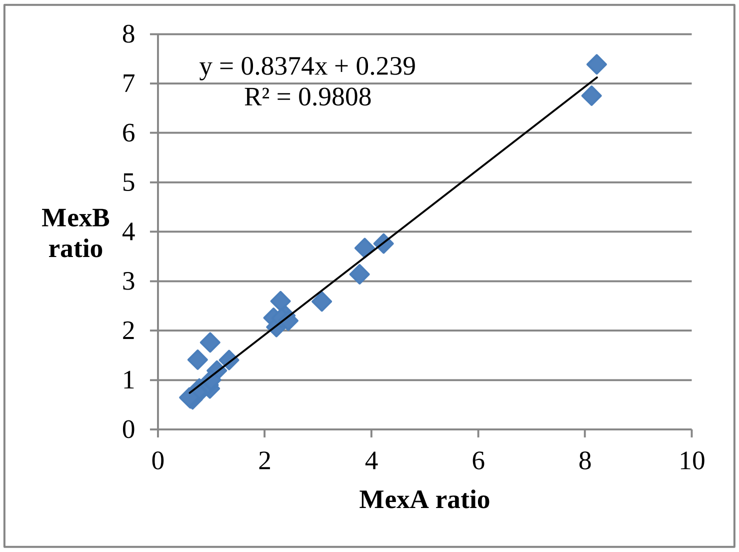


**Supplementary Figure 2. Correlation between *mexA* and *mexB* ratios measured by RT-qPCR in strains of literature-based set.**

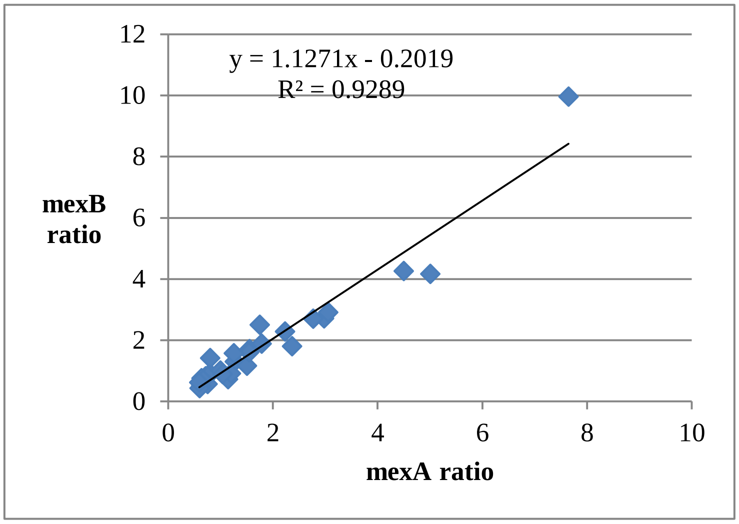

**Supplementary Figure 3. Correlation between *mexA* and MexA ratios measured by RT-qPCR and LC-ESI-SRM, respectively in strains of literature-based set.**

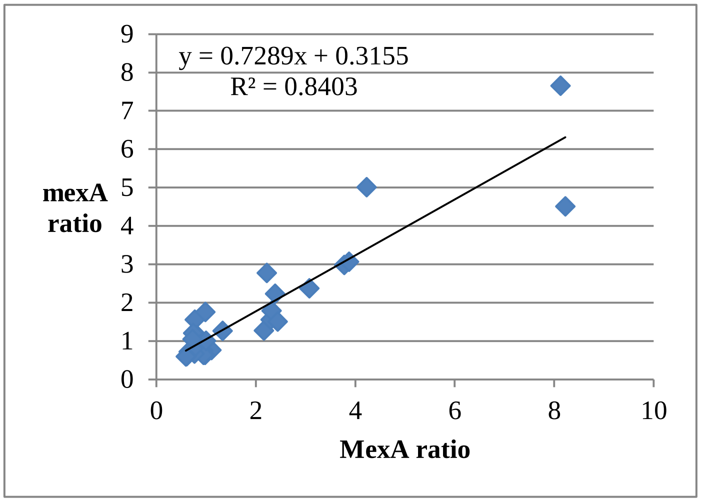


**Supplementary Figure 4. Correlation between MexE and MexF ratios measured by LC-ESI-SRM in strains of literature-based set.**

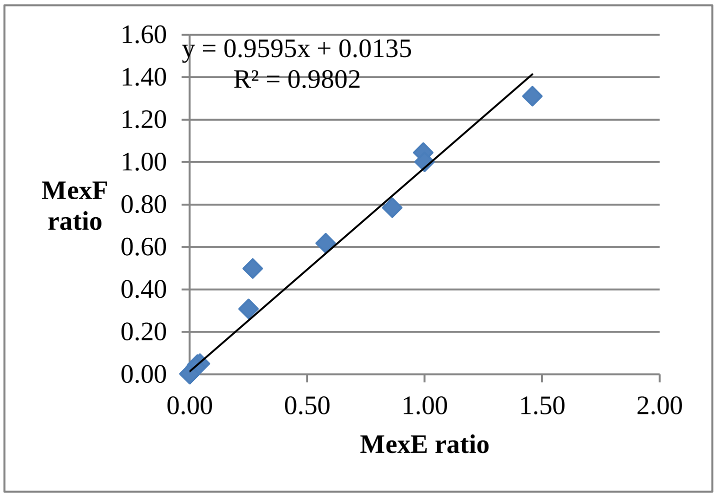

**Supplementary Figure 5. Correlation between *mexE* and *mexF* ratios measured by RT-qPCR in strains of literature-based set.**

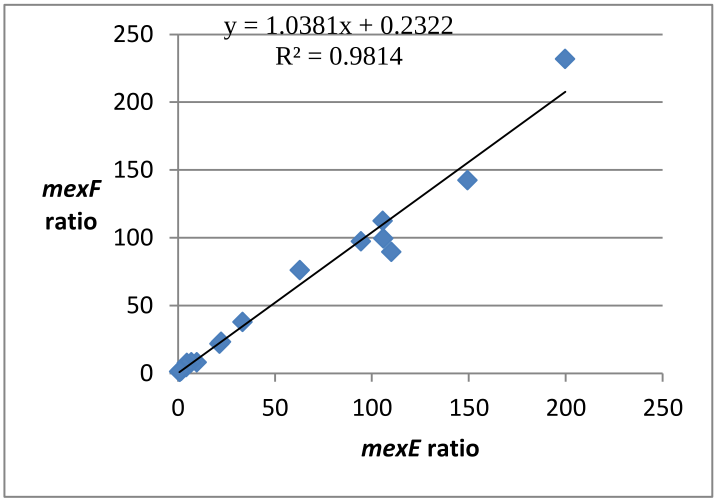


**Supplementary Figure 6. Correlation between MexX and MexY ratios measured by LC-ESI-SRM in strains of literature-based set.**

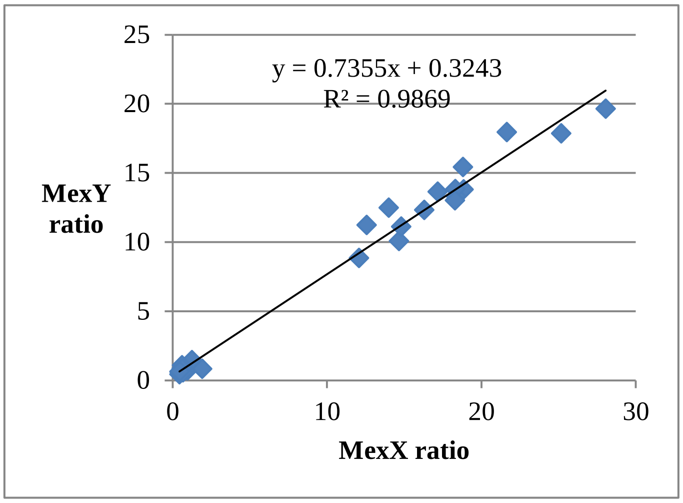

**Supplementary Figure 7. Correlation between *mexX* and *mexY* ratios measured by RT-qPCR in strains of literature-based set.**

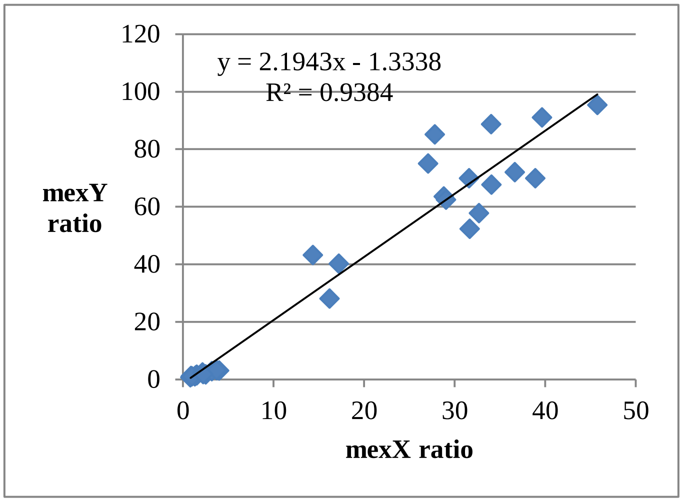


**Supplementary Figure 8. Correlation between MexX and OprM ratios measured by LC-ESI-SRM in strains of literature-based set.**

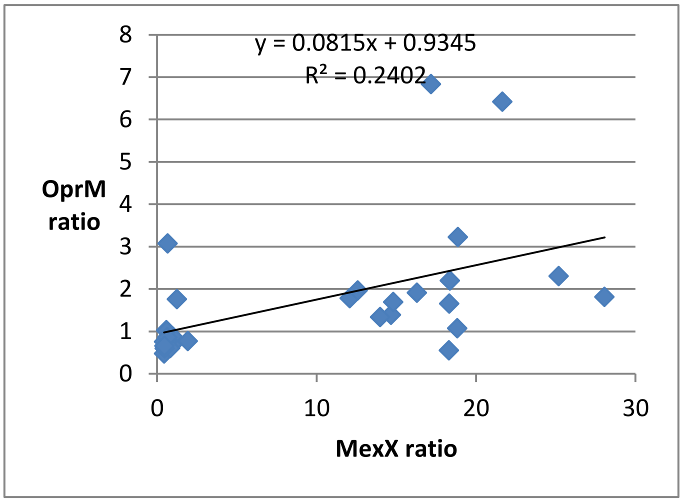

**Supplementary Figure 9. Correlation between MexY and OprM ratios measured by LC-ESI-SRM in strains of literature-based set.**

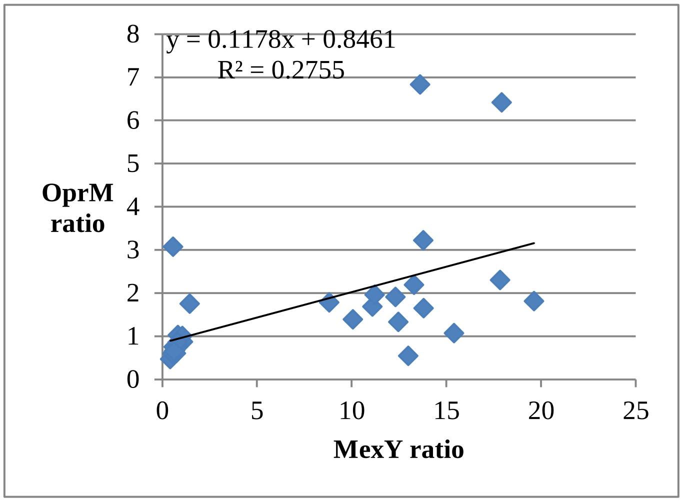


**Supplementary Figure 10. Correlation between MexA and MexB ratios measured by LC-ESI-SRM in strains of clinical-based set.**

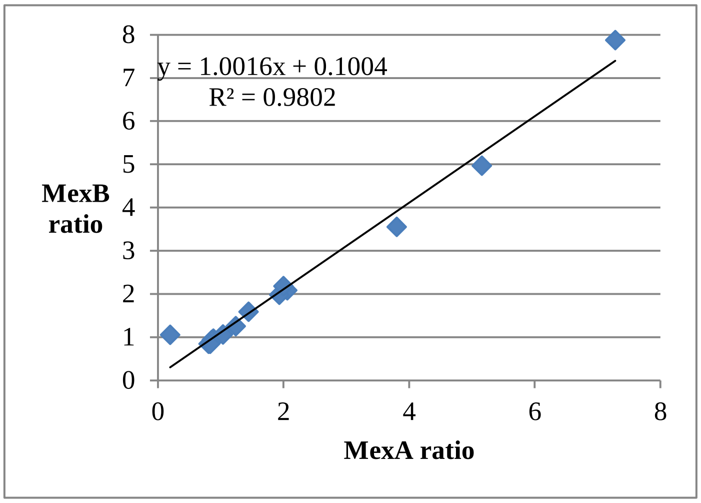

**Supplementary Figure 11. Correlation between *mexA* and *mexB* ratios measured by RT-qPCR in strains of clinical-based set.**

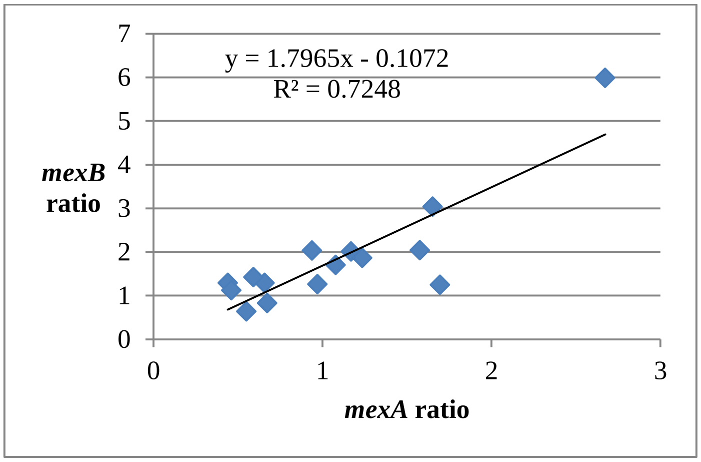


## 2. References

REFERENCES


Dumas, J. L., van Delden, C., Perron, K., and Köhler, T. (2006). Analysis of antibiotic resistance gene expression in *Pseudomonas aeruginosa* by quantitative real-time-PCR. *FEMS Microbiol. Lett.* 254, 217-225.

Llanes, C., Hocquet, D., Vogne, C., Benali-Baitich, D., Neuwirth, C., and Plesiat, P. (2004). Clinical strains of *Pseudomonas aeruginosa* overproducing MexAB-OprM and MexXY efflux pumps simultaneously. *Antimicrob. Agents Chemother.* 48, 1797-1802.

Llanes, C., Köhler, T., Patry, I., Dehecq, B., van Delden, C., and Plesiat, P. (2011). Role of the MexEF-OprN efflux system in low-level resistance of *Pseudomonas aeruginosa* to ciprofloxacin. *Antimicrob. Agents Chemother.* 55, 5676-5684.

Llanes, C., Pourcel, C., Richardot, C., Plesiat, P., Fichant, G., Cavallo, J. D. et al. (2013). Diversity of beta-lactam resistance mechanisms in cystic fibrosis isolates of *Pseudomonas aeruginosa*: a French multicentre study. *J. Antimicrob. Chemother.* 68, 1763-1771.

Vogne, C., Aires, J. R., Bailly, C., Hocquet, D., and Plesiat, P. (2004). Role of the multidrug efflux system MexXY in the emergence of moderate resistance to aminoglycosides among Pseudomonas aeruginosa isolates from patients with cystic fibrosis. *Antimicrob. Agents Chemother.* 48, 1676-1680.

Ziha-Zarifi, I., Llanes, C., Köhler, T., Pechere, J. C., and Plesiat, P. (1999). In vivo emergence of multidrug-resistant mutants of *Pseudomonas aeruginosa* overexpressing the active efflux system MexA-MexB-OprM. *Antimicrob. Agents Chemother.* 43, 287-291.
